# Dietary patterns in the progression of metabolic dysfunction-associated fatty liver disease to advanced liver disease: a prospective cohort study

**DOI:** 10.1016/j.ajcnut.2024.07.015

**Published:** 2024-07-17

**Authors:** Tengfei Li, Jianhui Zhao, Haoze Cao, Xin Han, Ying Lu, Fangyuan Jiang, Xinxuan Li, Jing Sun, Siyun Zhou, Zhongquan Sun, Weilin Wang, Yuan Ding, Xue Li

**Affiliations:** 1 Department of Hepatobiliary and Pancreatic Surgery, The Second Affiliated Hospital, Zhejiang University School of Medicine, Hangzhou, Zhejiang, China; 2 Department of Big Data in Health Science School of Public Health and The Second Affiliated Hospital, Zhejiang University School of Medicine, Hangzhou, Zhejiang, China; 3 Key Laboratory of Precision Diagnosis and Treatment for Hepatobiliary and Pancreatic Tumor of Zhejiang Province, Hangzhou, Zhejiang, China; 4 Research Center of Diagnosis and Treatment Technology for Hepatocellular Carcinoma of Zhejiang Province, Hangzhou, Zhejiang, China; 5 National Innovation Center for Fundamental Research on Cancer Medicine, Hangzhou, Zhejiang, China; 6 Cancer Center, Zhejiang University, Hangzhou, Zhejiang, China; 7 ZJU-Pujian Research & Development Center of Medical Artificial Intelligence for Hepatobiliary and Pancreatic Disease, Hangzhou, Zhejiang, China; 8 Centre for Global Health, Usher Institute, University of Edinburgh, Edinburgh, United Kingdom

**Keywords:** metabolic dysfunction-associated fatty liver disease, dietary pattern, chronic liver disease, severe liver disease, dietary intervention

## Abstract

**Background:**

Metabolic dysfunction-associated fatty liver disease (MAFLD) is a significant health problem. Dietary intervention plays an important role in patients with MAFLD.

**Objectives:**

We aimed to provide a reference for dietary patterns in patients with MAFLD.

**Methods:**

The presence of MAFLD was determined in the United Kingdom Biobank cohort. Nine dietary pattern scores were derived from the dietary records. Multivariable Cox regression models were used to estimate the hazard ratios (HRs) and 95% confidence intervals (CIs). The contrast test was employed to calculate the heterogeneity across MAFLD statuses.

**Results:**

We identified 175,300 patients with MAFLD at baseline. Compared with non-MAFLD, MAFLD was significantly associated with chronic liver disease (CLD) (HR: 3.48; 95% CI: 3.15, 3.84), severe liver disease (SLD) (HR: 2.87; 95% CI: 2.63, 3.14), liver cancer (HR: 1.93; 95% CI: 1.67, 2.23), and liver-related death (LRD) (HR: 1.93; 95% CI: 1.67, 2.23). In the overall cohort, the alternate Mediterranean diet (aMED) (HR_CLD_: 0.53; 95% CI: 0.37, 0.76; HR_SLD_: 0.52; 95% CI: 0.37, 0.72), planetary health diet (PHD) (HR_CLD_: 0.62; 95% CI: 0.47, 0.81; HR_SLD_: 0.65; 95% CI: 0.51, 0.83), plant-based low-carbohydrate diet (pLCD) (HR_CLD_: 0.65; 95% CI: 0.49, 0.86; HR_SLD_: 0.66; 95% CI: 0.51, 0.85), and healthful plant-based diet index (hPDI) (HR_CLD_: 0.63; 95% CI: 0.47, 0.84; HR_SLD_: 0.61; 95% CI: 0.47, 0.78) were associated with a lower risk of CLD and SLD. Additionally, unhealthful plant-based diet index (uPDI) was associated with increased risk of CLD (HR: 1.42; 95% CI: 1.09,1.85), SLD (HR: 1.50; 95% CI: 1.19, 1.90), and LRD (HR: 1.88; 95% CI: 1.28-2.78). The aforementioned associations remained consistently strong within the MAFLD subgroup while exhibiting less pronounced in the non-MAFLD group. However, no significant heterogeneity was observed across different MAFLD statuses.

**Conclusions:**

These findings highlight the detrimental effects of MAFLD on the development of subsequent liver diseases and the importance of dietary patterns in managing MAFLD.

## Introduction

Metabolic dysfunction-associated fatty liver disease (MAFLD) is a recently proposed term for the accumulation of excess fat in the liver, along with overweight/obesity, type 2 diabetes mellitus (T2DM), or the presence of ≥2 metabolic abnormalities indicating metabolic dysregulation [1]. Several studies have suggested that the prevalence of MAFLD exceeds one-third of the population [2]. Individuals with MAFLD face an increased risk of progression to steatohepatitis, ultimately leading to cirrhosis, liver failure, and liver cancer (LC) [3,4]. MAFLD is a serious public health challenge. Currently, lifestyle interventions such as adopting healthier dietary habits and increasing physical activity remain the cornerstone of treatment for MAFLD [5]. Nevertheless, there is ongoing debate about defining an appropriate and nutritious diet for patients with MAFLD.

Dietary patterns involve the quantification, variation, or combination of different foods and nutrients in the diet and their habitual frequency of consumption, offering a more precise and comprehensive measurement of the overall dietary intake [6]. Studies indicate that dietary indices, such as the alternate Mediterranean diet (aMED) [7] and low-carbohydrate diet (LCD) [8], are significantly associated with a reduced risk of developing MAFLD phenotypes. Moreover, a number of studies have indicated that the healthful plant-based diet may offer protection against nonalcoholic fatty liver disease (NAFLD) [9]. However, the sulfur microbial diet (SMD) had an adverse association with incident NAFLD [10]. Despite these associations, research on the efficacy of dietary patterns in preventing further progression of liver disease is limited. In addition, a number of newly proposed dietary patterns merit further investigation, such as the planetary health diet (PHD) [11]. Consequently, it is vital to conduct a comprehensive assessment of dietary patterns and their association with the risk of liver disease morbidity and mortality, particularly in patients with MAFLD.

In this prospective study, we used individual data from the United Kingdom Biobank to investigate the specific effects of different dietary patterns on the risk of developing and progressing liver disease and separately investigated the association across MAFLD statuses. We aimed to provide a reference for dietary interventions in patients with MAFLD.

## Methods

### Study population

**FIGURE 1:**
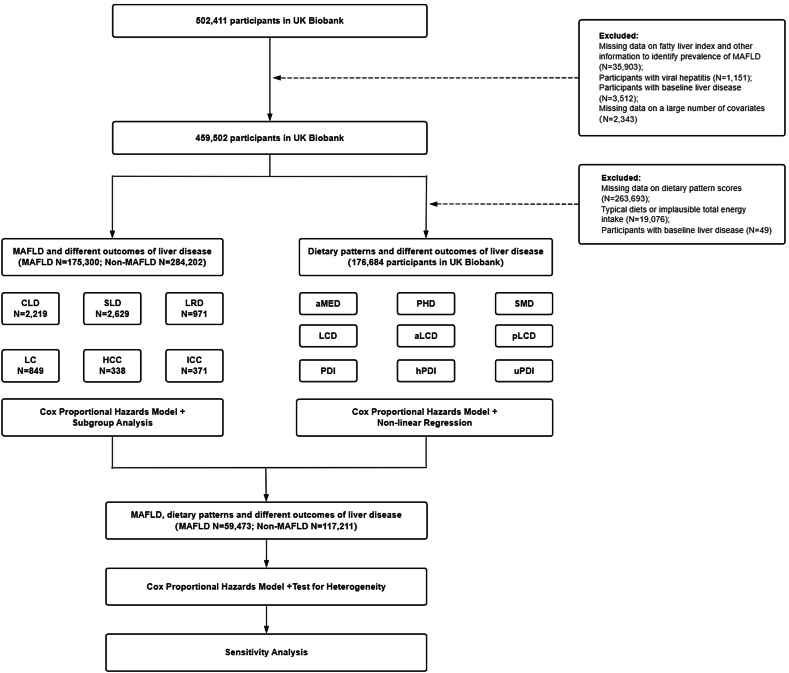
Study design and flowchart of the selection of eligible study population. aLCD, animal based low-carbohydrate diet; aMED, alternate Mediterranean diet; CLD, chronic liver disease; HCC, hepatocellular carcinoma; hPDI, healthful plant-based diet index; ICC, intrahepatic cholangiocarcinoma; LCD, low-carbohydrate diet; LC, liver cancer; LRD, liver-related death; MAFLD, metabolic dysfunction-associated fatty liver disease; PDI, plant-based diet index; PHD, planetary health diet; pLCD, plant-based low-carbohydrate diet; SMD, sulfur microbial diet; SLD, severe liver disease; uPDI, unhealthful plant-based diet index.

This study was conducted using data from all participants enrolled in the United Kingdom Biobank, a large-scale prospective cohort study that registered > 500,000 participants aged 40–69 y between 2006 and 2010 [12]. The United Kingdom Biobank documented phenotypic and genotypic health-related data linked to national medical records. All participants provided informed consent at the time of recruitment, allowing for follow-up using data linked to health records. United Kingdom Biobank has approval from the North West Multicenter Research Ethics Committee. This study was conducted in compliance with the principles of the Declaration of Helsinki. Figure 1 illustrates the process of constructing the analytical cohort.

### Diagnosis of MAFLD

According to the international expert consensus, the diagnostic criteria for MAFLD are evidence of fat accumulation in the liver through histological, imaging, or blood biomarker analyses, along with at least one of the following 3 criteria: overweight/obesity (BMI ≥25 kg/m^2^ in Caucasians or BMI ≥23 kg/m^2^ in Asians), presence of T2DM, or metabolic dysregulation [1]. In the absence of liver imaging or histological data in the United Kingdom Biobank database, the fatty liver index (FLI) was calculated to verify the presence of hepatic steatosis, with an FLI ≥ 60 indicating its likelihood [13]. The FLI is a well-established and commonly used indicator for fatty liver diagnosis and has been used in previous studies in the United Kingdom Biobank to identify hepatic steatosis [14,15]. The FLI is calculated using BMI, waist circumference, serum triglyceride concentrations, and λ-glutamyl transferase concentrations. For the identification of T2DM, individuals met any of the following criteria: *1)* glycated hemoglobin concentration above 47 mmol/mol; *2)* diagnosis codes ranging from E11.0 to E11.9, according to the International Classification of Diseases version 10 (ICD-10), or code 250 in ICD-9; or *3)* regularly used diabetes medication [16]. Metabolic dysregulation was defined as the presence of ≥2 of the following conditions: increased waist circumference, arterial hypertension, hypertriglyceridemia, low HDL cholesterol, prediabetes, insulin resistance, and subclinical inflammation [1]. Notably, insulin resistance was not assessed for metabolic dysfunction owing to the paucity of serum insulin data in the United Kingdom Biobank.

### Assessment of dietary patterns

The dietary factor data were obtained from the Oxford WebQ, a web-based, self-administered 24-h dietary questionnaire, which questioned the intake of 206 foods and 32 drinks within the previous 24 h. From April 2009 to June 2012, the United Kingdom Biobank invited participants to complete the Oxford WebQ, which showed good agreement between reported consumption at recruitment and at the repeat assessment center visit [17]. In order to reduce bias, participants with typical diets or implausible total energy intake were excluded in the study.

We calculated the following 9 dietary patterns based on previous studies to understand participants' adherence to different dietary patterns. Detailed descriptions of each dietary pattern are provided in Supplemental Table 1. The aMED score indicates the degree of adherence to the Mediterranean diet, comprising 9 different dietary components. Each component was scored as 0 (unhealthy) or 1 (healthy) based on whether the participant’s intake was above or below the cohort median level. [7]. The PHD was proposed in 2019 by the EAT-Lancet Commission as a beneficial diet for both people and planet [11]. The PHD score comprises 13 elements, each of which is scored as 0 (unhealthy) or 1 (healthy) according to whether the participant's intake meets the predetermined criteria [18]. The SMD score included 8 food groups that were linked to the abundance of sulfur-metabolizing bacterial species. The score was calculated by summing the product of beta-coefficients and the intake of corresponding foods. Higher scores indicated a more significant enrichment of sulfur-metabolizing bacteria [10]. The LCD score is determined by the relative amounts of fat, protein, and carbohydrate intake. The energy proportions of carbohydrate, protein, and fat were divided into 11 groups, with each group having an equal number of participants. These groups were assigned values ranging from 0 to 10 based on their ranking order. Participants in the highest stratum for fat and protein received 10 points, whereas those in the highest stratum for carbohydrate received 0 points. The scores for the 3 macronutrients were then summed to obtain the final LCD scores. Furthermore, 2 additional LCD scores, the animal based low-carbohydrate diet (aLCD) and the plant-based low-carbohydrate diet (pLCD) were created based on the source of protein and fat (animals or plants) [19]. The plant-based diet index (PDI) classifies the 18 food groups into 3 main groups: healthy plant foods, less healthy plant foods, and animal foods. It assigns positive weights to plant foods and negative weights to animal foods, and each group is assigned from 1 to 5 points, according to its intake quintile. Additionally, the healthful plant-based diet index (hPDI) and unhealthful plant-based diet index (uPDI) are derived by classifying plant foods based on their healthiness [9]. We calculated the cumulative means of the dietary pattern scores to capture the long-term dietary intake and diminish arbitrary within-person variability.

### Covariates assessment

We included the following factors as covariates: age (continuous), sex (male/female), race (white people/others), educational attainment (college degree/below college degree), Townsend deprivation index (TDI) (continuous), family history of cancer (yes/no), self-reported smoking status (never smoked/former or current smokers), drinking status (none-to-moderate/excessive) or average alcohol intake (continuous), physical activity (adequate/inadequate) or metabolic equivalent of task (MET) (continuous), average energy intake (continuous), BMI (continuous), waist-hip ratio (WHR) (continuous), and T2DM (yes/no).

In the study of the association between MAFLD and liver diseases, age was defined as age at recruitment; when examining the association between dietary patterns and liver diseases, age was defined as age at first completion of the Oxford WebQ. Average alcohol intake was calculated based on participants’ reported consumption of alcoholic beverages. Drinking status was divided into 2 groups based on daily alcohol consumption. For females, a consumption of 0–14 g per day and for males, 0–28 g per day were defined as none-to-moderate drinking. Conversely, consumption beyond these amounts is considered to be excessive. According to the International Physical Activity Questionnaire, we categorized physical activity into adequate (moderate activity 150 min/wk or vigorous activity ≥ 75 min/wk or equivalent combination or moderate physical activity ≥5 d per week or vigorous activity once per week) and inadequate (below adequate levels). Furthermore, we calculated the total METs by multiplying and summing the MET value of activity (3.3 METs for light physical activity, 4.0 METs for moderate physical activity, and 8.0 METs for vigorous physical activity) by the time of physical activity (minutes per week) [20]. Average energy intake was the cumulative means of five 24-h online dietary measurements. BMI and WHR were calculated based on measurements taken at the physical examination centers. We imputed the median for continuous variables or applied the most common category for categorical variables if covariate information was missing or recorded as “unknown.”

### Outcomes ascertainment

Cases of liver disease incidence and mortality were identified by linking the United Kingdom Hospital Episode Statistics data and the national death registries. The ICD-10 codes were used to identify cases of incident chronic liver disease (CLD), severe liver disease (SLD), and LC. CLD is defined as the occurrence of liver fibrosis or cirrhosis [21], whereas SLD encompasses a broader set of conditions and is defined as a composite diagnosis of cirrhosis, decompensated liver disease, hepatocellular carcinoma (HCC), and/or liver transplantation [22]. Furthermore, LC was categorized specifically as HCC or intrahepatic cholangiocarcinoma (ICC) based on ICD codes (Supplemental Table 2). Liver disease mortality was defined as death caused by liver disease (ICD-10 codes C22, I85, K70, K72, and K74-K76). Additionally, we identified prevalent liver diseases by consulting both the ICD-10 and ICD-9 codes (Supplemental Table 3). To ensure the accuracy of our cohort data, we excluded participants with a documented history of liver disease at baseline.

### Statistical analysis

Continuous and categorical variables are presented as mean and frequency (percentage), respectively. Person-years of follow-up were calculated from the date of initial recruitment to the United Kingdom Biobank when studying the association between MAFLD and liver diseases or the initial completion of the online dietary assessment when investigating various dietary patterns in relation to outcomes until the earliest diagnosis of liver disease, death, or end of follow-up (20 September, 2022).

Cox proportional hazard regression models were used to calculate the adjusted hazard ratios (HRs) and 95% confidence intervals (CIs) to evaluate the association between MAFLD and the risk of morbidity and mortality related to liver disease. We test the proportional hazards assumption using Kaplan-Meier estimation and the scaled Schoenfeld residuals, although no clear violations are observed. We constructed 2 distinct models for our analyses: Model 1, which was adjusted for age at recruitment and sex, and Model 2, which was further adjusted for variables, including race, educational attainment, TDI, family history of cancer, smoking status, drinking status, and physical activity. Subgroup analyses were conducted to explore the potential interactive factors. These analyses stratified participants by sex, age, educational level, alcohol consumption status, and smoking status.

The cumulative averages of dietary pattern scores were calculated as the mean of all online assessments completed by the participants. Pearson correlation coefficients (r) were calculated to measure the correlation between dietary pattern scores. Cox proportional hazards regression models were applied to estimate the likelihood of morbidity and mortality related to liver disease across dietary pattern scores. The dietary pattern scores were categorized into quintiles and treated as categorical variables. To detect the trends, we assigned the median value of each quintile as a continuous variable in the regression model. The 2 models were constructed in a similar manner. Model 2 was adjusted for age at first completion of the Oxford WebQ, sex, race, educational attainment, TDI, family history of cancer, smoking status, average alcohol intake (except aMED & SMD), MET, average energy intake, BMI, WHR, and T2DM. A multiple comparison test based on the false discovery rate (FDR) method was employed to assess the association between different dietary patterns and outcomes. Restricted cubic spline (RCS) analysis was used to evaluate the potential nonlinear associations between dietary pattern scores. The number of knots was chosen based on the smallest Akaike information criterion value, thus optimizing the model fit [23]. The overall significance of the spline curves was verified using a likelihood ratio test.

Furthermore, we divided the participants into 2 categories based on their MAFLD status and investigated the relationship between dietary patterns and liver disease risk within each group. We employed the contrast test to calculate the heterogeneity across MAFLD statuses to determine whether the associations between dietary exposure and disease risk varied between individuals with and without MAFLD [24].

Several sensitivity analyses were performed to ensure robustness of the findings. These analyses included *1)* excluding individuals newly diagnosed with MAFLD during the study period, *2)* excluding participants of other races, and *3)* excluding outcome events that occurred within the first 2 years of follow-up to minimize the potential influence of pre-existing conditions or reverse causality on the observed associations. All statistical analyses were performed using R version 4.2.1. Statistical significance was set at a 2-sided *P* value <0.05.

## Results

### Characteristics of the study population

A total of 459,502 individuals were included in the analysis, comprising 175,300 patients with MAFLD and 284,202 participants without MAFLD. The prevalence of MAFLD was 38.15%. The baseline characteristics of the study participants are shown in Table 1. Compared with individuals without MAFLD, those with MAFLD had higher rates of older age, male, overweight, hypertension, T2DM, alcohol and tobacco use, and lower socioeconomic status, educational level, and physical activity. In addition, patients with MAFLD had higher concentrations of liver enzymes, C-reactive protein, triglycerides, and cholesterol but lower concentrations of HDL cholesterol.

**TABLE 1:** Baseline characteristics of participants with MAFLD and without MAFLD in the United Kingdom Biobank cohort.

	Overall	MAFLD	Non-MAFLD
Sample size (No.)	459,502	175,300	284,202
Age at recruitment, year (SD)	56.53 (8.09)	57.29 (7.83)	56.06 (8.22)
Sex, *n* (%)			
Female	249,537 (54.3)	63,799 (36.4)	185,738 (65.4)
Male	209,965 (45.7)	111,501 (63.6)	98,464 (34.6)
Race, *n* (%)			
White	435,632 (94.8)	166,103 (94.8)	269,529 (94.8)
Others	23,870 (5.2)	9197 (5.2)	14,673 (5.2)
TDI scores (SD)	−1.34 (3.06)	−1.07 (3.18)	−1.51 (2.98)
Education, *n* (%)		··	
College degree	149,326 (32.5)	46,658 (26.6)	102,668 (36.1)
Below college degree	310,176 (67.5)	128,642 (73.4)	181,534 (63.9)
Smoking status, *n* (%)			
Never smoked	252,733 (55.0)	85,025 (48.5)	167,708 (59.0)
Former or current smokers	206,769 (45.0)	902,75 (51.5)	116,494 (41.0)
Drinking status, *n* (%)			··
None-to-moderate	338,922 (73.8)	125,964 (71.9)	212,958 (74.9)
Excess	120,580 (26.2)	49,336 (28.1)	71,244 (25.1)
Physical activity, *n* (%)			
Adequate	320,233 (69.7)	111,106 (63.4)	209,127 (73.6)
Inadequate	139,269 (30.3)	64,194 (36.6)	75,075 (26.4)
Family history of cancer, *n* (%)			
No	336,051 (73.1)	127,648 (72.8)	208,403 (73.3)
Yes	123,451 (26.9)	47,652 (27.2)	75,799 (26.7)
BMI, kg/m^2^ (SD)	27.40 (4.76)	31.47 (4.44)	24.90 (2.84)
WHR, scores (SD)	0.87 (0.09)	0.94 (0.07)	0.83 (0.07)
Hypertension, *n* (%)	328,306 (71.4)	146,687 (83.7)	181,619 (63.9)
Type 2 diabetes, *n* (%)	43,129 (9.4)	23,598 (13.5)	19,531 (6.9)
FLI, scores (SD)	47.98 (30.09)	81.13 (11.52)	27.52 (16.91)
ALP, U/L (SD)	83.46 (25.72)	88.51 (28.16)	80.35 (23.56)
ALT, U/L (SD)	23.46 (13.89)	30.00 (17.02)	19.43 (9.53)
AST, U/L (SD)	26.14 (10.28)	28.70 (12.58)	24.57 (8.17)
Cholesterol, mmol/L (SD)	5.70 (1.14)	5.71 (1.22)	5.69 (1.09)
CRP, mg/L (SD)	2.58 (4.32)	3.62 (4.83)	1.95 (3.84)
GGT, U/L (SD)	37.05 (40.92)	54.75 (56.50)	26.13 (20.64)
HDL cholesterol, mmol/L (SD)	1.45 (0.38)	1.25 (0.30)	1.57 (0.38)
Triglycerides, mmol/L (SD)	1.75 (1.03)	2.40 (1.20)	1.34 (0.62)

Abbreviations: ALP, alkaline phosphatase; ALT, alanine aminotransferase; AST, aspartate aminotransferase; CRP, C-reactive protein; FLI, fatty liver index; GGT, λ-glutamyl transferase; MAFLD, metabolic dysfunction-associated fatty liver disease; SD, standard deviation; TDI, Townsend deprivation index; WHR, waist-hip ratio.

### MAFLD and risk of liver disease morbidity and mortality

After a mean follow-up of 13.65 years, a total of 2219 incident cases of CLD, 2629 incident cases of SLD, 849 incident cases of LC, and 971 cases of mortality due to liver disease were identified. Information pertaining to liver diseases among the participants with and without MAFLD is shown in Supplemental Table 4.

**FIGURE 2:**
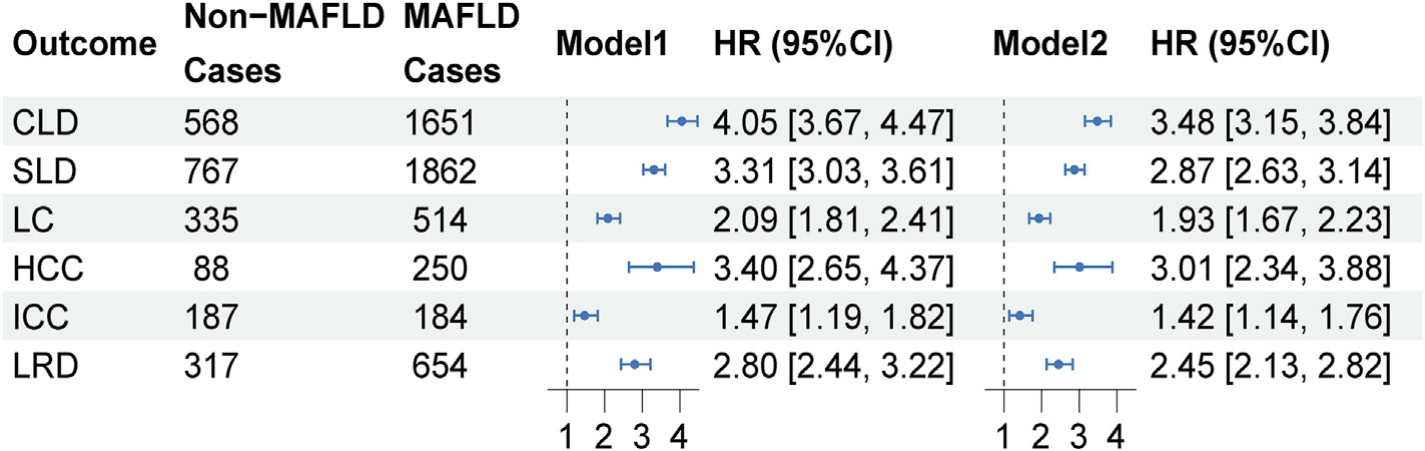
Associations of MAFLD with risk of liver disease morbidity and mortality in the United Kingdom Biobank cohort. The Cox regression model derived HRs, 95% CIs, and *P* values by using participants without MAFLD as the reference group. Model 1 was adjusted for age at recruitment and sex, and Model 2 was further adjusted for race, TDI, smoking and drinking status, educational attainment, physical activity, and family history of cancer. CI, confidence interval; CLD, chronic liver disease; HCC, hepatocellular carcinoma; HR, hazard ratio; ICC, intrahepatic cholangiocarcinoma; LC, liver cancer; LRD, liver-related death; MAFLD, metabolic dysfunction-associated fatty liver disease; SLD, severe liver disease.

The associations between MAFLD and the risk of incident CLD, SLD, LC, and its subtypes, and the risk of liver-related death (LRD) are shown in Figure 2. Patients with MAFLD showed increased risk of CLD (HR: 3.48; 95% CI: 3.15, 3.84), SLD (HR: 2.87; 95% CI: 2.63, 3.14), and LC (HR: 1.93; 95% CI: 1.67, 2.23) in comparison with those without MAFLD. When examining the associations between MAFLD and the subtypes of LC, the analysis revealed a consistent and significant association between MAFLD and HCC (HR: 3.01; 95% CI: 2.34, 3.88), as well as MAFLD and ICC (HR: 1.42; 95% CI: 1.14, 1.76). Additionally, MAFLD poses a notable risk for mortality caused by liver disease (HR: 2.45; 95% CI: 2.13, 2.82).

Subgroup analyses (Supplemental Table 5) revealed that the associations of MAFLD with CLD (HR for the MAFLD ∗ Male: 0.66; 95% CI: 0.54, 0.80) and SLD (HR for the MAFLD ∗ Male: 0.74; 95% CI: 0.62, 0.88) were more pronounced in females than in males. Conversely, the associations between MAFLD and LC (HR for the MAFLD ∗ Male: 1.61; 95% CI: 1.19, 2.17) and between MAFLD and HCC (HR for the MAFLD ∗ Male: 2.07; 95% CI: 1.21, 3.56) were stronger in males. The associations between MAFLD and LC (HR for the MAFLD ∗ Excess: 1.45; 95% CI: 1.06, 1.99), HCC (HR for the MAFLD ∗ Excess: 2.47; 95% CI: 1.34, 4.55), and LRD (HR for the MAFLD ∗ Excess: 1.34; 95% CI: 1.01, 1.77) were strengthened in heavy drinkers. Additionally, the association between MAFLD and CLD was more significant in never smokers (HR for the MAFLD ∗ Previous or current smokers: 0.82; 95% CI: 0.68, 1.00). The analysis demonstrated that the associations between MAFLD and the risk of liver disease morbidity and mortality remained consistent across different age groups and education levels (all *P* interactions > 0.164).

### The scores of different dietary patterns in the study population

Within the subset of participants who provided complete dietary questionnaire data, we included 59,473 patients with MAFLD and 117,211 individuals without MAFLD in our analysis. Table 2 shows the average scores for the 9 dietary patterns of the participants. Those diagnosed with MAFLD exhibited greater energy consumption and higher scores for potentially unhealthy dietary patterns such as SMD, LCD, aLCD, and uPDI. In contrast, participants without MAFLD had higher scores for metabolic equivalents and potentially healthy dietary patterns, including aMED, PHD, pLCD, PDI, and hPDI. The correlation between LCD and aLCD was strong (r = 0.9) but weaker for the other dietary patterns (Supplemental Figure 1).

**TABLE 2:** The average dietary pattern scores of participants with MAFLD and without MAFLD in the United Kingdom Biobank cohort.

	Score Range	Overall	MAFLD	Non-MAFLD
Sample size (No.)		176,684	59,473	117,211
Age^1^, year (SD)		58.47 (7.98)	59.31 (7.70)	58.05 (8.08)
aMED, scores (SD)	0–9	3.55 (1.42)	3.33 (1.38)	3.66 (1.42)
PHD, scores (SD)	0–13	3.39 (1.15)	3.24 (1.14)	3.47 (1.15)
SMD, scores (SD)	−12.935 to 7.17^2^	−1.24 (1.10)	−1.15 (1.20)	−1.29 (1.04)
LCD, scores (SD)	0–30	9.01 (5.56)	9.31 (5.71)	8.86 (5.48)
aLCD, scores (SD)	0–30	7.70 (5.79)	8.19 (6.01)	7.46 (5.67)
pLCD, scores (SD)	0–30	16.31 (4.53)	16.04 (4.58)	16.44 (4.51)
PDI, scores (SD)	18–90	50.31 (5.17)	49.61 (5.27)	50.66 (5.07)
hPDI, scores (SD)	18–90	56.82 (5.74)	55.56 (5.67)	57.46 (5.67)
uPDI, scores (SD)	18–90	56.80 (5.71)	57.36 (5.80)	56.51 (5.64)
Alcohol intake, g/day (SD)		14.54 (19.93)	17.09 (23.58)	13.24 (17.65)
Energy intake, kJ (SD)		8595.81 (2296.16)	8862.58 (2421.31)	8460.45 (2217.74)
MET, scores (SD)		11.90 (8.83)	11.28 (9.03)	12.22 (8.71)

Abbreviations: aLCD, animal based low-carbohydrate diet; aMED, alternate Mediterranean diet; hPDI, healthful plant-based diet index; kJ, kilojoule; LCD, low-carbohydrate diet; MAFLD, metabolic dysfunction-associated fatty liver disease; MET, metabolic equivalent of task PDI, plant-based diet index; PHD, planetary health diet; pLCD, plant-based low-carbohydrate diet; SD, standard deviation; SMD, sulfur microbial diet; uPDI, unhealthful plant-based diet index.

1 Age was calculated by subtracting the date of first completion of the dietary questionnaire from the birthday.

2 The SMD was calculated by summing the product of beta-coefficients and the intake of corresponding food groups, with no specific score range. The range of SMD scores for all participants was presented in the table.

### Dietary patterns and risk of liver disease morbidity and mortality

**FIGURE 3:**
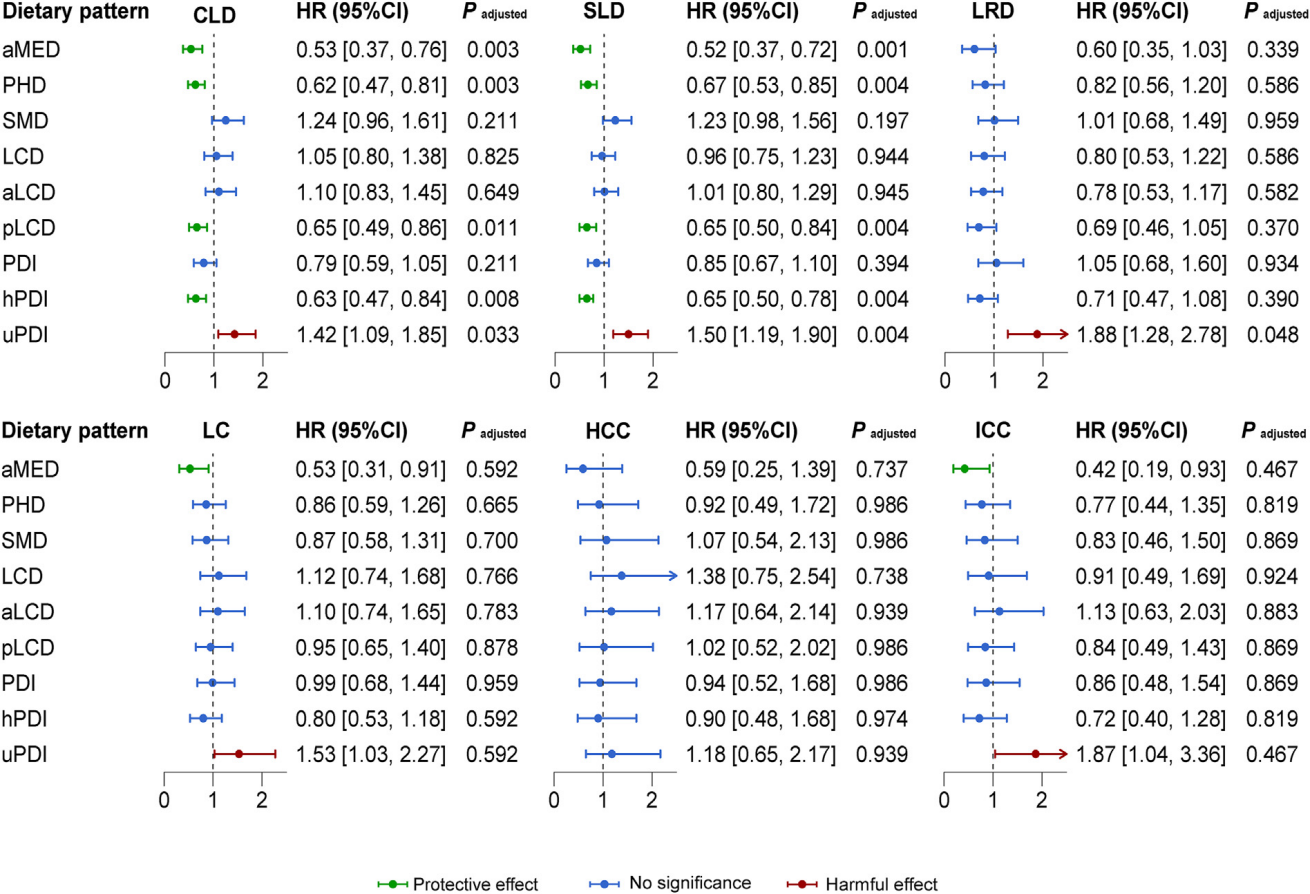
Associations of dietary pattern scores with risk of liver disease morbidity and mortality in the United Kingdom Biobank cohort. The 9 dietary pattern scores were divided into quintiles and treated as categorical variables. The Cox regression model derived HRs, 95% CIs, and *P* values by comparing the fifth quantile to the first quantile. The model was adjusted for age at first completion of the Oxford WebQ, sex, race, educational attainment, TDI, family history of cancer, smoking status, average alcohol intake (except aMED & SMD), MET, average energy intake, BMI, WHR, and T2DM. The *P* adjusted was determined using a multiple comparison test based on the false discovery rate (FDR). aLCD, animal based low-carbohydrate diet; aMED, alternate Mediterranean diet; CI, confidence interval; CLD, chronic liver disease; HCC, hepatocellular carcinoma; HR, hazard ratio; hPDI, healthful plant-based diet index; ICC, intrahepatic cholangiocarcinoma; LC, liver cancer; LCD, low-carbohydrate diet; LRD, liver-related death; PDI, plant-based diet index; PHD, planetary health diet; pLCD, plant-based low-carbohydrate diet; SLD, severe liver disease; SMD, sulfur microbial diet; uPDI, unhealthful plant-based diet index.

A mean follow-up of 11.49 y was observed, during which time a total of 562 incident cases of CLD, 686 incident cases of SLD, 257 incident cases of LC, and 237 cases of LRD were identified within the participants with complete dietary records. The associations between the scores of 9 dietary patterns and the risk of liver disease morbidity and mortality showed significant variations across different dietary patterns (Figure 3). The aMED (HR for the first to fifth quantile difference: 0.53; 95% CI: 0.37, 0.76), PHD (HR: 0.62; 95% CI: 0.47, 0.81), pLCD (HR: 0.65; 95% CI: 0.49, 0.86), and hPDI (HR: 0.63; 95% CI: 0.47, 0.84) were strongly associated with a lower risk of CLD. Similarly, these dietary patterns were also found to be associated with a decreased risk of SLD (HR of aMED: 0.52; 95% CI: 0.37, 0.72; HR of PHD: 0.65; 95% CI: 0.51, 0.83; HR of pLCD: 0.66; 95% CI: 0.51, 0.85; and HR of hPDI: 0.61; 95% CI: 0.47, 0.78). Furthermore, uPDI was associated with increased risk of CLD (HR: 1.42; 95% CI: 1.09, 1.85) and SLD (HR: 1.50; 95% CI: 1.19, 1.90) (Supplemental Table 6). Notably, uPDI was also associated with the risk of LRD (HR: 1.88; 95% CI: 1.28, 2.78), and the associations of other dietary patterns with LRD were nonsignificant (Supplemental Table 7). Overall, the aforementioned associations remained statistically significant after correcting for the FDR (*P* adjusted < 0.05). Regarding LC, a higher aMED score was associated with a decreased risk of LC (HR: 0.53; 95% CI: 0.31, 0.91), whereas the higher uPDI score, the higher risk of LC (HR: 1.53; 95% CI: 1.03, 2.27) (Supplemental Table 7). In an independent analysis of HCC and ICC, we only found a consistent association with ICC (HR of aMED: 0.42; 95% CI: 0.19, 0.93; HR of uPDI: 1.87; 95% CI: 1.04, 3.36) (Supplemental Table 8). However, the *P* adjusted exceeded 0.05 when the FDR-based multiple comparison was employed.

The results of the RCS analysis showed a statistically significant nonlinear relationship of LCD with CLD (*P* nonlinearity =  0.001) and SLD (*P* nonlinearity =  0.005). Additionally, significant nonlinearity was observed between PDI and SLD (*P* nonlinearity =  0.037) and between PDI and LC (*P* nonlinearity =  0.046) (Supplemental Table 9). The RCS curve for the association between LCD and CLD demonstrated the lowest HR of 0.62 (95% CI: 0.47, 0.81) at a score of 14.47. As for SLD, the RCS curves of LCD and PDI indicated the smallest HR at scores of 14.47 and 56.17, with HRs of 0.67 (95% CI: 0.52, 0.85) and 0.76 (95% CI: 0.59, 0.97), respectively. For the association between PDI and LC, the RCS curve presented the lowest HR of 1.00 (95% CI: 0.99, 1.00) at a PDI score of 50.19 (Supplemental Figure 2).

### Dietary patterns and risk of liver disease morbidity and mortality according to MAFLD status

**FIGURE 4:**
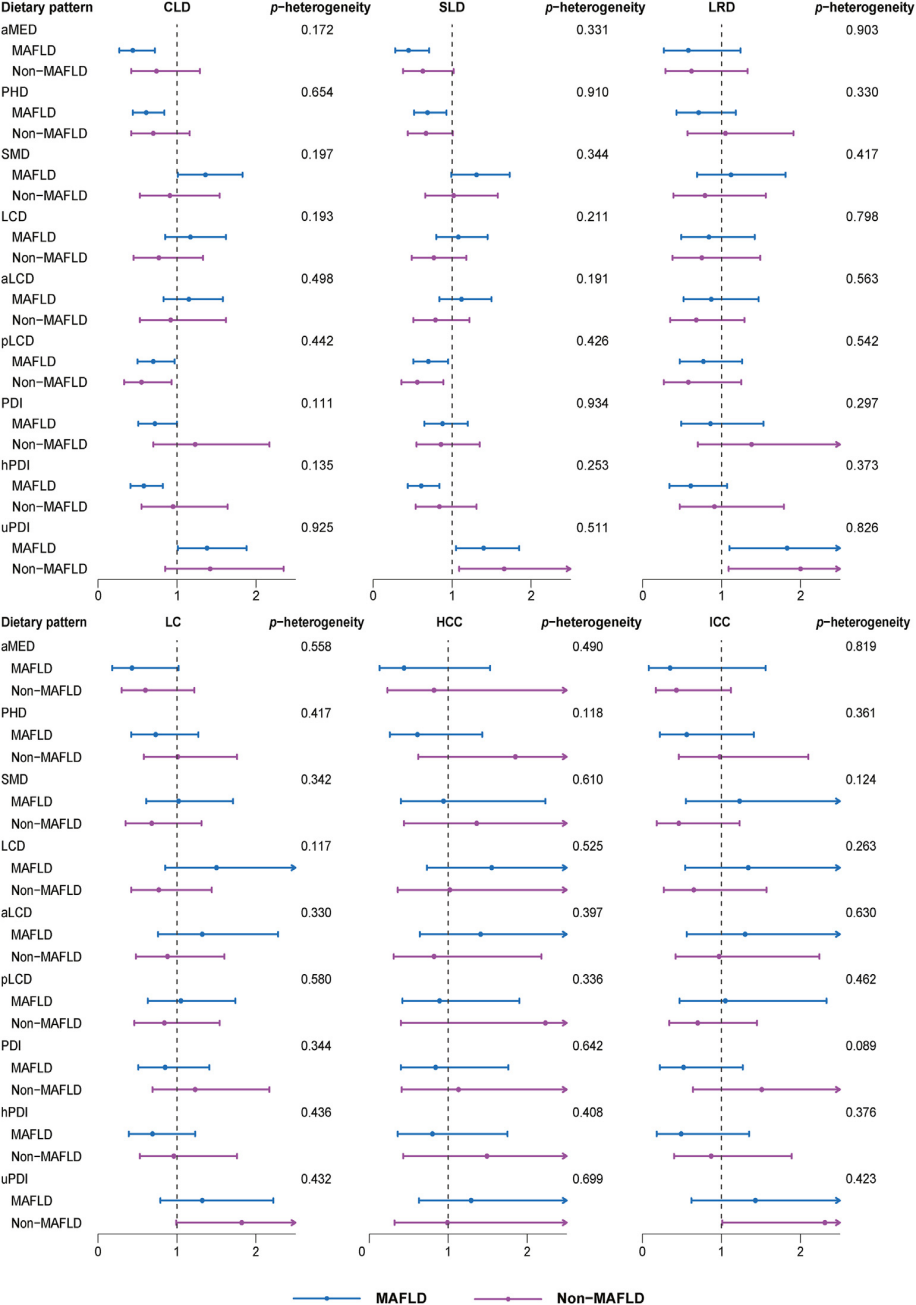
Associations of dietary pattern scores with risk of liver disease morbidity and mortality across MAFLD statuses. The 9 dietary pattern scores were divided into quintiles and treated as categorical variables. The Cox regression model derived HRs, 95% CIs, and *P* values by comparing the fifth quantile to the first quantile. The model was adjusted for age at first completion of the Oxford WebQ, sex, race, educational attainment, TDI, family history of cancer, smoking status, average alcohol intake (except aMED & SMD), MET, average energy intake, BMI, WHR, and T2DM. The *P* heterogeneity was determined using the contrast method based on a fully unconstrained approach. aLCD, animal based low-carbohydrate diet; aMED, alternate Mediterranean diet; CLD, chronic liver disease; HCC, hepatocellular carcinoma; hPDI, healthful plant-based diet index; ICC, intrahepatic cholangiocarcinoma; LC, liver cancer; LCD, low-carbohydrate diet; LRD, liver-related death; MAFLD, metabolic dysfunction-associated fatty liver disease; PDI, plant-based diet index; PHD, planetary health diet; pLCD, plant-based low-carbohydrate diet; SLD, severe liver disease; SMD, sulfur microbial diet; uPDI, unhealthful plant-based diet index.

To investigate the potential impact of MAFLD on the association between dietary pattern scores and the risk of liver diseases morbidity and mortality, we stratified the cohort into subgroups with and without MAFLD. The results revealed that within the MAFLD subgroup, the correlation between dietary pattern scores and the risk of CLD and SLD remained remarkably consistent with those observed in the overall cohort. However, this association was less pronounced in the non-MAFLD group, as indicated by the *P* values. For participants without MAFLD, pLCD had a protective effect against CLD (HR: 0.55; 95% CI: 0.33,0.93) and SLD (HR: 0.56; 95% CI: 0.36, 0.89). Conversely, an increased risk of SLD was noted with uPDI (HR: 1.66; 95% CI: 1.09, 2.53). When examining the associations between dietary pattern scores and the risk of LC and its subtypes, the protective effects of aMED on LC and ICC were less substantial in both MAFLD and non-MAFLD subgroups. Nonetheless, a high risk of uPDI associated with ICC remained in the participants without MAFLD (HR: 2.31; 95% CI: 1.01, 5.30). Regarding LRD, the risk of uPDI was consistent in both groups (Figure 4 and Supplemental Tables 10–15). Concurrently, as we analyzed heterogeneity across MAFLD statuses, we found no significant heterogeneity (*P* heterogeneity > 0.089) in the association between various dietary patterns and subsequent liver disease (Figure 4).

### Sensitivity analysis

According to the sensitivity analysis, the associations between MAFLD and incident CLD, SLD, LC, HCC, ICC, and LRD remained stable even after excluding individuals newly diagnosed with MAFLD, participants of other races, and participants with outcome events that occurred within the first 2 years of follow-up (Supplemental Table 16). Likewise, the relationship between dietary pattern scores and the risk of these liver diseases remained unchanged (Supplemental Tables 17 and 18).

## Discussion

In this large-scale cohort study, we investigated the relationship between MAFLD and the incidence and mortality of liver diseases. Additionally, we assessed the influence of different dietary patterns on it. After adjusting for multiple confounding factors, MAFLD was found to be significantly associated with a higher risk of developing various liver diseases and an increased risk of LRD. According to the evaluation of 9 dietary patterns across the overall population, aMED, PHD, pLCD, and hPDI were beneficial for reducing the risk of CLD and SLD. Conversely, uPDI appeared to increase these risks. Additionally, uPDI was associated with a higher risk of LC and LRD. A consistent association between dietary patterns and the incidence and mortality of liver diseases was also observed in the MAFLD subgroup. However, some of these relationships were nonsignificant among the participants without MAFLD. To our knowledge, this study is the first to systematically examine the effectiveness of different dietary patterns on the risk of liver disease morbidity and mortality in patients with MAFLD.

MAFLD is a recent term debated within the field of hepatology but has shown a tendency to gain significant global recognition and support [25,26]. MAFLD integrates a group of metabolic ailments within the framework of hepatic steatosis, and a few studies indicate that the MAFLD classification is more effective in identifying individuals with metabolically complex fatty liver than the traditional NAFLD criteria. Although we recognize that the same dietary recommendations are suitable for patients with NAFLD and MAFLD, it may be advantageous to re-evaluate the definition of disease in people with worsening metabolic well-being.

Previous studies reported that aMED and LCD are associated with a lower risk of MAFLD [7,8]. And hPDI may have a protective effect against NAFLD [9]. Some guidelines emphasize the significance of caloric restriction in NAFLD treatment, and some associations recommend aMED [27,28]. The results of our research are in line with those of previous studies, indicating greater benefits of aMED, pLCD, and hPDI in patients with MAFLD. Additionally, we found that PHD may also be beneficial for patients with MAFLD. Furthermore, we identified uPDI as a harmful dietary pattern that could increase the risk of subsequent liver disease. This study provided initial evidence that some dietary patterns may help prevent MAFLD from progressing to more SLD.

The development and progression of MAFLD are multifactorial and involve interactions between various factors, including lifestyle, dietary habits, and individual genetics. In terms of dietary influence, excessive calorie intake and dietary patterns rich in saturated fats, carbohydrates, and sugar-sweetened beverages have been implicated in the development of liver steatosis [29]. Over intake of fat and carbohydrates leads to the accumulation of free fatty acids, and excess fatty acids increase endoplasmic reticulum stress, oxidative stress, and activation of inflammatory factors [30]. Furthermore, excessive consumption of saturated fatty acids may result in hepatic gluconeogenesis, insulin resistance, and lipid accumulation in the liver [31], which contributes to oxidative damage leading to necroinflammation [32]. Additionally, diet can affect liver fat deposition by regulating overall adiposity. Consumption of certain food components, such as fruits and vegetables, may decrease energy intake and increase the production of beneficial short-chain fatty acids, which can aid in suppressing inflammation and weight loss [33]. Notably, bacteria could be future targets for treating MAFLD as the liver-gut axis is a key focus of therapy [34]. Hydrogen sulfide, which is produced by gut microorganisms, has been associated with increased intestinal permeability and inflammation. Studies revealed that SMD is associated with a greater incidence of obesity and NAFLD [35,10]. Meanwhile, it has been discovered that an increase of 1 SD in SMD is associated with an increased risk of CLD (HR: 1.12; 95% CI: 1.03, 1.21) and SLD (HR: 1.10; 95% CI: 1.02, 1.18) in our study (Supplemental Table 6). Hence, it is plausible that SMD plays a significant role in the elevated risk of liver disease associated with MAFLD. In conclusion, to enhance the quality of life and prevent the development of other liver diseases in patients with MAFLD, it is imperative to promptly evaluate patients' nutritional status and provide them with adequate dietary interventions.

The strengths of this study include the large sample size, prospective design, and long-term follow-up. Additionally, comprehensive measurements of blood-related biomarkers and detailed dietary questionnaire data from the United Kingdom Biobank enabled the identification of MAFLD and assessment of the impact of different dietary patterns on the progression from MAFLD to other liver diseases. However, our study had several limitations. First, although we investigated 9 different dietary patterns and their effects on 6 outcomes in patients with MAFLD, it is possible that our study overlooked certain aspects due to the broad range of definitions of dietary patterns. Furthermore, we did not consider the potential risks of cardiovascular disease or all-cause mortality in patients with MAFLD. Second, although the ethnic and socioeconomic backgrounds of individuals in the UK Biobank were diverse, the participants in these studies were predominantly white, which may have limited the generalizability and transferability of the results. Finally, the possibility of residual confounding bias cannot be ruled out despite adjustment for the most common health-related confounders. Overall, it is important to conduct future randomized controlled trials and explore the physiological mechanisms to verify the impact of alterations in each dietary pattern on the progression of severe liver damage in patients with MAFLD.

## Acknowledgments

This research was conducted using the United Kingdom Biobank study under Application Number 66354. We thank the United Kingdom Biobank Principal Investigator and Chief Executive Sir Rory Collins and all the United Kingdom Biobank participants, researchers, and staff.

### Author contributions

The authors’ responsibilities were as follows – Xue Li, YD, WW: conceptualized the project; TL, JZ: performed the data analyses and wrote the first draft; HC, XH, YL, FJ, Xinxuan Li, JS, SZ, ZS: helped with the review and editing; Xue Li, YD, WW: revised the final version of the manuscript; and all authors: read and approved the final manuscript.

### Conflict of interest

The authors report no conflicts of interest.

### Funding

YD was supported by the Key Project of Traditional Chinese Medicine Science and Technology Plan of Zhejiang Province (GZY-ZJ-KJ-24077) and the National Natural Science Foundation of China (No. 82001673 and No. 82272860); WLW was supported by the Key Research and Development Program of Zhejiang Province (No. 202121C03121), the National Natural Science Foundation of China (No. 82072650) and the Zhejiang University Basic Research Fund (No. 226-2022-00037).

### Ethics approval

This prospective cohort study was conducted based on the United Kingdom Biobank (approved by the North West Multicenter Research Ethics Committee).

### Reporting checklist

The authors have completed the STROBE reporting checklist.

### Data availability

Only publicly available data were used in this study, and the data sources and handling methods are described in the Methods section. The United Kingdom Biobank study was under Application Number 66354. The United Kingdom Biobank is an open access resource, and bona fide researchers can apply it to the United Kingdom Biobank dataset by registering at http://ukbiobank.ac.uk/register-apply/. Additional information is available from the corresponding author upon request.
