# Supplementary material for: Dietary patterns in the progression of metabolic dysfunction-associated fatty liver disease to advanced liver disease: a prospective cohort study
Source: Am J Clin Nutr. 2024 Jul 17;120(3):518–27. doi: 10.1016/j.ajcnut.2024.07.015 (PMC11393393; doi:10.1016/j.ajcnut.2024.07.015)
Supplement: Multimedia component 1 [file mmc1.docx]

**Dietary patterns in the progression of metabolic dysfunction-associated fatty liver disease to advanced liver disease: a prospective cohort study**

**First Authors:** Tengfei Li, Jianhui Zhao

**Supplementary figures**

- Figure S1. The Pearson correlation coefficients between the dietary pattern scores in the UK Biobank cohort.
- Figure S2. The nonlinear associations between the dietary pattern scores and risk of CLD, SLD and LC in the UK Biobank cohort.

**Supplementary Tables**

- Table S1. Detailed descriptions of dietary patterns.
- Table S2. Criteria for endpoint events of different liver diseases.
- Table S3. ICD-9 & ICD-10 codes used to define baseline liver disease.
- Table S4. The associations of MAFLD with the incidence and mortality of liver diseases in the UK Biobank cohort.
- Table S5. Subgroup analyses for associations of MALFD with the incidence and mortality of liver diseases.
- Table S6. The associations of the scores of dietary patterns with the incidence of chronic liver disease and severe liver disease in the UK Biobank cohort.
- Table S7. The associations of the scores of dietary patterns with the incidence of liver cancer and mortality of liver diseases in the UK Biobank cohort.
- Table S8. The associations of the scores of dietary patterns with the incidence of hepatocellular carcinoma and intrahepatic cholangiocarcinoma in the UK Biobank cohort.
- Table S9. The *p*-nonlinearity of the nonlinear associations between dietary pattern scores and the incidence and mortality of liver diseases.
- Table S10. The associations of the scores of dietary patterns with the incidence of chronic liver disease according to MAFLD status.
- Table S11. The associations of the scores of dietary patterns with the incidence of severe liver disease according to MAFLD status.
- Table S12. The associations of the scores of dietary patterns with the incidence of liver cancer according to MAFLD status.
- Table S13. The associations of the scores of dietary patterns with the incidence of hepatocellular carcinoma according to MAFLD status.
- Table S14. The associations of the scores of dietary patterns with the incidence of intrahepatic cholangiocarcinoma according to MAFLD status.
- Table S15. The associations of the scores of dietary patterns with the mortality of liver disease according to MAFLD status.
- Table S16. Sensitivity analyses for associations of MAFLD with the incidence and mortality of liver diseases in the UK Biobank cohort.
- Table S17. Sensitivity analyses for associations of the scores of dietary patterns with the incidence and mortality of liver diseases in the UK Biobank cohort.
- Table S18. Sensitivity analyses for associations of the scores of dietary patterns with the incidence of liver cancer and its subtypes in the UK Biobank cohort.


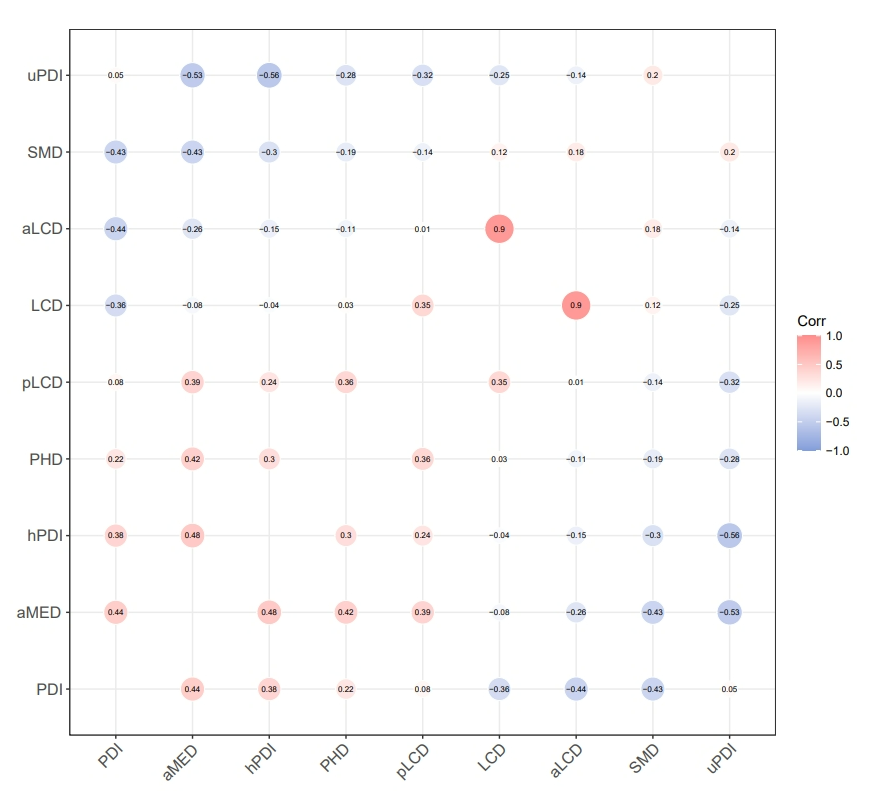


**Figure S1. The Pearson correlation coefficients between the dietary pattern scores in the UK Biobank cohort.**

uPDI, unhealthful Plant-based Diet Index; SMD, sulfur microbial diet; aLCD, animal based Low-carbohydrate Diet; LCD, Low-carbohydrate Diet; pLCD, plant based Low-carbohydrate Diet; PHD, Planetary Health Diet; hPDI, healthful Plant-based Diet Index; aMED, alternate Mediterranean diet; PDI, Plant-based Diet Index.

**
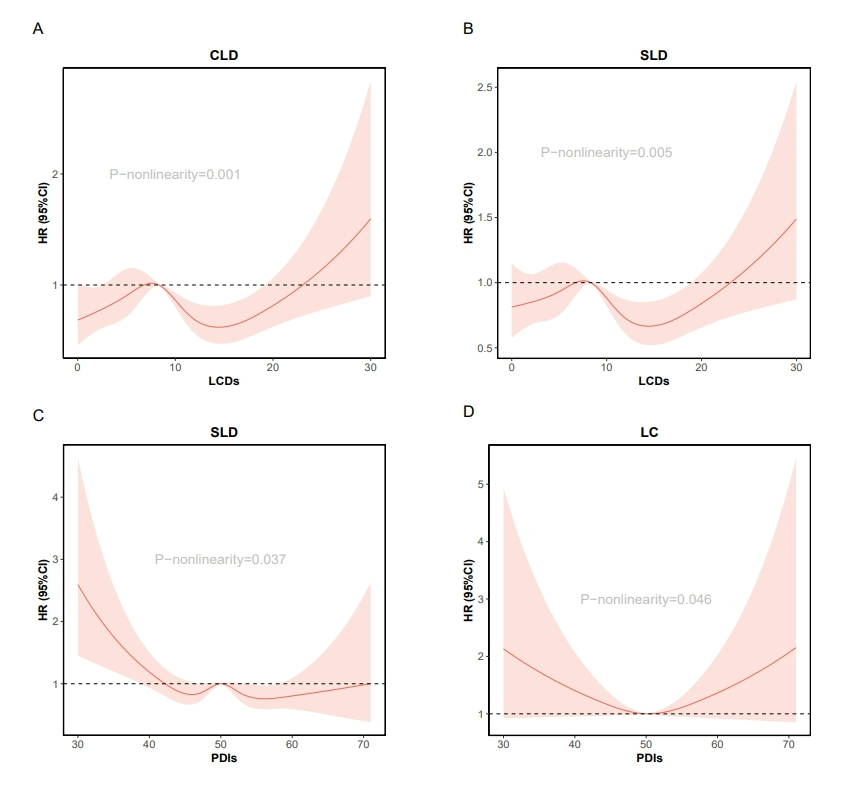
Figure S2. The nonlinear associations between the dietary pattern scores and risk of CLD, SLD and LC in the UK Biobank cohort.**

HR, hazard ratio; CI, confidence interval; CLD, chronic liver disease; SLD, severe liver disease; LC, liver cancer; LCD, Low-carbohydrate Diet; PDI, Plant-based Diet Index.

**Table S1. Detailed descriptions of dietary patterns.**

| **Patterns** | **Items** | **Score range** | **Components** |
| --- | --- | --- | --- |
| Alternate Mediterranean Diet (aMED) | 9 | 0~9 | Participants were awarded one point for meeting the following criteria: (1) greater intakes of fruits, vegetables, whole grains, nuts, legumes, fish and seafood, and ratio of monounsaturated to saturated fatty acids; (2) lower intakes of red and processed meats; and (3) moderate intake of alcohol (5-25g/day). |
| Planetary Health Diet (PHD) | 13 | 0~13 | The score included 13 components, each scored as 0 (unhealthy) or 1 (healthy) according to whether the participant’s intake met each component’s pre-specified criterion: rice and whole grain wheat (0-232 g),ubers and starchy vegetables (0-100 g), vegetables (200-600 g), fruit (100-300 g), dairy (0-500 g), red meat, unprocessed (0-28 g), poultry (0-58 g), eggs (0-25 g), fish (0-25 g), legumes (0-100 g), nuts (0-100 g), sweets (0-31 g), and healthy added fats, unsaturated to saturated fat ratio (>1.8). |
| Sulfur microbial diet (SMD) | 8 | \ | Participants were scored by summing the product of beta-coefficients and the intake of corresponding food groups. There were eight food groups, including foods with positive associations (0.64*processed meats, 0.31*liquor, and 0.38*low-calorie drinks) and those with negative associations (-0.54*beer, -0.21*fruit juice, -0.64*legumes, -0.30*other vegetables, and -0.21*sweets & desserts). |
| Low-carbohydrate diet (LCDs) | 3 | 0~30 | The energy proportions of carbohydrate, protein, and fat were divided into 11 groups, with each groups having an equal number of participants. Higher points assigned to (1) greater intakes of protein (animal protein for animal-based LCD, plant protein for plant-based LCD), and fat (animal fat for animal-based LCD, plant fat for plant-based LCD); and (2) lower intakes of carbohydrate. |
| (LCD, animal-based LCD, plant- based LCD) |  |  |  |
| Plant-based Diet Index (PDIs) | 18 | 18~90 | The index comprised 18 food groups, which were classified into three broad categories: (1) healthy plant foods, which included fruits, vegetables, whole grains, nuts, legumes, tea and coffee, and vegetable oils; (2) less healthy plant foods, which comprised potatoes, refined grains, sugar-sweetened beverages, fruit juices, sweets and desserts., and (3) animal foods, which included meats, eggs, fish and seafood, miscellaneous animal-based foods, dairy, and animal fat. For PDI, both healthy and less healthy plant foods received positive scores, while animal foods received negative scores. For hPDI, healthy plant foods were assigned positive scores, while both less healthy plant foods and animal foods received negative scores. Conversely, less healthy plant foods received positive scores for uPDI, while both healthy plant foods and animal foods were assigned negative scores. |
| (PDI, healthful PDI, unhealthful PDI)) |  |  |  |

**Table S2. Criteria for endpoint events of different liver diseases.**

| **ICD-10** | **Description** |
| --- | --- |
| **Chronic liver disease (CLD)** | |
| I85.0 | Esophageal varices, bleeding |
| I85.9 | Esophageal varices, not bleeding |
| K70.2 | Alcoholic fibrosis and sclerosis of the liver |
| K70.3 | Alcoholic liver cirrhosis |
| K70.4 | Alcoholic liver failure |
| K74.0 | Hepatic fibrosis |
| K74.1 | Hepatic sclerosis |
| K74.2 | Hepatic fibrosis with hepatic sclerosis |
| K74.6 | Liver cirrhosis, other and unspecified |
| K76.6 | Portal hypertension |
| **Severe liver disease (SLD)** | |
| C22.0 | Liver cell carcinoma |
| I85.0 | Esophageal varices, bleeding |
| I85.9 | Esophageal varices, not bleeding |
| K70.3 | Alcoholic liver cirrhosis |
| K70.4 | Alcoholic liver failure |
| K72.1 | Liver failure, chronic |
| K72.9 | Liver failure, unspecified |
| K74.1 | Hepatic sclerosis |
| K74.2 | Hepatic fibrosis with hepatic sclerosis |
| K74.6 | Liver cirrhosis, other and unspecified |
| K76.6 | Portal hypertension |
| K76.7 | Hepatorenal syndrome |
| Z94.4 | Liver transplant status |
| **Liver cancer (LC)** | |
| C22 | Malignant neoplasm of liver and intrahepatic bile ducts |
| C22.0 | Liver cell carcinoma |
| C22.1 | Intrahepatic bile duct carcinoma |
| C22.2 | Hepatoblastoma |
| C22.3 | Angiosarcoma of liver |
| C22.4 | Other sarcomas of liver |
| C22.7 | Other specified carcinomas of liver |
| C22.9 | Liver, unspecified |
| **Hepatocellular carcinoma (HCC)** | |
| C22.0 | Liver cell carcinoma |
| **Intrahepatic cholangiocarcinoma（ICC）** | |
| C22.1 | Intrahepatic bile duct carcinoma |

**Table S3. ICD-9 & ICD-10 codes used to define baseline liver disease.**

| **Diagnosis** | **ICD-9** | **ICD-10** |
| --- | --- | --- |
| Viral hepatitis | 070,573.1 | B15-B19 |
| Hepatocellular carcinoma | 155 | C22 |
| Disorders of copper metabolism | 275.1 | E83.0 |
| Disorders of iron metabolism | 275.0 | E83.1 |
| Esophageal varices, bleeding | 456.0 | I85.0 |
| Esophageal varices, not bleeding | 456.1 | I85.9 |
| Alcoholic liver disease | 571.0,571.1,571.2,571.3 | K70 |
| Toxic liver disease |  | K71 |
| Liver failure, chronic |  | K72.1 |
| Liver failure, unspecified |  | K72.9 |
| Chronic hepatitis, not elsewhere classified | 571.4,571.9 | K73 |
| Liver fibrosis and cirrhosis | 571.5,571.6 | K74 |
| Nonspecific reactive hepatitis |  | K75.2 |
| Granulomatous hepatitis, not elsewhere classified |  | K75.3 |
| Autoimmune hepatitis |  | K75.4 |
| Other specified inflammatory liver diseases |  | K75.8 |
| Inflammatory liver disease, unspecified | 573.3 | K75.9 |
| Fatty liver, not elsewhere classified | 571.8 | K76.0 |
| Portal hypertension | 572.3 | K76.6 |
| Hepatorenal syndrome | 572.4 | K76.7 |
| Other specified diseases of liver | 573.8 | K76.8 |
| Liver disease, unspecified | 573.9 | K76.9 |
| Ascites | 789.5 | R18 |
| Liver transplant status | V42.7 | Z94.4 |

**Table S4. The associations of MAFLD with the incidence and mortality of liver diseases in the UK Biobank cohort.**

| **Outcome** | **Non-MAFLD** | **MAFLD** | **Model1** | | **Model2** | |
| --- | --- | --- | --- | --- | --- | --- |
|  | **Cases**  **(Person-years)** | **Cases**  **(Person-years)** | **HR**  **(95% CI)** | ***P*** | **HR**  **(95% CI)** | ***P*** |
| CLD | 568  (3867575) | 1651  (2381247) | 4.05  [3.67, 4.47] | <0.001 | 3.48  [3.15, 3.84] | <0.001 |
| SLD | 767  (3866245) | 1862  (2380105) | 3.31  [3.03, 3.61] | <0.001 | 2.87  [2.63, 3.14] | <0.001 |
| LC | 335  (3868738) | 514  (2387894) | 2.09  [1.81, 2.41] | <0.001 | 1.93  [1.67, 2.23] | <0.001 |
| HCC | 88  (3866938) | 250  (2385933) | 3.40  [2.65, 4.37] | <0.001 | 3.01  [2.34, 3.88] | <0.001 |
| ICC | 187  (3867618) | 184  (2385294) | 1.47  [1.19, 1.82] | <0.001 | 1.42  [1.14, 1.76] | 0.002 |
| LRD | 317  (3588682) | 654  (2183988) | 2.80  [2.44, 3.22] | <0.001 | 2.45  [2.13, 2.82] | <0.001 |

The Cox regression model derived HRs, 95% CIs, and *P*-values by using participants without MAFLD as the reference group. Model 1 was adjusted for age at recruitment and sex, and Model 2 was further adjusted for race, TDI, smoking and drinking status, educational attainment, physical activity, and family history of cancer.

MAFLD, metabolic dysfunction-associated fatty liver disease; HR, hazard ratio; CI, confidence interval; CLD, chronic liver disease; SLD, severe liver disease; LC, liver cancer; HCC, hepatocellular carcinoma; ICC, intrahepatic cholangiocarcinoma; LRD, liver-related death.

**Table S5. Subgroup analyses for associations of MALFD with the incidence and mortality of liver diseases.**

|  | **CLD** | **SLD** | **LC** | **HCC** | **ICC** | **LRD** |
| --- | --- | --- | --- | --- | --- | --- |
|  | HR (95% CI) | HR (95% CI) | HR (95% CI) | HR (95% CI) | HR (95% CI) | HR (95% CI) |
| **Sex** |  |  |  |  |  |  |
| Female | 4.50 [3.86, 5.23] | 3.49 [3.05, 4.00] | 1.57 [1.26, 1.97] | 2.03 [1.32, 3.11] | 1.36 [1.00, 1.85] | 2.68 [2.16, 3.33] |
| Male | 2.90 [2.54, 3.30] | 2.53 [2.25, 2.83] | 2.35 [1.92, 2.89] | 3.86 [2.73, 5.45] | 1.57 [1.15, 2.14] | 2.32 [1.92, 2.79] |
| MAFLD * Male | 0.66 [0.54, 0.80] | 0.74 [0.62, 0.88] | 1.61 [1.19, 2.17] | 2.07 [1.21, 3.56] | 1.20 [0.78, 1.85] | 0.88 [0.67, 1.17] |
| *P*-interaction | <0.001 | 0.001 | 0.002 | 0.008 | 0.404 | 0.384 |
| **Age at recruitment** |  |  |  |  |  |  |
| <60 | 3.40 [2.97, 3.90] | 2.86 [2.52, 3.24] | 1.89 [1.48, 2.43] | 2.43 [1.57, 3.77] | 1.49 [1.04, 2.15] | 2.21 [1.78, 2.74] |
| ≥60 | 3.69 [3.18, 4.27] | 2.99 [2.64, 3.38] | 2.03 [1.69, 2.43] | 3.44 [2.51, 4.71] | 1.43 [1.09, 1.87] | 2.75 [2.28, 3.32] |
| MAFLD * ≥60 | 1.02 [0.84, 1.24] | 1.00 [0.85, 1.19] | 1.15 [0.86, 1.54] | 1.44 [0.86, 2.40] | 1.06 [0.69, 1.61] | 1.14 [0.87, 1.50] |
| *P*-interaction | 0.843 | 0.985 | 0.337 | 0.164 | 0.805 | 0.332 |
| **Education** |  |  |  |  |  |  |
| College and above | 3.84 [3.12, 4.73] | 3.25 [2.71, 3.89] | 2.02 [1.52, 2.70] | 3.16 [1.89, 5.29] | 1.44 [0.95, 2.20] | 2.96 [2.21, 3.97] |
| Below college | 3.42 [3.05, 3.83] | 2.81 [2.54, 3.11] | 1.95 [1.64, 2.31] | 3.02 [2.25, 4.04] | 1.44 [1.12, 1.85] | 2.37 [2.01, 2.78] |
| MAFLD * Below college | 0.86 [0.69, 1.08] | 0.87 [0.71, 1.06] | 1.02 [0.74, 1.40] | 1.01 [0.58, 1.79] | 1.04 [0.65, 1.66] | 0.86 [0.63, 1.18] |
| *P*-interaction | 0.199 | 0.166 | 0.905 | 0.960 | 0.867 | 0.339 |
| **Alcohol consumption** | |  |  |  |  |  |
| None to moderate | 3.43 [3.04, 3.88] | 2.89 [2.60, 3.22] | 1.85 [1.56, 2.19] | 2.62 [1.95, 3.50] | 1.43 [1.11, 1.83] | 2.35 [1.97, 2.80] |
| Excess | 3.65 [3.06, 4.35] | 2.90 [2.48, 3.39] | 2.20 [1.65, 2.94] | 4.52 [2.58, 7.90] | 1.48 [0.96, 2.28] | 2.65 [2.08, 3.38] |
| MAFLD * Excess | 1.20 [0.98 1.47] | 1.17 [0,97, 1.39] | 1.45 [1.06, 1.99] | 2.47 [1.34, 4.55] | 1.06 [0.67, 1.68] | 1.34 [1.01, 1.77] |
| *P*-interaction | 0.074 | 0.095 | 0.020 | 0.004 | 0.802 | 0.045 |
| **Smoking status** |  |  |  |  |  |  |
| Never smoked | 3.97 [3.41, 4.62] | 3.18 [2.78, 3.64] | 1.83 [1.46, 2.28] | 2.94 [2.00, 4.33] | 1.44 [1.04, 1.99] | 2.89 [2.31, 3.62] |
| Previous or current smokers | 3.21 [2.82, 3.67] | 2.73 [2.42, 3.07] | 2.10 [1.73, 2.55] | 3.15 [2.24, 4.43] | 1.47 [1.10, 1.96] | 2.27 [1.89, 2.72] |
| MAFLD * Previous or current smokers | 0.82 [0.68, 1.00] | 0.87 [0.74, 1.04] | 1.21 [0.91, 1.60] | 1.25 [0.76, 2.04] | 1.00 [0.66, 1.51] | 0.79 [0.60, 1.04] |
| *P*-interaction | 0.046 | 0.118 | 0.193 | 0.386 | 0.983 | 0.090 |

The Cox regression model derived HRs, 95% CIs, and *P-*values using a fully adjusted model that accounted for age, sex, race, TDI, educational attainment, smoking and drinking status, physical activity, and family history of cancer.

MAFLD, metabolic dysfunction-associated fatty liver disease; HR, hazard ratio; CI, confidence interval; CLD, chronic liver disease; SLD, severe liver disease; LC, liver cancer; HCC, hepatocellular carcinoma; ICC, intrahepatic cholangiocarcinoma; LRD, liver-related death.

**Table S6. The associations of the scores of dietary patterns with the incidence of chronic liver disease and severe liver disease in the UK Biobank cohort.**

|  | Median  (Score range) | **Chronic liver disease (CLD)** | | | | | | | | **Severe liver disease (SLD)** | | | | | | | |
| --- | --- | --- | --- | --- | --- | --- | --- | --- | --- | --- | --- | --- | --- | --- | --- | --- | --- |
|  |  | Cases | Person-years | Model 1 HR (95% CI) | Model 1 *P* | Model 1 *P*_adjusted_ | Model 2 HR (95% CI) | Model 2 *P* | Model 2 *P*_adjusted_ | Cases | Person-years | Model1 HR (95% CI) | Model 1 *P* | Model 1 *P*_adjusted_ | Model 2 HR  (95% CI) | Model 2 *P* | Model 2 *P*_adjusted_ |
|  | **aMED** |  |  |  |  |  |  |  |  |  |  |  |  |  |  |  |  |
| Q1 | 2  [0, 2] | 180 | 413198 | Ref. |  |  | Ref. |  |  | 204 | 413078 | Ref. |  |  | Ref. |  |  |
| Q2 | 3  [2, 3] | 144 | 512994 | 0.63  [0.51, 0.79] | <0.001 | 1.54E-04 | 0.70  [0.56, 0.88] | 0.002 | 0.008 | 183 | 512748 | 0.70  [0.57, 0.86] | <0.001 | 0.002 | 0.77  [0.63, 0.94] | 0.011 | 0.036 |
| Q3 | 4  [3, 4] | 126 | 539033 | 0.52  [0.42, 0.66] | <0.001 | 4.55E-07 | 0.62  [0.50, 0.79] | <0.001 | 0.002 | 146 | 538923 | 0.53  [0.43, 0.65] | <0.001 | 9.00E-08 | 0.61  [0.50, 0.76] | <0.001 | 3.11E-04 |
| Q4 | 5  [4, 5] | 74 | 361055 | 0.46  [0.35, 0.60] | <0.001 | 4.55E-07 | 0.59  [0.45, 0.77] | <0.001 | 0.003 | 108 | 360869 | 0.58  [0.46, 0.73] | <0.001 | 1.97E-05 | 0.72  [0.56, 0.91] | 0.006 | 0.021 |
| Q5 | 6  [5, 9] | 38 | 220788 | 0.37  [0.26, 0.53] | <0.001 | 4.55E-07 | 0.53  [0.37, 0.76] | 0.001 | 0.003 | 45 | 220768 | 0.38  [0.28, 0.53] | <0.001 | 9.00E-08 | 0.52  [0.37, 0.72] | <0.001 | 0.001 |
| P for trend |  |  |  | 0.78  [0.72, 0.83] | <0.001 | 8.87E-12 | 0.84  [0.79, 0.91] | <0.001 | 3.02E-05 |  |  | 0.80  [0.75, 0.85] | <0.001 | 3.85E-11 | 0.86  [0.81, 0.92] | <0.001 | 5.27E-05 |
| per 1 SD |  |  |  | 0.75  [0.69, 0.82] | <0.001 | 7.67E-10 | 0.84  [0.77, 0.92] | <0.001 | 3.81E-04 |  |  | 0.78  [0.72, 0.84] | <0.001 | 1.78E-09 | 0.85  [0.79, 0.92] | <0.001 | 4.09E-04 |
|  | **PHD** |  |  |  |  |  |  |  |  |  |  |  |  |  |  |  |  |
| Q1 | 2  [0, 2.5] | 199 | 488241 | Ref. |  |  | Ref. |  |  | 232 | 488037 | Ref. |  |  | Ref. |  |  |
| Q2 | 3  [2.5, 3] | 117 | 482939 | 0.60  [0.48, 0.75] | <0.001 | 5.71E-05 | 0.66  [0.52, 0.83] | <0.001 | 0.003 | 149 | 482751 | 0.65  [0.53, 0.80] | <0.001 | 1.52E-04 | 0.70  [0.57, 0.86] | 0.001 | 0.004 |
| Q3 | 3.5  [3, 3.67] | 70 | 297236 | 0.59  [0.45, 0.78] | <0.001 | 5.37E-04 | 0.72  [0.55, 0.94] | 0.018 | 0.054 | 80 | 297187 | 0.57  [0.44, 0.74] | <0.001 | 8.01E-05 | 0.67  [0.52, 0.87] | 0.002 | 0.010 |
| Q4 | 4  [3.67, 4.25] | 102 | 384356 | 0.68  [0.53, 0.86] | 0.001 | 3.45E-03 | 0.80  [0.62, 1.02] | 0.066 | 0.162 | 126 | 384238 | 0.71  [0.57, 0.88] | 0.002 | 4.54E-03 | 0.81  [0.65, 1.01] | 0.063 | 0.176 |
| Q5 | 5  [4.25, 9] | 74 | 394296 | 0.49  [0.38, 0.64] | <0.001 | 1.68E-06 | 0.62  [0.47, 0.81] | <0.001 | 0.003 | 99 | 394173 | 0.55  [0.44, 0.70] | <0.001 | 1.06E-05 | 0.67  [0.53, 0.85] | 0.001 | 0.004 |
| P for trend |  |  |  | 0.80  [0.74, 0.87] | <0.001 | 4.29E-07 | 0.87  [0.80, 0.94] | 0.001 | 0.002 |  |  | 0.83  [0.77, 0.89] | <0.001 | 1.86E-06 | 0.88  [0.82, 0.95] | 0.001 | 0.003 |
| per 1 SD |  |  |  | 0.78  [0.72, 0.86] | <0.001 | 9.09E-08 | 0.86  [0.79, 0.93] | <0.001 | 0.001 |  |  | 0.81  [0.75, 0.88] | <0.001 | 5.42E-07 | 0.88  [0.81, 0.95] | 0.001 | 0.002 |
|  | **SMD** |  |  |  |  |  |  |  |  |  |  |  |  |  |  |  |  |
| Q1 | -2.56  [-12.94, -2.03] | 107 | 410201 | Ref. |  |  | Ref. |  |  | 134 | 410063 | Ref. |  |  | Ref. |  |  |
| Q2 | -1.7  [-2.03, -1.44] | 91 | 409583 | 0.88  [0.66, 1.16] | 0.363 | 0.408 | 0.94  [0.71, 1.25] | 0.678 | 0.787 | 118 | 409444 | 0.91  [0.71, 1.16] | 0.435 | 0.496 | 0.96  [0.75, 1.23] | 0.760 | 0.944 |
| Q3 | -1.2  [-1.44, -0.97] | 99 | 409985 | 0.97  [0.74, 1.28] | 0.832 | 0.856 | 1.03  [0.78, 1.36] | 0.837 | 0.837 | 118 | 409882 | 0.92  [0.72, 1.18] | 0.524 | 0.571 | 0.97  [0.75, 1.35] | 0.803 | 0.945 |
| Q4 | -0.73  [-0.97, -0.44] | 110 | 408921 | 1.08  [0.83, 1.42] | 0.549 | 0.599 | 1.08  [0.82, 1.41] | 0.596 | 0.716 | 131 | 408818 | 1.04  [0.81, 1.32] | 0.776 | 0.799 | 1.03  [0.80, 1.31] | 0.843 | 0.945 |
| Q5 | 0  [-0.44, 7,17] | 155 | 408379 | 1.52  [1.19, 1.95] | 0.001 | 0.002 | 1.24  [0.96, 1.61] | 0.099 | 0.211 | 185 | 408181 | 1.47  [1.18, 1.84] | 0.001 | 0.002 | 1.23  [0.98, 1.56] | 0.077 | 0.197 |
| P for trend |  |  |  | 1.20  [1.09, 1.32] | <0.001 | 3.57E-04 | 1.10  [1.00, 1.21] | 0.057 | 0.073 |  |  | 1.17  [1.07, 1.28] | <0.001 | 5.49E-04 | 1.09  [1.00, 1.19] | 0.059 | 0.088 |
| per 1 SD |  |  |  | 1.22  [1.13, 1.33] | <0.001 | 3.46E-06 | 1.12  [1.03, 1.21] | 0.005 | 0.008 |  |  | 1.18  [1.10, 1.27] | <0.001 | 2.10E-05 | 1.10  [1.02, 1.18] | 0.012 | 0.017 |
|  | **LCD** |  |  |  |  |  |  |  |  |  |  |  |  |  |  |  |  |
| Q1 | 2.33  [0, 4] | 99 | 440301 | Ref. |  |  | Ref. |  |  | 132 | 440097 | Ref. |  |  | Ref. |  |  |
| Q2 | 6  [4, 7] | 139 | 431488 | 1.44  [1.11, 1.86] | 0.006 | 0.012 | 1.38  [1.07, 1.79] | 0.014 | 0.046 | 163 | 431387 | 1.26  [1.00, 1.58] | 0.050 | 0.086 | 1.22  [0.97, 1.53] | 0.094 | 0.214 |
| Q3 | 9  [7, 10] | 140 | 435399 | 1.47  [1.14, 1.90] | 0.003 | 0.007 | 1.30  [1.01, 1.69] | 0.044 | 0.123 | 166 | 435262 | 1.30  [1.04, 1.64] | 0.023 | 0.047 | 1.18  [0.93, 1.48] | 0.167 | 0.333 |
| Q4 | 12  [10, 13.5] | 77 | 344096 | 1.04  [0.77, 1.39] | 0.820 | 0.856 | 0.89  [0.66, 1.20] | 0.441 | 0.610 | 96 | 343990 | 0.96  [0.74, 1.25] | 0.777 | 0.799 | 0.84  [0.65, 1.10] | 0.210 | 0.394 |
| Q5 | 17  [13.5, 30] | 107 | 395784 | 1.29  [0.98, 1.70] | 0.065 | 0.086 | 1.05  [0.80, 1.38] | 0.733 | 0.825 | 129 | 395650 | 1.17  [0.91, 1.49] | 0.216 | 0.287 | 0.96  [0.75, 1.23] | 0.758 | 0.944 |
| P for trend |  |  |  | 1.01  [0.99, 1.02] | 0.438 | 0.438 | 0.99  [0.98, 1.01] | 0.323 | 0.363 |  |  | 1.00  [0.99, 1.02] | 0.717 | 0.717 | 0.99  [0.97, 1.00] | 0.160 | 0.204 |
| per 1 SD |  |  |  | 1.06  [0.97, 1.15] | 0.188 | 0.188 | 0.98  [0.90, 1.06] | 0.613 | 0.690 |  |  | 1.03  [0.96, 1.11] | 0.387 | 0.387 | 0.96  [0.89, 1.04] | 0.338 | 0.381 |
|  | **aLCD** |  |  |  |  |  |  |  |  |  |  |  |  |  |  |  |  |
| Q1 | 1  [0, 2.5] | 89 | 425256 | Ref. |  |  | Ref. |  |  | 123 | 425077 | Ref. |  |  | Ref. |  |  |
| Q2 | 4  [2.5, 5] | 118 | 399572 | 1.39  [1.05, 1.83] | 0.020 | 0.037 | 1.29  [0.98, 1.71] | 0.067 | 0.162 | 135 | 399481 | 1.14  [0.90, 1.46] | 0.280 | 0.348 | 1.08  [0.84, 1.38] | 0.552 | 0.736 |
| Q3 | 7  [5, 8] | 112 | 409567 | 1.29  [0.98, 1.71] | 0.071 | 0.092 | 1.11  [0.84, 1.47] | 0.460 | 0.613 | 140 | 409415 | 1.16  [0.91, 1.48] | 0.223 | 0.287 | 1.02  [0.80, 1.31] | 0.861 | 0.94 |
| Q4 | 10  [8, 12] | 119 | 413458 | 1.38  [1.05, 1.81] | 0.022 | 0.039 | 1.08  [0.82, 1.43] | 0.585 | 0.716 | 136 | 413365 | 1.14  [0.89, 1.45] | 0.306 | 0.356 | 0.92  [0.72, 1.18] | 0.511 | 0.708 |
| Q5 | 16  [12, 30] | 124 | 399215 | 1.52  [1.16, 1.99] | 0.003 | 0.006 | 1.10  [0.83, 1.45] | 0.505 | 0.649 | 152 | 399048 | 1.34  [1.06, 1.70] | 0.016 | 0.033 | 1.01  [0.80, 1.29] | 0.916 | 0.945 |
| P for trend |  |  |  | 1.02  [1.01, 1.04] | 0.010 | 0.011 | 1.00  [0.98, 1.02] | 0.897 | 0.897 |  |  | 1.02  [1.00, 1.03] | 0.025 | 0.028 | 1.00  [0.98, 1.01] | 0.726 | 0.726 |
| per 1 SD |  |  |  | 1.13  [1.04 1.22] | 0.003 | 0.003 | 1.01  [0.93, 1.09] | 0.847 | 0.847 |  |  | 1.10  [1.02, 1.18] | 0.013 | 0.015 | 0.99  [0.92, 1.07] | 0.857 | 0.857 |
|  | **pLCD** |  |  |  |  |  |  |  |  |  |  |  |  |  |  |  |  |
| Q1 | 11  [0, 12.5] | 89 | 425256 | Ref. |  |  | Ref. |  |  | 123 | 425077 | Ref. |  |  | Ref. |  |  |
| Q2 | 14  [12.5, 15] | 118 | 399572 | 0.78  [0.61, 1.00] | 0.051 | 0.073 | 0.83  [0.65, 1.06] | 0.132 | 0.264 | 135 | 399481 | 0.89  [0.71, 1.11] | 0.290 | 0.348 | 0.93  [0.74, 1.15] | 0.497 | 0.708 |
| Q3 | 16.5  [15, 17.5] | 112 | 409567 | 0.78  [0.61, 1.00] | 0.051 | 0.073 | 0.85  [0.67, 1.10] | 0.219 | 0.343 | 140 | 409415 | 0.78  [0.62, 0.98] | 0.036 | 0.065 | 0.84  [0.67, 1.06] | 0.139 | 0.294 |
| Q4 | 19  [17.5, 20] | 119 | 413458 | 0.79  [0.62, 1.00] | 0.050 | 0.073 | 0.88  [0.69, 1.12] | 0.298 | 0.447 | 136 | 413365 | 0.85  [0.68, 1.06] | 0.154 | 0.231 | 0.93  [0.74, 1.16] | 0.504 | 0.708 |
| Q5 | 22  [20, 30] | 124 | 399215 | 0.61  [0.46, 0.80] | <0.001 | 0.001 | 0.65  [0.49, 0.86] | 0.003 | 0.011 | 152 | 399048 | 0.62  [0.48, 0.80] | <0.001 | 8.06E-04 | 0.65  [0.50, 0.84] | 0.001 | 0.004 |
| P for trend |  |  |  | 0.96  [0.94, 0.99] | 0.001 | 0.001 | 0.97  [0.95, 0.99] | 0.012 | 0.021 |  |  | 0.97  [0.95, 0.98] | 0.001 | 7.28E-04 | 0.97  [0.95, 0.99] | 0.004 | 0.007 |
| per 1 SD |  |  |  | 0.86  [0.79, 0.93] | <0.001 | 3.01E-04 | 0.88  [0.81, 0.96] | 0.003 | 0.006 |  |  | 0.86  [0.80, 0.93] | <0.001 | 1.13E-04 | 0.88  [0.81, 0.95] | 0.001 | 0.002 |
|  | **PDI** |  |  |  |  |  |  |  |  |  |  |  |  |  |  |  |  |
| Q1 | 44  [26, 46] | 163 | 438898 | Ref. |  |  | Ref. |  |  | 190 | 438766 | Ref. |  |  | Ref. |  |  |
| Q2 | 48  [46, 49] | 135 | 422698 | 0.89  [0.71, 1.11] | 0.298 | 0.347 | 1.03  [0.82, 1.29] | 0.817 | 0.837 | 154 | 422560 | 0.86  [0.70, 1.06] | 0.167 | 0.250 | 0.98  [0.79, 1.22] | 0.869 | 0.945 |
| Q3 | 50.5  [49, 51.5] | 99 | 368510 | 0.76  [0.59, 0.98] | 0.033 | 0.054 | 0.97  [0.75, 1.25] | 0.819 | 0.837 | 124 | 368369 | 0.81  [0.65, 1.02] | 0.069 | 0.113 | 1.00  [0.80, 1.26] | 0.970 | 0.970 |
| Q4 | 53  [51.5, 54.5] | 89 | 411617 | 0.63  [0.48, 0.81] | <0.001 | 0.001 | 0.84  [0.65, 1.10] | 0.201 | 0.328 | 116 | 411465 | 0.69  [0.55, 0.87] | 0.002 | 0.005 | 0.89  [0.71, 1.13] | 0.350 | 0.573 |
| Q5 | 57  [54.5, 77] | 76 | 405346 | 0.55  [0.42, 0.72] | <0.001 | 8.46E-05 | 0.79  [0.59, 1.05] | 0.100 | 0.211 | 102 | 405226 | 0.63  [0.49, 0.80] | <0.001 | 5.18E-04 | 0.85  [0.67, 1.10] | 0.219 | 0.394 |
| P for trend |  |  |  | 0.95  [0.93, 0.97] | <0.001 | 1.27E-06 | 0.98  [0.96, 1.00] | 0.055 | 0.073 |  |  | 0.96  [0.95, 0.98] | <0.001 | 3.56E-05 | 0.99  [0.97, 1.01] | 0.181 | 0.204 |
| per 1 SD |  |  |  | 0.77  [0.71, 0.84] | <0.001 | 2.71E-09 | 0.88  [0.81, 0.96] | 0.004 | 0.006 |  |  | 0.81  [0.75, 0.87] | <0.001 | 1.22E-07 | 0.91  [0.84, 0.98] | 0.016 | 0.020 |
|  | **hPDI** |  |  |  |  |  |  |  |  |  |  |  |  |  |  |  |  |
| Q1 | 50  [31, 52] | 170 | 435473 | Ref. |  |  | Ref. |  |  | 204 | 435300 | Ref. |  |  | Ref. |  |  |
| Q2 | 54  [52, 55.33] | 122 | 388498 | 0.81  [0.64, 1.02] | 0.079 | 0.098 | 0.86  [0.68, 1.08] | 0.194 | 0.328 | 145 | 388391 | 0.79  [0.64, 0.98] | 0.031 | 0.060 | 0.83  [0.67, 1.03] | 0.095 | 0.214 |
| Q3 | 57  [55.33, 58] | 121 | 419344 | 0.76  [0.60, 0.96] | 0.024 | 0.040 | 0.84  [0.66, 1.06] | 0.145 | 0.270 | 144 | 419210 | 0.74  [0.60, 0.92] | 0.006 | 0.013 | 0.81  [0.65, 1.01] | 0.056 | 0.169 |
| Q4 | 60  [58, 61.5] | 76 | 396062 | 0.53  [0.40, 0.69] | <0.001 | 2.60E-05 | 0.60  [0.46, 0.80] | <0.001 | 0.003 | 98 | 395923 | 0.55  [0.43, 0.70] | <0.001 | 1.10E-05 | 0.62  [0.48, 0.80] | <0.001 | 0.002 |
| Q5 | 64  [61.5, 83] | 73 | 407691 | 0.52  [0.39, 0.69] | <0.001 | 2.60E-05 | 0.63  [0.47, 0.84] | 0.002 | 0.008 | 95 | 407563 | 0.54  [0.43, 0.70] | <0.001 | 1.10E-05 | 0.65  [0.50, 0.78] | 0.001 | 0.004 |
| P for trend |  |  |  | 0.95  [0.93, 0.97] | <0.001 | 1.34E-07 | 0.96  [0.95, 0.98] | <0.001 | 4.46E-04 |  |  | 0.95  [0.94, 0.97] | <0.001 | 5.13E-08 | 0.97  [0.95, 0.84] | <0.001 | 2.69E-04 |
| per 1 SD |  |  |  | 0.77  [0.71, 0.84] | <0.001 | 1.13E-08 | 0.82  [0.75, 0.90] | <0.001 | 2.33E-04 |  |  | 0.78  [0.72, 0.85] | <0.001 | 3.87E-09 | 0.83  [0.77, 0.98] | <0.001 | 1.02E-04 |
|  | **uPDI** |  |  |  |  |  |  |  |  |  |  |  |  |  |  |  |  |
| Q1 | 50  [28, 52] | 98 | 433372 | Ref. |  |  | Ref. |  |  | 123 | 433220 | Ref. |  |  | Ref. |  |  |
| Q2 | 54  [52, 55.5] | 90 | 403687 | 0.99  [0.74, 1.31] | 0.926 | 0.926 | 0.97  [0.73, 1.29] | 0.835 | 0.837 | 114 | 403576 | 1.00  [0.78, 1.29] | 0.991 | 0.991 | 0.99  [0.76, 1.27] | 0.919 | 0.945 |
| Q3 | 57  [55.5, 58] | 116 | 397832 | 1.30  [0.99, 1.70] | 0.059 | 0.081 | 1.22  [0.93, 1.60] | 0.150 | 0.270 | 134 | 397711 | 1.21  [0.95, 1.54] | 0.129 | 0.202 | 1.15  [0.90, 1.47] | 0.269 | 0.461 |
| Q4 | 60  [58, 61.5] | 112 | 407933 | 1.23  [0.94, 1.62] | 0.133 | 0.160 | 1.13  [0.86, 1.48] | 0.399 | 0.574 | 133 | 407838 | 1.19  [0.93, 1.52] | 0.170 | 0.235 | 1.10  [0.86, 1.41] | 0.441 | 0.690 |
| Q5 | 64  [61.5, 80] | 146 | 404245 | 1.69  [1.30, 2.19] | <0.001 | 2.55E-04 | 1.42  [1.09, 1.85] | 0.009 | 0.033 | 182 | 404041 | 1.74  [1.38, 2.19] | <0.001 | 1.65E-05 | 1.50  [1.19, 1.90] | 0.001 | 0.004 |
| P for trend |  |  |  | 1.04  [1.02, 1.06] | <0.001 | 2.86E-05 | 1.03  [1.01, 1.04] | 0.004 | 0.010 |  |  | 1.04  [1.02, 1.06] | <0.001 | 1.96E-06 | 1.03  [1.01, 1.05] | <0.001 | 0.001 |
| per 1 SD |  |  |  | 1.20  [1.10, 1.30] | <0.001 | 4.88E-05 | 1.12  [1.03, 1.22] | 0.008 | 0.010 |  |  | 1.21  [1.12, 1.30] | <0.001 | 3.64E-06 | 1.14  [1.06, 1.23] | 0.001 | 0.002 |

The nine dietary pattern scores were divided into quintiles and treated as categorical variables. Model 1 was adjusted for age at first completion of the Oxford WebQ and sex, and Model 2 was further adjusted for race, educational attainment, TDI, family history of cancer, smoking status, average alcohol intake (except aMED & SMD), MET, average energy intake, BMI, WHR and T2DM. The *P*_adjusted_ was determined using a multiple comparison test based on the false discovery rate (FDR).

HR, hazard ratio; CI, confidence interval; aMED, alternate Mediterranean diet; PHD, Planetary Health Diet; SMD, sulfur microbial diet; LCD, Low-carbohydrate Diet; aLCD, animal based Low-carbohydrate Diet; pLCD, plant based Low-carbohydrate Diet; PDI, Plant-based Diet Index; hPDI, healthful Plant-based Diet Index; uPDI, unhealthful Plant-based Diet Index.

**Table S7. The associations of the scores of dietary patterns with the incidence of liver cancer and mortality of liver diseases in the UK Biobank cohort.**

|  | Median  (Score range) | **Liver-related death(LRD)** | | | | | | | | **Liver cancer (LC)** | | | | | | | |
| --- | --- | --- | --- | --- | --- | --- | --- | --- | --- | --- | --- | --- | --- | --- | --- | --- | --- |
|  |  | Cases | Person-years | Model 1 HR (95% CI) | Model 1 *P* | Model 1 *P*_adjusted_ | Model 2 HR (95% CI) | Model 2 *P* | Model 2 *P*_adjusted_ | Cases | Person-years | Model1 HR (95% CI) | Model 1 *P* | Model 1 *P*_adjusted_ | Model 2 HR  (95% CI) | Model 2 *P* | Model 2 *P*_adjusted_ |
|  | **aMED** |  |  |  |  |  |  |  |  |  |  |  |  |  |  |  |  |
| Q1 | 2  [0, 2] | 65 | 403484 | Ref. |  |  | Ref. |  |  | 63 | 413773 | Ref. |  |  | Ref. |  |  |
| Q2 | 3  [2, 3] | 62 | 501737 | 0.73  [0.51, 1.03] | 0.072 | 0.216 | 0.79  [0.55, 1.11] | 0.177 | 0.530 | 62 | 513435 | 0.74  [0.52, 1.05] | 0.095 | 0.311 | 0.78  [0.55, 1.11] | 0.169 | 0.592 |
| Q3 | 4  [3, 4] | 48 | 528562 | 0.52  [0.36, 0.76] | 0.001 | 0.012 | 0.60  [0.41, 0.87] | 0.008 | 0.103 | 62 | 539309 | 0.69  [0.48, 0.98] | 0.036 | 0.292 | 0.75  [0.53, 1.07] | 0.108 | 0.592 |
| Q4 | 5  [4, 5] | 44 | 354526 | 0.70  [0.48, 1.02] | 0.067 | 0.216 | 0.85  [0.58, 1.26] | 0.428 | 0.669 | 52 | 361136 | 0.83  [0.58, 1.20] | 0.333 | 0.461 | 0.95  [0.65, 1.37] | 0.771 | 0.867 |
| Q5 | 6  [5, 9] | 18 | 216591 | 0.45  [0.27, 0.76] | 0.003 | 0.031 | 0.60  [0.35, 1.03] | 0.063 | 0.339 | 18 | 220907 | 0.45  [0.26, 0.76] | 0.003 | 0.097 | 0.53  [0.31, 0.91] | 0.021 | 0.592 |
| P for trend |  |  |  | 0.84  [0.76, 0.94] | 0.001 | 0.007 | 0.91  [0.81, 1.01] | 0.069 | 0.247 |  |  | 0.89  [0.80, 0.98] | 0.016 | 0.070 | 0.92  [0.84, 1.02] | 0.125 | 0.561 |
| per 1 SD |  |  |  | 0.80  [0.71, 0.92] | 0.001 | 0.005 | 0.88  [0.77, 1.01] | 0.060 | 0.178 |  |  | 0.86  [0.76, 0.97] | 0.016 | 0.077 | 0.91  [0.80, 1.03] | 0.126 | 0.565 |
|  | **PHD** |  |  |  |  |  |  |  |  |  |  |  |  |  |  |  |  |
| Q1 | 2  [0, 2.5] | 76 | 476687 | Ref. |  |  | Ref. |  |  | 70 | 488898 | Ref. |  |  | Ref. |  |  |
| Q2 | 3  [2.5, 3] | 44 | 472020 | 0.57  [0.40, 0.83] | 0.003 | 0.031 | 0.61  [0.42, 0.88] | 0.009 | 0.103 | 55 | 483231 | 0.77  [0.54, 1.09] | 0.145 | 0.341 | 0.80  [0.56, 1.14] | 0.216 | 0.592 |
| Q3 | 3.5  [3, 3.67] | 29 | 292037 | 0.62  [0.40, 0.94] | 0.026 | 0.157 | 0.69  [0.45, 1.06] | 0.093 | 0.370 | 33 | 297410 | 0.75  [0.50, 1.14] | 0.180 | 0.341 | 0.82  [0.54, 1.25] | 0.361 | 0.592 |
| Q4 | 4  [3.67, 4.25] | 45 | 377259 | 0.75  [0.52, 1.08] | 0.124 | 0.320 | 0.83  [0.57, 1.20] | 0.326 | 0.586 | 55 | 384591 | 0.98  [0.69, 1.39] | 0.899 | 0.924 | 1.06  [0.74, 1.51] | 0.741 | 0.867 |
| Q5 | 5  [4.25, 9] | 43 | 386897 | 0.71  [0.49, 1.03] | 0.072 | 0.216 | 0.82  [0.56, 1.20] | 0.309 | 0.586 | 44 | 394431 | 0.77  [0.52, 1.12] | 0.167 | 0.341 | 0.86  [0.59, 1.26] | 0.443 | 0.665 |
| P for trend |  |  |  | 0.90  [0.79, 1.02] | 0.103 | 0.155 | 0.95  [0.84, 1.08] | 0.409 | 0.526 |  |  | 0.94  [0.84, 1.06] | 0.326 | 0.488 | 0.98  [0.87, 1.11] | 0.750 | 0.963 |
| per 1 SD |  |  |  | 0.88  [0.77, 1.00] | 0.049 | 0.102 | 0.92  [0.81, 1.05] | 0.244 | 0.313 |  |  | 0.92  [0.81, 1.04] | 0.185 | 0.416 | 0.96  [0.84, 1.09] | 0.500 | 0.901 |
|  | **SMD** |  |  |  |  |  |  |  |  |  |  |  |  |  |  |  |  |
| Q1 | -2.56  [-12.94, -2.03] | 51 | 401037 | Ref. |  |  | Ref. |  |  | 55 | 410450 | Ref. |  |  | Ref. |  |  |
| Q2 | -1.7  [-2.03, -1.44] | 48 | 401456 | 0.96  [0.65, 1.42] | 0.841 | 0.971 | 0.97  [0.65, 1.44] | 0.883 | 0.935 | 58 | 409784 | 1.07  [0.74, 1.55] | 0.709 | 0.823 | 1.13  [0.78, 1.64] | 0.525 | 0.700 |
| Q3 | -1.2  [-1.44, -0.97] | 40 | 402195 | 0.82  [0.54, 1.24] | 0.346 | 0.542 | 0.80  [0.53, 1.22] | 0.308 | 0.586 | 47 | 410202 | 0.89  [0.61, 1.32] | 0.571 | 0.685 | 0.94  [0.63, 1.40] | 0.754 | 0.867 |
| Q4 | -0.73  [-0.97, -0.44] | 38 | 400862 | 0.80  [0.52, 1.21] | 0.287 | 0.492 | 0.73  [0.47, 1.12] | 0.148 | 0.484 | 49 | 409221 | 0.96  [0.65, 1.41] | 0.837 | 0.886 | 0.97  [0.65, 1.44] | 0.868 | 0.893 |
| Q5 | 0  [-0.44, 7,17] | 60 | 399348 | 1.30  [0.89, 1.89] | 0.172 | 0.344 | 1.01  [0.68, 1.49] | 0.959 | 0.959 | 48 | 408905 | 0.98  [0.67, 1.45] | 0.939 | 0.939 | 0.87  [0.58, 1.31] | 0.513 | 0.700 |
| P for trend |  |  |  | 1.07  [0.93, 1.24] | 0.352 | 0.453 | 0.97  [0.84, 1.13] | 0.734 | 0.825 |  |  | 0.98  [0.85, 1.13] | 0.767 | 0.862 | 0.94  [0.81, 1.09] | 0.396 | 0.891 |
| per 1 SD |  |  |  | 1.06  [0.93, 1.21] | 0.362 | 0.465 | 0.97  [0.86, 1.11] | 0.693 | 0.762 |  |  | 0.97  [0.86, 1.10] | 0.649 | 0.700 | 0.94  [0.83, 1.06] | 0.317 | 0.752 |
|  | **LCD** |  |  |  |  |  |  |  |  |  |  |  |  |  |  |  |  |
| Q1 | 2.33  [0, 4] | 49 | 430317 | Ref. |  |  | Ref. |  |  | 45 | 440579 | Ref. |  |  | Ref. |  |  |
| Q2 | 6  [4, 7] | 58 | 422573 | 1.19  [0.81, 1.74] | 0.368 | 0.552 | 1.14  [0.78, 1.67] | 0.500 | 0.720 | 59 | 431900 | 1.32  [0.89, 1.94] | 0.164 | 0.341 | 1.29  [0.88, 1.91] | 0.196 | 0.592 |
| Q3 | 9  [7, 10] | 48 | 426885 | 1.00  [0.67, 1.49] | 0.987 | 0.989 | 0.89  [0.59, 1.32] | 0.552 | 0.736 | 55 | 435786 | 1.25  [0.84, 1.86] | 0.263 | 0.411 | 1.19  [0.80, 1.77] | 0.391 | 0.612 |
| Q4 | 12  [10, 13.5] | 41 | 337266 | 1.10  [0.72, 1.66] | 0.666 | 0.888 | 0.94  [0.62, 1.43] | 0.781 | 0.934 | 49 | 344208 | 1.42  [0.95, 2.13] | 0.088 | 0.311 | 1.31  [0.87, 1.96] | 0.194 | 0.592 |
| Q5 | 17  [13.5, 30] | 41 | 387858 | 1.00  [0.66, 1.51] | 0.989 | 0.989 | 0.80  [0.53, 1.22] | 0.297 | 0.586 | 49 | 396089 | 1.29  [0.86, 1.93] | 0.220 | 0.360 | 1.12  [0.74, 1.68] | 0.596 | 0.766 |
| P for trend |  |  |  | 1.00  [0.97, 1.02] | 0.832 | 0.832 | 0.98  [0.96, 1.01] | 0.169 | 0.304 |  |  | 1.01  [0.99, 1.04] | 0.234 | 0.422 | 1.00  [0.98, 1.03] | 0.704 | 0.963 |
| per 1 SD |  |  |  | 0.96  [0.84, 1.10] | 0.589 | 0.663 | 0.89  [0.78, 1.02] | 0.086 | 0.178 |  |  | 1.05  [0.93, 1.19] | 0.434 | 0.559 | 1.00  [0.88, 1.13] | 0.937 | 0.937 |
|  | **aLCD** |  |  |  |  |  |  |  |  |  |  |  |  |  |  |  |  |
| Q1 | 1  [0, 2.5] | 50 | 416071 | Ref. |  |  | Ref. |  |  | 45 | 425485 | Ref. |  |  | Ref. |  |  |
| Q2 | 4  [2.5, 5] | 46 | 391772 | 0.95  [0.63, 1.41] | 0.789 | 0.947 | 0.88  [0.59, 1.31] | 0.523 | 0.724 | 56 | 399897 | 1.28  [0.86, 1.89] | 0.220 | 0.360 | 1.24  [0.84, 1.84] | 0.281 | 0.592 |
| Q3 | 7  [5, 8] | 39 | 401162 | 0.79  [0.52, 1.20] | 0.262 | 0.472 | 0.67  [0.44, 1.03] | 0.066 | 0.339 | 39 | 409906 | 0.87  [0.57, 1.34] | 0.525 | 0.675 | 0.82  [0.53, 1.26] | 0.362 | 0.592 |
| Q4 | 10  [8, 12] | 52 | 405116 | 1.06  [0.72, 1.56] | 0.771 | 0.947 | 0.83  [0.56, 1.23] | 0.342 | 0.587 | 63 | 413713 | 1.42  [0.97, 2.08] | 0.072 | 0.311 | 1.29  [0.87, 1.89] | 0.205 | 0.592 |
| Q5 | 16  [12, 30] | 50 | 390778 | 1.08  [0.73, 1.60] | 0.691 | 0.888 | 0.78  [0.53, 1.17] | 0.229 | 0.582 | 54 | 399559 | 1.29  [0.87, 1.91] | 0.212 | 0.360 | 1.10  [0.74, 1.65] | 0.631 | 0.783 |
| P for trend |  |  |  | 1.01  [0.98, 1.03] | 0.493 | 0.555 | 0.99  [0.96, 1.01] | 0.302 | 0.453 |  |  | 1.02  [0.99, 1.04] | 0.183 | 0.412 | 1.01  [0.98, 1.03] | 0.652 | 0.963 |
| per 1 SD |  |  |  | 1.02  [0.90, 1.16] | 0.755 | 0.755 | 0.91  [0.79, 1.04] | 0.153 | 0.229 |  |  | 1.07  [0.95, 1.21] | 0.278 | 0.500 | 1.01  [0.89, 1.14] | 0.890 | 0.937 |
|  | **pLCD** |  |  |  |  |  |  |  |  |  |  |  |  |  |  |  |  |
| Q1 | 11  [0, 12.5] | 50 | 416071 | Ref. |  |  | Ref. |  |  | 45 | 425485 | Ref. |  |  | Ref. |  |  |
| Q2 | 14  [12.5, 15] | 46 | 391772 | 0.76  [0.52, 1.11] | 0.161 | 0.344 | 0.78  [0.54, 1.14] | 0.202 | 0.558 | 56 | 399897 | 0.82  [0.56, 1.20] | 0.319 | 0.459 | 0.83  [0.57, 1.21] | 0.326 | 0.592 |
| Q3 | 16.5  [15, 17.5] | 39 | 401162 | 0.76  [0.52, 1.12] | 0.171 | 0.344 | 0.81  [0.55, 1.20] | 0.298 | 0.586 | 39 | 409906 | 0.81  [0.55, 1.20] | 0.299 | 0.449 | 0.83  [0.56, 1.22] | 0.348 | 0.592 |
| Q4 | 19  [17.5, 20] | 52 | 405116 | 0.74  [0.50, 1.08] | 0.113 | 0.312 | 0.80  [0.54, 1.17] | 0.242 | 0.582 | 63 | 413713 | 0.95  [0.66, 1.37] | 0.792 | 0.886 | 0.96  [0.67, 1.39] | 0.844 | 0.893 |
| Q5 | 22  [20, 30] | 50 | 390778 | 0.67  [0.44, 1.01] | 0.056 | 0.216 | 0.69  [0.46, 1.05] | 0.084 | 0.370 | 54 | 399559 | 0.96  [0.65, 1.41] | 0.828 | 0.886 | 0.95  [0.65, 1.40] | 0.805 | 0.878 |
| P for trend |  |  |  | 0.97  [0.93, 1.00] | 0.055 | 0.121 | 0.97  [0.94, 1.01] | 0.110 | 0.247 |  |  | 1.00  [0.97, 1.03] | 0.964 | 0.964 | 1.00  [0.97, 1.03] | 0.963 | 0.963 |
| per 1 SD |  |  |  | 0.88  [0.78, 1.00] | 0.057 | 0.102 | 0.90  [0.79, 1.02] | 0.099 | 0.178 |  |  | 0.98  [0.86, 1.10] | 0.700 | 0.700 | 0.97  [0.86, 1.10] | 0.684 | 0.937 |
|  | **PDI** |  |  |  |  |  |  |  |  |  |  |  |  |  |  |  |  |
| Q1 | 44  [26, 46] | 59 | 428420 | Ref. |  |  | Ref. |  |  | 68 | 439354 | Ref. |  |  | Ref. |  |  |
| Q2 | 48  [46, 49] | 56 | 413963 | 0.99  [0.69, 1.43] | 0.956 | 0.989 | 1.14  [0.79, 1.64] | 0.496 | 0.720 | 51 | 423059 | 0.77  [0.54, 1.11] | 0.165 | 0.341 | 0.82  [0.57, 1.18] | 0.290 | 0.592 |
| Q3 | 50.5  [49, 51.5] | 43 | 361181 | 0.88  [0.60, 1.31] | 0.539 | 0.757 | 1.11  [0.74, 1.65] | 0.609 | 0.783 | 37 | 368809 | 0.65  [0.43, 0.97] | 0.034 | 0.292 | 0.71  [0.48, 1.07] | 0.102 | 0.592 |
| Q4 | 53  [51.5, 54.5] | 41 | 403866 | 0.77  [0.51, 1.14] | 0.193 | 0.366 | 1.01  [0.67, 1.53] | 0.944 | 0.959 | 47 | 411844 | 0.75  [0.52, 1.09] | 0.128 | 0.341 | 0.83  [0.57, 1.22] | 0.347 | 0.592 |
| Q5 | 57  [54.5, 77] | 38 | 397468 | 0.73  [0.49, 1.10] | 0.136 | 0.326 | 1.05  [0.68, 1.60] | 0.830 | 0.934 | 54 | 405496 | 0.88  [0.61, 1.26] | 0.485 | 0.647 | 0.99  [0.68, 1.44] | 0.959 | 0.959 |
| P for trend |  |  |  | 0.97  [0.95, 1.00] | 0.067 | 0.121 | 1.00  [0.97, 1.03] | 0.935 | 0.935 |  |  | 0.99  [0.96, 1.02] | 0.411 | 0.529 | 1.00  [0.97, 1.03] | 0.875 | 0.963 |
| per 1 SD |  |  |  | 0.89  [0.79, 1.02] | 0.087 | 0.130 | 1.02  [0.89, 1.17] | 0.762 | 0.762 |  |  | 0.95  [0.84, 1.07] | 0.395 | 0.559 | 0.99  [0.87, 1.12] | 0.868 | 0.937 |
|  | **hPDI** |  |  |  |  |  |  |  |  |  |  |  |  |  |  |  |  |
| Q1 | 50  [31, 52] | 66 | 425117 | Ref. |  |  | Ref. |  |  | 69 | 435975 | Ref. |  |  | Ref. |  |  |
| Q2 | 54  [52, 55.33] | 41 | 380019 | 0.67  [0.45, 0.99] | 0.045 | 0.202 | 0.68  [0.46, 1.00] | 0.051 | 0.339 | 45 | 388941 | 0.69  [0.47, 1.00] | 0.052 | 0.310 | 0.74  [0.50, 1.07] | 0.112 | 0.592 |
| Q3 | 57  [55.33, 58] | 55 | 410827 | 0.83  [0.58, 1.20] | 0.325 | 0.532 | 0.86  [0.59, 1.24] | 0.412 | 0.669 | 51 | 419692 | 0.72  [0.50, 1.03] | 0.071 | 0.311 | 0.79  [0.55, 1.15] | 0.220 | 0.592 |
| Q4 | 60  [58, 61.5] | 36 | 388533 | 0.59  [0.39, 0.89] | 0.013 | 0.090 | 0.62  [0.41, 0.94] | 0.024 | 0.218 | 48 | 396131 | 0.73  [0.50, 1.05] | 0.091 | 0.311 | 0.83  [0.56, 1.21] | 0.333 | 0.592 |
| Q5 | 64  [61.5, 83] | 39 | 400403 | 0.65  [0.44, 0.98] | 0.039 | 0.199 | 0.71  [0.47, 1.08] | 0.108 | 0.390 | 44 | 407823 | 0.67  [0.46, 0.98] | 0.041 | 0.292 | 0.80  [0.53, 1.18] | 0.260 | 0.592 |
| P for trend |  |  |  | 0.97  [0.94, 1.00] | 0.025 | 0.076 | 0.98  [0.95, 1.00] | 0.083 | 0.247 |  |  | 0.97  [0.95, 1.00] | 0.056 | 0.167 | 0.99  [0.96, 1.01] | 0.352 | 0.891 |
| per 1 SD |  |  |  | 0.85  [0.74, 0.97] | 0.014 | 0.042 | 0.87  [0.75, 1.00] | 0.049 | 0.178 |  |  | 0.88  [0.77, 1.00] | 0.043 | 0.130 | 0.94  [0.82, 1.07] | 0.334 | 0.752 |
|  | **uPDI** |  |  |  |  |  |  |  |  |  |  |  |  |  |  |  |  |
| Q1 | 50  [28, 52] | 43 | 424534 | Ref. |  |  | Ref. |  |  | 47 | 433587 | Ref. |  |  | Ref. |  |  |
| Q2 | 54  [52, 55.5] | 39 | 395312 | 0.99  [0.64, 1.53] | 0.970 | 0.989 | 0.97  [0.63, 1.49] | 0.877 | 0.935 | 48 | 403874 | 1.13  [0.76, 1.69] | 0.550 | 0.683 | 1.14  [0.76, 1.71] | 0.517 | 0.700 |
| Q3 | 57  [55.5, 58] | 43 | 389807 | 1.14  [0.75, 1.74] | 0.547 | 0.757 | 1.07  [0.70, 1.64] | 0.758 | 0.934 | 53 | 398180 | 1.31  [0.89, 1.94] | 0.175 | 0.341 | 1.32  [0.89, 1.96] | 0.173 | 0.592 |
| Q4 | 60  [58, 61.5] | 39 | 399512 | 1.04  [0.67, 1.60] | 0.863 | 0.971 | 0.95  [0.61, 1.47] | 0.811 | 0.934 | 53 | 408193 | 1.33  [0.90, 1.97] | 0.154 | 0.341 | 1.32  [0.89, 1.97] | 0.166 | 0.592 |
| Q5 | 64  [61.5, 80] | 73 | 395734 | 2.16  [1.48, 3.16] | <0.001 | 0.003 | 1.88  [1.28, 2.78] | 0.001 | 0.048 | 56 | 404727 | 1.59  [1.08, 2.35] | 0.020 | 0.292 | 1.53  [1.03, 2.27] | 0.037 | 0.592 |
| P for trend |  |  |  | 1.06  [1.03, 1.08] | <0.001 | 7.70E-04 | 1.05  [1.02, 1.07] | 0.002 | 0.014 |  |  | 1.03  [1.01, 1.06] | 0.014 | 0.070 | 1.03  [1.00, 1.06] | 0.028 | 0.249 |
| per 1 SD |  |  |  | 1.32  [1.15, 1.50] | <0.001 | 4.28E-04 | 1.25  [1.09, 1.43] | 0.001 | 0.009 |  |  | 1.17  [1.03, 1.32] | 0.017 | 0.077 | 1.15  [1.01, 1.30] | 0.034 | 0.307 |

The nine dietary pattern scores were divided into quintiles and treated as categorical variables. Model 1 was adjusted for age at first completion of the Oxford WebQ and sex, and Model 2 was further adjusted for race, educational attainment, TDI, family history of cancer, smoking status, average alcohol intake (except aMED & SMD), MET, average energy intake, BMI, WHR and T2DM. The *P*_adjusted_ was determined using a multiple comparison test based on the false discovery rate (FDR).

HR, hazard ratio; CI, confidence interval; aMED, alternate Mediterranean diet; PHD, Planetary Health Diet; SMD, sulfur microbial diet; LCD, Low-carbohydrate Diet; aLCD, animal based Low-carbohydrate Diet; pLCD, plant based Low-carbohydrate Diet; PDI, Plant-based Diet Index; hPDI, healthful Plant-based Diet Index; uPDI, unhealthful Plant-based Diet Index.

**Table S8. The associations of the scores of dietary patterns with the incidence of** **hepatocellular carcinoma and intrahepatic cholangiocarcinoma in the UK Biobank cohort.**

|  | Median  (Score range) | **Hepatocellular carcinoma (HCC)** | | | | | | | | **Intrahepatic cholangiocarcinoma（ICC）** | | | | | | | |
| --- | --- | --- | --- | --- | --- | --- | --- | --- | --- | --- | --- | --- | --- | --- | --- | --- | --- |
|  |  | Cases | Person-years | Model 1 HR (95% CI) | Model 1 *P* | Model 1 *P*_adjusted_ | Model 2 HR (95% CI) | Model 2 *P* | Model 2 *P*_adjusted_ | Cases | Person-years | Model1 HR (95% CI) | Model 1 *P* | Model 1 *P*_adjusted_ | Model 2 HR  (95% CI) | Model 2 *P* | Model 2 *P*_adjusted_ |
|  | **aMED** |  |  |  |  |  |  |  |  |  |  |  |  |  |  |  |  |
| Q1 | 2  [0, 2] | 24 | 413534 | Ref. |  |  | Ref. |  |  | 32 | 413581 | Ref. |  |  | Ref. |  |  |
| Q2 | 3  [2, 3] | 24 | 513181 | 0.75  [0.42, 1.32] | 0.312 | 0.627 | 0.83  [0.47, 1.46] | 0.514 | 0.925 | 33 | 513230 | 0.77  [0.48, 1.26] | 0.304 | 0.730 | 0.79  [0.49, 1.29] | 0.345 | 0.819 |
| Q3 | 4  [3, 4] | 18 | 539055 | 0.52  [0.28, 0.96] | 0.036 | 0.282 | 0.60  [0.33, 1.11] | 0.106 | 0.737 | 35 | 539157 | 0.76  [0.47, 1.22] | 0.256 | 0.730 | 0.79  [0.49, 1.28] | 0.333 | 0.819 |
| Q4 | 5  [4, 5] | 20 | 360964 | 0.83  [0.46, 1.51] | 0.550 | 0.747 | 1.03  [0.56, 1.88] | 0.928 | 0.986 | 21 | 360956 | 0.66  [0.38, 1.14] | 0.133 | 0.660 | 0.69  [0.40, 1.21] | 0.201 | 0.814 |
| Q5 | 6  [5, 9] | 7 | 220839 | 0.45  [0.19, 1.04] | 0.063 | 0.282 | 0.59  [0.25, 1.39] | 0.224 | 0.737 | 8 | 220841 | 0.39  [0.18, 0.84] | 0.017 | 0.336 | 0.42  [0.19, 0.93] | 0.033 | 0.467 |
| P for trend |  |  |  | 0.87  [0.74, 1.03] | 0.101 | 0.380 | 0.93  [0.79, 1.10] | 0.400 | 0.709 |  |  | 0.84  [0.73, 0.97] | 0.015 | 0.084 | 0.86  [0.74, 0.99] | 0.037 | 0.167 |
| per 1 SD |  |  |  | 0.85  [0.69, 1.05] | 0.134 | 0.395 | 0.93  [0.75, 1.14] | 0.479 | 0.863 |  |  | 0.79  [0.66, 0.94] | 0.009 | 0.083 | 0.81  [0.68, 0.97] | 0.023 | 0.158 |
|  | **PHD** |  |  |  |  |  |  |  |  |  |  |  |  |  |  |  |  |
| Q1 | 2  [0, 2.5] | 27 | 488622 | Ref. |  |  | Ref. |  |  | 32 | 488642 | Ref. |  |  | Ref. |  |  |
| Q2 | 3  [2.5, 3] | 19 | 483016 | 0.69  [0.38, 1.24] | 0.217 | 0.602 | 0.75  [0.41, 1.34] | 0.329 | 0.738 | 29 | 483069 | 0.88  [0.53, 1.45] | 0.612 | 0.847 | 0.90  [0.54, 1.48] | 0.666 | 0.883 |
| Q3 | 3.5  [3, 3.67] | 10 | 297278 | 0.60  [0.29, 1.24] | 0.166 | 0.545 | 0.70  [0.34, 1.46] | 0.344 | 0.738 | 18 | 297322 | 0.88  [0.49, 1.57] | 0.668 | 0.891 | 0.92  [0.52, 1.64] | 0.780 | 0.924 |
| Q4 | 4  [3.67, 4.25] | 21 | 384373 | 0.99  [0.56, 1.75] | 0.961 | 0.993 | 1.15  [0.65, 2.04] | 0.637 | 0.939 | 30 | 384447 | 1.14  [0.69, 1.88] | 0.609 | 0.847 | 1.18  [0.71, 1.95] | 0.518 | 0.869 |
| Q5 | 5  [4.25, 9] | 16 | 394284 | 0.74  [0.40, 1.38] | 0.347 | 0.627 | 0.92  [0.49, 1.72] | 0.790 | 0.986 | 20 | 394284 | 0.74  [0.42, 1.29] | 0.284 | 0.730 | 0.77  [0.44, 1.35] | 0.364 | 0.819 |
| P for trend |  |  |  | 0.94  [0.77, 1.14] | 0.526 | 0.527 | 1.01  [0.83, 1.23] | 0.931 | 0.931 |  |  | 0.95  [0.80, 1.12] | 0.513 | 0.704 | 0.96  [0.81, 1.14] | 0.639 | 0.955 |
| per 1 SD |  |  |  | 0.88  [0.72, 1.09] | 0.248 | 0.395 | 0.95  [0.77, 1.18] | 0.655 | 0.886 |  |  | 0.92  [0.77, 1.10] | 0.349 | 0.628 | 0.93  [0.78, 1.11] | 0.446 | 0.669 |
|  | **SMD** |  |  |  |  |  |  |  |  |  |  |  |  |  |  |  |  |
| Q1 | -2.56  [-12.94, -2.03] | 17 | 410228 | Ref. |  |  | Ref. |  |  | 26 | 410276 | Ref. |  |  | Ref. |  |  |
| Q2 | -1.7  [-2.03, -1.44] | 21 | 409528 | 1.27  [0.67, 2.41] | 0.462 | 0.736 | 1.45  [0.76, 2.76] | 0.263 | 0.737 | 31 | 409597 | 1.20  [0.71, 2.02] | 0.496 | 0.812 | 1.22  [0.72, 2.07] | 0.451 | 0.869 |
| Q3 | -1.2  [-1.44, -0.97] | 16 | 410035 | 1.00  [0.51, 1.99] | 0.993 | 0.993 | 1.16  [0.58, 2.32] | 0.678 | 0.939 | 25 | 410077 | 0.99  [0.57, 1.71] | 0.971 | 0.983 | 1.00  [0.57, 1.76] | 0.990 | 0.990 |
| Q4 | -0.73  [-0.97, -0.44] | 21 | 409058 | 1.36  [0.72, 2.58] | 0.348 | 0.627 | 1.49  [0.77, 2.87] | 0.238 | 0.737 | 26 | 409051 | 1.07  [0.62, 1.83] | 0.820 | 0.923 | 1.04  [0.60, 1.82] | 0.887 | 0.924 |
| Q5 | 0  [-0.44, 7,17] | 18 | 408724 | 1.22  [0.63, 2.36] | 0.561 | 0.747 | 1.07  [0.54, 2.13] | 0.850 | 0.986 | 21 | 408764 | 0.91  [0.51, 1.62] | 0.747 | 0.912 | 0.83  [0.46, 1.50] | 0.528 | 0.869 |
| P for trend |  |  |  | 1.08  [0.85, 1.37] | 0.527 | 0.527 | 1.03  [0.81, 1.30] | 0.823 | 0.709 |  |  | 0.96  [0.78, 1.17] | 0.684 | 0.704 | 0.93  [0.75, 1.14] | 0.462 | 0.831 |
| per 1 SD |  |  |  | 1.03  [0.84, 1.26] | 0.800 | 0.800 | 0.99  [0.81, 1.20] | 0.886 | 0.886 |  |  | 0.96  [0.80, 1.14] | 0.621 | 0.699 | 0.93  [0.78, 1.11] | 0.410 | 0.669 |
|  | **LCD** |  |  |  |  |  |  |  |  |  |  |  |  |  |  |  |  |
| Q1 | 2.33  [0, 4] | 18 | 440424 | Ref. |  |  | Ref. |  |  | 22 | 440415 | Ref. |  |  | Ref. |  |  |
| Q2 | 6  [4, 7] | 22 | 431674 | 1.23  [0.66, 2.30] | 0.511 | 0.736 | 1.19  [0.64, 2.22] | 0.588 | 0.939 | 30 | 431709 | 1.36  [0.79, 2.36] | 0.270 | 0.730 | 1.36  [0.78, 2.36] | 0.275 | 0.819 |
| Q3 | 9  [7, 10] | 18 | 435573 | 1.04  [0.54, 2.01] | 0.897 | 0.993 | 0.95  [0.50, 1.84] | 0.889 | 0.986 | 26 | 435617 | 1.19  [0.68, 2.11] | 0.543 | 0.847 | 1.16  [0.66, 2.05] | 0.611 | 0.869 |
| Q4 | 12  [10, 13.5] | 9 | 343959 | 0.67  [0.30, 1.49] | 0.324 | 0.627 | 0.58  [0.26, 1.29] | 0.179 | 0.737 | 32 | 344118 | 1.86  [1.08, 3.21] | 0.025 | 0.336 | 1.78  [1.03, 3.07] | 0.039 | 0.467 |
| Q5 | 17  [13.5, 30] | 26 | 395943 | 1.78  [0.97, 3.25] | 0.061 | 0.282 | 1.38  [0.75, 2.54] | 0.294 | 0.738 | 19 | 395905 | 0.99  [0.54, 1.84] | 0.983 | 0.983 | 0.91  [0.49, 1.69] | 0.765 | 0.924 |
| P for trend |  |  |  | 1.03  [0.99, 1.07] | 0.162 | 0.380 | 1.01  [0.97, 1.05] | 0.592 | 0.709 |  |  | 1.01  [0.97, 1.04] | 0.704 | 0.704 | 1.00  [0.97, 1.04] | 0.981 | 0.981 |
| per 1 SD |  |  |  | 1.14  [0.94, 1.40] | 0.190 | 0.395 | 1.04  [0.85, 1.27] | 0.702 | 0.886 |  |  | 1.01  [0.84, 1.20] | 0.950 | 0.950 | 0.97  [0.81, 1.16] | 0.752 | 0.957 |
|  | **aLCD** |  |  |  |  |  |  |  |  |  |  |  |  |  |  |  |  |
| Q1 | 1  [0, 2.5] | 19 | 425358 | Ref. |  |  | Ref. |  |  | 21 | 425314 | Ref. |  |  | Ref. |  |  |
| Q2 | 4  [2.5, 5] | 19 | 399643 | 1.02  [0.54, 1.93] | 0.944 | 0.993 | 0.98  [0.52, 1.86] | 0.959 | 0.986 | 28 | 399720 | 1.37  [0.78, 2.41] | 0.275 | 0.730 | 1.35  [0.77, 2.38] | 0.300 | 0.819 |
| Q3 | 7  [5, 8] | 13 | 409749 | 0.69  [0.34, 1.39] | 0.295 | 0.627 | 0.62  [0.31, 1.27] | 0.192 | 0.737 | 23 | 409795 | 1.10  [0.61, 1.98] | 0.760 | 0.847 | 1.06  [0.58, 1.92] | 0.860 | 0.924 |
| Q4 | 10  [8, 12] | 16 | 413440 | 0.86  [0.44, 1.68] | 0.664 | 0.853 | 0.73  [0.37, 1.44] | 0.363 | 0.738 | 32 | 413527 | 1.53  [0.88, 2.66] | 0.127 | 0.336 | 1.44  [0.82, 2.51] | 0.204 | 0.814 |
| Q5 | 16  [12, 30] | 26 | 399383 | 1.49  [0.82, 2.69] | 0.186 | 0.558 | 1.17  [0.64, 2.14] | 0.608 | 0.939 | 25 | 399409 | 1.26  [0.71, 2.25] | 0.435 | 0.983 | 1.13  [0.63, 2.03] | 0.687 | 0.883 |
| P for trend |  |  |  | 1.03  [0.99, 1.07] | 0.169 | 0.380 | 1.01  [0.97, 1.05] | 0.585 | 0.709 |  |  | 1.01  [0.98, 1.05] | 0.454 | 0.704 | 1.00  [0.97, 1.04] | 0.774 | 0.955 |
| per 1 SD |  |  |  | 1.12  [0.92, 1.36] | 0.263 | 0.395 | 1.02  [0.83, 1.25] | 0.835 | 0.886 |  |  | 1.05  [0.88, 1.24] | 0.589 | 0.699 | 1.00  [0.84, 1.20] | 0.961 | 0.961 |
|  | **pLCD** |  |  |  |  |  |  |  |  |  |  |  |  |  |  |  |  |
| Q1 | 11  [0, 12.5] | 19 | 425358 | Ref. |  |  | Ref. |  |  | 21 | 425314 | Ref. |  |  | Ref. |  |  |
| Q2 | 14  [12.5, 15] | 19 | 399643 | 0.88  [0.45, 1.72] | 0.708 | 0.879 | 0.87  [0.44, 1.70] | 0.676 | 0.939 | 28 | 399720 | 0.87  [0.53, 1.44] | 0.596 | 0.847 | 0.88  [0.54, 1.46] | 0.627 | 0.869 |
| Q3 | 16.5  [15, 17.5] | 13 | 409749 | 1.01  [0.52, 1.96] | 0.979 | 0.993 | 1.01  [0.52, 1.96] | 0.978 | 0.986 | 23 | 409795 | 0.68  [0.39, 1.17] | 0.165 | 0.660 | 0.70  [0.40, 1.21] | 0.197 | 0.814 |
| Q4 | 19  [17.5, 20] | 16 | 413440 | 1.44  [0.79, 2.62] | 0.238 | 0.612 | 1.41  [0.77, 2.58] | 0.266 | 0.737 | 32 | 413527 | 0.68  [0.40, 1.17] | 0.162 | 0.660 | 0.70  [0.41, 1.20] | 0.194 | 0.814 |
| Q5 | 22  [20, 30] | 26 | 399383 | 1.09  [0.56, 2.14] | 0.801 | 0.961 | 1.02  [0.52, 2.02] | 0.943 | 0.986 | 25 | 399409 | 0.83  [0.49, 1.41] | 0.492 | 0.812 | 0.84  [0.49, 1.43] | 0.520 | 0.869 |
| P for trend |  |  |  | 1.03  [0.97, 1.08] | 0.347 | 0.511 | 1.02  [0.97, 1.08] | 0.445 | 0.709 |  |  | 0.97  [0.93, 1.02] | 0.270 | 0.608 | 0.98  [0.93, 1.02] | 0.303 | 0.772 |
| per 1 SD |  |  |  | 1.10  [0.90, 1.36] | 0.353 | 0.453 | 1.08  [0.88, 1.33] | 0.466 | 0.863 |  |  | 0.89  [0.75, 1.06] | 0.208 | 0.533 | 0.90  [0.75, 1.07] | 0.233 | 0.669 |
|  | **PDI** |  |  |  |  |  |  |  |  |  |  |  |  |  |  |  |  |
| Q1 | 44  [26, 46] | 29 | 439113 | Ref. |  |  | Ref. |  |  | 29 | 439085 | Ref. |  |  | Ref. |  |  |
| Q2 | 48  [46, 49] | 18 | 422886 | 0.64  [0.36, 1.16] | 0.143 | 0.514 | 0.70  [0.39, 1.27] | 0.243 | 0.737 | 26 | 422934 | 0.91  [0.54, 1.55] | 0.733 | 0.912 | 0.96  [0.56, 1.63] | 0.866 | 0.924 |
| Q3 | 50.5  [49, 51.5] | 12 | 368659 | 0.50  [0.26, 0.98] | 0.045 | 0.282 | 0.57  [0.29, 1.13] | 0.109 | 0.737 | 24 | 368728 | 0.96  [0.56, 1.66] | 0.892 | 0.944 | 1.04  [0.60, 1.79] | 0.899 | 0.924 |
| Q4 | 53  [51.5, 54.5] | 13 | 411630 | 0.50  [0.26, 0.96] | 0.037 | 0.282 | 0.57  [0.30, 1.12] | 0.103 | 0.737 | 29 | 411735 | 1.05  [0.63, 1.76] | 0.847 | 0.924 | 1.15  [0.68, 1.94] | 0.612 | 0.869 |
| Q5 | 57  [54.5, 77] | 21 | 405286 | 0.83  [0.47, 1.45] | 0.506 | 0.736 | 0.94  [0.52, 1.68] | 0.823 | 0.986 | 21 | 405282 | 0.78  [0.44, 1.36] | 0.378 | 0.800 | 0.86  [0.48, 1.54] | 0.609 | 0.869 |
| P for trend |  |  |  | 0.98  [0.93, 1.02] | 0.299 | 0.511 | 0.99  [0.94, 1.03] | 0.564 | 0.709 |  |  | 0.99  [0.95, 1.03] | 0.549 | 0.704 | 1.00  [0.96, 1.04] | 0.848 | 0.955 |
| per 1 SD |  |  |  | 0.87  [0.71, 1.06] | 0.168 | 0.395 | 0.91  [0.74, 1.12] | 0.372 | 0.863 |  |  | 0.95  [0.80, 1.13] | 0.542 | 0.699 | 0.98  [0.82, 1.18] | 0.851 | 0.957 |
|  | **hPDI** |  |  |  |  |  |  |  |  |  |  |  |  |  |  |  |  |
| Q1 | 50  [31, 52] | 30 | 435736 | Ref. |  |  | Ref. |  |  | 30 | 435730 | Ref. |  |  | Ref. |  |  |
| Q2 | 54  [52, 55.33] | 15 | 388750 | 0.53  [0.29, 0.99] | 0.046 | 0.282 | 0.61  [0.33, 1.14] | 0.120 | 0.737 | 23 | 388783 | 0.80  [0.46, 1.37] | 0.411 | 0.812 | 0.81  [0.47, 1.40] | 0.442 | 0.869 |
| Q3 | 57  [55.33, 58] | 17 | 419482 | 0.56  [0.31, 1.02] | 0.056 | 0.282 | 0.70  [0.38, 1.28] | 0.244 | 0.737 | 30 | 419580 | 0.94  [0.56, 1.56] | 0.802 | 0.923 | 0.96  [0.57, 1.61] | 0.873 | 0.924 |
| Q4 | 60  [58, 61.5] | 14 | 395944 | 0.51  [0.27, 0.96] | 0.037 | 0.282 | 0.66  [0.34, 1.28] | 0.222 | 0.737 | 25 | 395991 | 0.83  [0.48, 1.41] | 0.489 | 0.812 | 0.86  [0.49, 1.48] | 0.579 | 0.869 |
| Q5 | 64  [61.5, 83] | 17 | 407660 | 0.64  [0.35, 1.16] | 0.143 | 0.514 | 0.90  [0.48, 1.68] | 0.730 | 0.974 | 21 | 407680 | 0.68  [0.39, 1.21] | 0.190 | 0.684 | 0.72  [0.40, 1.28] | 0.262 | 0.819 |
| P for trend |  |  |  | 0.96  [0.92, 1.01] | 0.097 | 0.380 | 0.99  [0.95, 1.03] | 0.630 | 0.709 |  |  | 0.98  [0.94, 1.02] | 0.247 | 0.608 | 0.98  [0.94, 1.02] | 0.343 | 0.772 |
| per 1 SD |  |  |  | 0.81  [0.65, 1.00] | 0.047 | 0.395 | 0.92  [0.74, 1.15] | 0.477 | 0.863 |  |  | 0.90  [0.75, 1.07] | 0.237 | 0.533 | 0.91  [0.75, 1.10] | 0.335 | 0.669 |
|  | **uPDI** |  |  |  |  |  |  |  |  |  |  |  |  |  |  |  |  |
| Q1 | 50  [28, 52] | 23 | 433459 | Ref. |  |  | Ref. |  |  | 20 | 433426 | Ref. |  |  | Ref. |  |  |
| Q2 | 54  [52, 55.5] | 15 | 403689 | 0.72  [0.37, 1.38] | 0.318 | 0.627 | 0.74  [0.39, 1.42] | 0.369 | 0.738 | 24 | 403721 | 1.34  [0.74, 2.42] | 0.336 | 0.756 | 1.34  [0.74, 2.44] | 0.330 | 0.819 |
| Q3 | 57  [55.5, 58] | 15 | 397925 | 0.75  [0.39, 1.45] | 0.395 | 0.678 | 0.78  [0.41, 1.51] | 0.463 | 0.877 | 29 | 398020 | 1.71  [0.97, 3.03] | 0.065 | 0.547 | 1.71  [0.96, 3.03] | 0.068 | 0.604 |
| Q4 | 60  [58, 61.5] | 19 | 407956 | 0.97  [0.53, 1.78] | 0.916 | 0.993 | 0.99  [0.54, 1.84] | 0.986 | 0.986 | 28 | 408036 | 1.68  [0.95, 2.99] | 0.076 | 0.547 | 1.67  [0.93, 2.98] | 0.084 | 0.604 |
| Q5 | 64  [61.5, 80] | 21 | 404544 | 1.23  [0.68, 2.22] | 0.503 | 0.736 | 1.18  [0.65, 2.17] | 0.584 | 0.939 | 28 | 404562 | 1.91  [1.07, 3.41] | 0.028 | 0.336 | 1.87  [1.04, 3.36] | 0.036 | 0.467 |
| P for trend |  |  |  | 1.02  [0.98, 1.06] | 0.398 | 0.511 | 1.02  [0.97, 1.06] | 0.451 | 0.709 |  |  | 1.04  [1.01, 1.08] | 0.019 | 0.084 | 1.04  [1.00, 1.08] | 0.027 | 0.167 |
| per 1 SD |  |  |  | 1.09  [0.88, 1.34] | 0.432 | 0.486 | 1.08  [0.88, 1.33] | 0.478 | 0.863 |  |  | 1.23  [1.03, 1.47] | 0.025 | 0.112 | 1.22  [1.01, 1.46] | 0.035 | 0.158 |

The nine dietary pattern scores were divided into quintiles and treated as categorical variables. Model 1 was adjusted for age at first completion of the Oxford WebQ and sex, and Model 2 was further adjusted for race, educational attainment, TDI, family history of cancer, smoking status, average alcohol intake (except aMED & SMD), MET, average energy intake, BMI, WHR and T2DM. The *P*_adjusted_ was determined using a multiple comparison test based on the false discovery rate (FDR).

HR, hazard ratio; CI, confidence interval; aMED, alternate Mediterranean diet; PHD, Planetary Health Diet; SMD, sulfur microbial diet; LCD, Low-carbohydrate Diet; aLCD, animal based Low-carbohydrate Diet; pLCD, plant based Low-carbohydrate Diet; PDI, Plant-based Diet Index; hPDI, healthful Plant-based Diet Index; uPDI, unhealthful Plant-based Diet Index.

**Table S9. The *p*-nonlinearity of the nonlinear associations between dietary pattern scores and the incidence and mortality of liver diseases.**

|  | **CLD** | **SLD** | **LC** | **HCC** | **ICC** | **LRD** |
| --- | --- | --- | --- | --- | --- | --- |
| **aMED** | 0.166 | 0.499 | 0.133 | 0.203 | 0.549 | 0.206 |
| **PHD** | 0.131 | 0.100 | 0.397 | 0.169 | 0.899 | 0.074 |
| **SMD** | 0.193 | 0.089 | 0.341 | 0. 094 | 0.700 | 0.113 |
| **LCD** | 0.001 | 0.005 | 0.107 | 0.455 | 0.095 | 0.769 |
| **aLCD** | 0.681 | 0.251 | 0.115 | 0.114 | 0.100 | 0.263 |
| **pLCD** | 0.385 | 0.792 | 0.248 | 0.375 | 0.874 | 0.708 |
| **PDI** | 0.076 | 0.037 | 0.046 | 0.095 | 0.787 | 0.148 |
| **hPDI** | 0.496 | 0. 446 | 0.525 | 0.197 | 0.595 | 0.233 |
| **uPDI** | 0.351 | 0.396 | 0.577 | 0.330 | 0.164 | 0.240 |

The *p*-nonlinearity values for the potential non-linear relationship between dietary pattern scores and the incidence and mortality of liver diseases were assessed using Restricted Cubic Spline (RCS) analysis using a fully adjusted model that accounted for age at first completion of the Oxford WebQ, sex, race, educational attainment, TDI, family history of cancer, smoking status, average alcohol intake (except aMED & SMD), MET, average energy intake, BMI, WHR and T2DM.

CLD, chronic liver disease; SLD, severe liver disease; LC, liver cancer; HCC, hepatocellular carcinoma; ICC, intrahepatic cholangiocarcinoma; LRD, liver-related death; aMED, alternate Mediterranean diet; PHD, Planetary Health Diet; SMD, sulfur microbial diet; LCD, Low-carbohydrate Diet; aLCD, animal based Low-carbohydrate Diet; pLCD, plant based Low-carbohydrate Diet; PDI, Plant-based Diet Index; hPDI, healthful Plant-based Diet Index; uPDI, unhealthful Plant-based Diet Index.

**Table S10. The associations of the scores of dietary patterns with the incidence of chronic liver disease according to MAFLD status.**

|  | **MAFLD (59,473)** | | | | | | | **Non-MAFLD (117,211)** | | | | | | | ***p*-heterogeneity** |
| --- | --- | --- | --- | --- | --- | --- | --- | --- | --- | --- | --- | --- | --- | --- | --- |
|  | Median | Cases | Person-years | Model 1  HR (95% CI) | Model 1 *P* | Model 2  HR (95% CI) | Model 2 *P* | Median | Cases | Person-years | Model 1 HR  (95% CI) | Model 1 *P* | Model 2  HR (95% CI) | Model 2 *P* |  |
|  | **aMED** |  |  |  |  |  |  | **aMED** |  |  |  |  |  |  |  |
| Q1 | 2 | 139 | 170499 | Ref. |  | Ref. |  | 2 | 41 | 242699 | Ref. |  | Ref. |  |  |
| Q2 | 3 | 113 | 187018 | 0.71 [0.55, 0.91] | 0.007 | 0.74 [0.58, 0.95] | 0.020 | 3 | 31 | 325977 | 0.55 [0.34, 0.88] | 0.012 | 0.60 [0.37, 0.96] | 0.032 | 0.444 |
| Q3 | 4 | 87 | 174921 | 0.57 [0.44, 0.75] | <0.001 | 0.61 [0.47, 0.80] | <0.001 | 4 | 39 | 364112 | 0.61 [0.39, 0.95] | 0.028 | 0.69 [0.44, 1.07] | 0.096 | 0.641 |
| Q4 | 5 | 49 | 103823 | 0.53 [0.39, 0.74] | <0.001 | 0.59 [0.42, 0.81] | 0.001 | 5 | 25 | 257232 | 0.55 [0.33, 0.90] | 0.017 | 0.63 [0.38, 1.05] | 0.075 | 0.832 |
| Q5 | 6 | 19 | 52886 | 0.39 [0.24, 0.64] | <0.001 | 0.44 [0.27, 0.72] | 0.001 | 6 | 19 | 167902 | 0.62 [0.36, 1.07] | 0.084 | 0.74 [0.42, 1.29] | 0.286 | 0.172 |
| P for trend |  |  |  | 0.80 [0.73, 0.87] | <0.001 | 0.82 [0.75, 0.89] | <0.001 |  |  |  | 0.89 [0.78, 1.01] | 0.063 | 0.93 [0.81, 1.05] | 0.243 | 0.112 |
| per 1 SD |  |  |  | 0.80 [0.72, 0.88] | <0.001 | 0.83 [0.75, 0.92] | <0.001 |  |  |  | 0.85 [0.72, 0.99] | 0.043 | 0.90 [0.76, 1.05] | 0.186 | 0.407 |
|  | **PHD** |  |  |  |  |  |  | **PHD** |  |  |  |  |  |  |  |
| Q1 | 2 | 159 | 196123 | Ref. |  | Ref. |  | 2 | 40 | 292119 | Ref. |  | Ref. |  |  |
| Q2 | 3 | 88 | 173186 | 0.61 [0.47, 0.80] | <0.001 | 0.64 [0.49, 0.83] | 0.001 | 3 | 29 | 309753 | 0.68 [0.42, 1.10] | 0.113 | 0.73 [0.45, 1.17] | 0.192 | 0.637 |
| Q3 | 3.5 | 46 | 92591 | 0.59 [0.42, 0.82] | 0.002 | 0.65 [0.46, 0.90] | 0.010 | 3.5 | 24 | 204645 | 0.86 [0.52, 1.42] | 0.547 | 0.97 [0.58, 1.62] | 0.907 | 0.201 |
| Q4 | 4 | 64 | 119791 | 0.64 [0.48, 0.86] | 0.003 | 0.69 [0.52, 0.93] | 0.014 | 4 | 38 | 264565 | 1.05 [0.67, 1.64] | 0.820 | 1.16 [0.74, 1.81] | 0.518 | 0.056 |
| Q5 | 5 | 50 | 107457 | 0.56 [0.41, 0.77] | <0.001 | 0.61 [0.44, 0.84] | 0.003 | 5 | 24 | 286839 | 0.62 [0.37, 1.02] | 0.061 | 0.70 [0.42, 1.16] | 0.165 | 0.654 |
| P for trend |  |  |  | 0.81 [0.73, 0.90] | <0.001 | 0.84 [0.76, 0.93] | 0.001 |  |  |  | 0.91 [0.78, 1.06] | 0.231 | 0.95 [0.81, 1.11] | 0.501 | 0.197 |
| per 1 SD |  |  |  | 0.81 [0.73, 0.90] | <0.001 | 0.84 [0.76, 0.93] | 0.001 |  |  |  | 0.86 [0.73, 1.02] | 0.077 | 0.90 [0.77, 1.06] | 0.220 | 0.474 |
|  | **SMD** |  |  |  |  |  |  | **SMD** |  |  |  |  |  |  |  |
| Q1 | -2.6 | 74 | 136254 | Ref. |  | Ref. |  | -2.54 | 33 | 273946 | Ref. |  | Ref. |  |  |
| Q2 | -1.7 | 63 | 123170 | 0.95 [0.68, 1.33] | 0.760 | 0.98 [0.70, 1.38] | 0.922 | -1.69 | 28 | 286412 | 0.82 [0.49, 1.36] | 0.437 | 0.86 [0.52, 1.42] | 0.551 | 0.673 |
| Q3 | -1.2 | 65 | 124473 | 0.98 [0.70, 1.37] | 0.903 | 1.01 [0.72, 1.42] | 0.949 | -1.2 | 34 | 285512 | 1.01 [0.63, 1.63] | 0.965 | 1.05 [0.65, 1.72] | 0.832 | 0.898 |
| Q4 | -0.72 | 76 | 134864 | 1.07 [0.78, 1.48] | 0.663 | 1.08 [0.78, 1.50] | 0.657 | -0.74 | 34 | 274058 | 1.06 [0.66, 1.72] | 0.801 | 1.08 [0.66, 1.77] | 0.769 | 1.000 |
| Q5 | 0.03 | 129 | 170386 | 1.48 [1.11, 1.98] | 0.007 | 1.36 [1.01, 1.83] | 0.045 | -0.03 | 26 | 237993 | 0.95 [0.57, 1.59] | 0.853 | 0.91 [0.53, 1.54] | 0.714 | 0.197 |
| P for trend |  |  |  | 1.18 [1.06, 1.32] | 0.003 | 1.14 [1.01, 1.27] | 0.027 |  |  |  | 1.02 [0.84, 1.23] | 0.848 | 1.00 [0.82, 1.21] | 0.989 | 0.279 |
| per 1 SD |  |  |  | 1.19 [1.08, 1.30] | <0.001 | 1.14 [1.04, 1.25] | 0.004 |  |  |  | 1.07 [0.90, 1.27] | 0.434 | 1.05 [0.89, 1.25] | 0.542 | 0.404 |
|  | **LCD** |  |  |  |  |  |  | **LCD** |  |  |  |  |  |  |  |
| Q1 | 2.33 | 66 | 141881 | Ref. |  | Ref. |  | 2.33 | 33 | 298420 | Ref. |  | Ref. |  |  |
| Q2 | 6 | 101 | 138472 | 1.55 [1.14, 2.11] | 0.006 | 1.49 [1.09, 2.04] | 0.012 | 6 | 38 | 293016 | 1.17 [0.74, 1.87] | 0.504 | 1.17 [0.73, 1.86] | 0.517 | 0.400 |
| Q3 | 9 | 102 | 146463 | 1.50 [1.10, 2.05] | 0.010 | 1.37 [1.01, 1.88] | 0.046 | 9 | 38 | 288936 | 1.21 [0.76, 1.93] | 0.428 | 1.15 [0.72, 1.83] | 0.566 | 0.540 |
| Q4 | 12 | 53 | 118233 | 0.98 [0.68, 1.40] | 0.899 | 0.89 [0.62, 1.28] | 0.543 | 12 | 24 | 225863 | 0.98 [0.58, 1.66] | 0.939 | 0.91 [0.54, 1.54] | 0.722 | 0.946 |
| Q5 | 17 | 85 | 144097 | 1.31 [0.95, 1.81] | 0.098 | 1.17 [0.85, 1.62] | 0.332 | 16.67 | 22 | 251687 | 0.83 [0.48, 1.42] | 0.487 | 0.77 [0.45, 1.33] | 0.346 | 0.193 |
| P for trend |  |  |  | 1.00 [0.98, 1.02] | 0.709 | 1.00 [0.98, 1.02] | 0.692 |  |  |  | 0.99 [0.95, 1.02] | 0.386 | 0.98 [0.95, 1.01] | 0.225 | 0.279 |
| per 1 SD |  |  |  | 1.03 [0.94, 1.13] | 0.533 | 0.99 [0.90, 1.10] | 0.912 |  |  |  | 0.96 [0.82, 1.14] | 0.653 | 0.94 [0.79, 1.11] | 0.439 | 0.607 |
|  | **aLCD** |  |  |  |  |  |  | **aLCD** |  |  |  |  |  |  |  |
| Q1 | 1 | 63 | 129972 | Ref. |  | Ref. |  | 1 | 26 | 295284 | Ref. |  | Ref. |  |  |
| Q2 | 4 | 80 | 126609 | 1.27 [0.91, 1.77] | 0.155 | 1.22 [0.88, 1.70] | 0.239 | 4 | 38 | 272964 | 1.57 [0.95, 2.58] | 0.077 | 1.47 [0.89, 2.43] | 0.131 | 0.543 |
| Q3 | 7 | 77 | 135698 | 1.14 [0.82, 1.59] | 0.446 | 1.03 [0.74, 1.44] | 0.854 | 7 | 35 | 273869 | 1.45 [0.87, 2.41] | 0.151 | 1.30 [0.78, 2.18] | 0.310 | 0.456 |
| Q4 | 10 | 87 | 145006 | 1.22 [0.88, 1.69] | 0.228 | 1.04 [0.75, 1.45] | 0.810 | 10 | 32 | 268452 | 1.36 [0.81, 2.29] | 0.239 | 1.15 [0.68, 1.95] | 0.598 | 0.751 |
| Q5 | 16 | 100 | 151863 | 1.37 [1.00, 1.87] | 0.053 | 1.15 [0.83, 1.58] | 0.402 | 15.67 | 24 | 247352 | 1.13 [0.65, 1.96] | 0.673 | 0.92 [0.53, 1.62] | 0.782 | 0.498 |
| P for trend |  |  |  | 1.02 [1.00, 1.03] | 0.096 | 1.00 [0.98, 1.02] | 0.695 |  |  |  | 1.00 [0.97, 1.03] | 0.983 | 0.99 [0.95, 1.02] | 0.383 | 0.629 |
| per 1 SD |  |  |  | 1.08 [0.99, 1.18] | 0.097 | 1.02 [0.93, 1.12] | 0.681 |  |  |  | 1.04 [0.89, 1.22] | 0.637 | 0.97 [0.82, 1.15] | 0.733 | 0.610 |
|  | **pLCD** |  |  |  |  |  |  | **pLCD** |  |  |  |  |  |  |  |
| Q1 | 11 | 63 | 129972 | Ref. |  | Ref. |  | 11 | 26 | 295284 | Ref. |  | Ref. |  |  |
| Q2 | 14 | 80 | 126609 | 0.89 [0.67, 1.19] | 0.438 | 0.91 [0.68, 1.21] | 0.519 | 14 | 38 | 272964 | 0.60 [0.37, 0.97] | 0.037 | 0.63 [0.39, 1.02] | 0.062 | 0.198 |
| Q3 | 16.5 | 77 | 135698 | 0.85 [0.63, 1.14] | 0.278 | 0.87 [0.65, 1.18] | 0.372 | 16.5 | 35 | 273869 | 0.74 [0.47, 1.17] | 0.194 | 0.80 [0.51, 1.27] | 0.342 | 0.763 |
| Q4 | 19 | 87 | 145006 | 0.94 [0.71, 1.25] | 0.670 | 0.97 [0.73, 1.30] | 0.852 | 19 | 32 | 268452 | 0.62 [0.39, 0.98] | 0.041 | 0.67 [0.42, 1.07] | 0.095 | 0.187 |
| Q5 | 22 | 100 | 151863 | 0.70 [0.50, 0.98] | 0.037 | 0.70 [0.50, 0.97] | 0.033 | 22 | 24 | 247352 | 0.54 [0.32, 0.89] | 0.017 | 0.55 [0.33, 0.93] | 0.025 | 0.442 |
| P for trend |  |  |  | 0.98 [0.95, 1.00] | 0.089 | 0.98 [0.95, 1.00] | 0.097 |  |  |  | 0.95 [0.91, 0.99] | 0.021 | 0.96 [0.92, 1.00] | 0.039 | 0.409 |
| per 1 SD |  |  |  | 0.90 [0.82, 1.00] | 0.041 | 0.90 [0.82, 1.00] | 0.040 |  |  |  | 0.81 [0.69, 0.95] | 0.011 | 0.83 [0.71, 0.97] | 0.021 | 0.391 |
|  | **PDI** |  |  |  |  |  |  | **PDI** |  |  |  |  |  |  |  |
| Q1 | 44 | 137 | 179837 | Ref. |  | Ref. |  | 44 | 26 | 259062 | Ref. |  | Ref. |  |  |
| Q2 | 48 | 97 | 149940 | 0.85 [0.65, 1.10] | 0.216 | 0.93 [0.72, 1.21] | 0.598 | 48 | 38 | 272758 | 1.39 [0.85, 2.30] | 0.193 | 1.56 [0.95, 2.58] | 0.081 | 0.072 |
| Q3 | 50.5 | 66 | 117834 | 0.74 [0.55, 0.99] | 0.041 | 0.85 [0.63, 1.14] | 0.284 | 50.5 | 33 | 250675 | 1.32 [0.79, 2.21] | 0.287 | 1.60 [0.95, 2.70] | 0.076 | 0.039 |
| Q4 | 53 | 57 | 126143 | 0.60 [0.44, 0.82] | 0.001 | 0.72 [0.52, 0.98] | 0.039 | 53 | 32 | 285474 | 1.14 [0.68, 1.91] | 0.630 | 1.44 [0.85, 2.45] | 0.173 | 0.027 |
| Q5 | 57 | 50 | 115393 | 0.58 [0.42, 0.80] | 0.001 | 0.72 [0.51, 1.00] | 0.051 | 57 | 26 | 289953 | 0.91 [0.53, 1.57] | 0.739 | 1.23 [0.70, 2.17] | 0.468 | 0.111 |
| P for trend |  |  |  | 0.95 [0.93, 0.98] | <0.001 | 0.97 [0.95, 0.99] | 0.014 |  |  |  | 0.99 [0.95, 1.02] | 0.508 | 1.01 [0.97, 1.05] | 0.546 | 0.076 |
| per 1 SD |  |  |  | 0.78 [0.71, 0.86] | <0.001 | 0.85 [0.77, 0.94] | 0.001 |  |  |  | 0.90 [0.77, 1.06] | 0.213 | 1.01 [0.85, 1.19] | 0.945 | 0.084 |
|  | **hPDI** |  |  |  |  |  |  | **hPDI** |  |  |  |  |  |  |  |
| Q1 | 49.5 | 143 | 194276 | Ref. |  | Ref. |  | 50 | 27 | 241196 | Ref. |  | Ref. |  |  |
| Q2 | 54 | 88 | 143251 | 0.81 [0.62, 1.05] | 0.112 | 0.78 [0.60, 1.02] | 0.074 | 54 | 34 | 245247 | 1.22 [0.74, 2.03] | 0.434 | 1.24 [0.74, 2.07] | 0.410 | 0.116 |
| Q3 | 57 | 88 | 138493 | 0.83 [0.63, 1.08] | 0.169 | 0.81 [0.62, 1.07] | 0.139 | 57 | 33 | 280852 | 1.04 [0.62, 1.73] | 0.883 | 1.06 [0.63, 1.78] | 0.833 | 0.369 |
| Q4 | 60 | 45 | 114151 | 0.51 [0.37, 0.72] | <0.001 | 0.51 [0.36, 0.71] | <0.001 | 60 | 31 | 281912 | 0.99 [0.59, 1.66] | 0.959 | 1.03 [0.60, 1.75] | 0.921 | 0.030 |
| Q5 | 64 | 43 | 98976 | 0.58 [0.41, 0.81] | 0.002 | 0.58 [0.41, 0.82] | 0.002 | 64 | 30 | 308714 | 0.89 [0.52, 1.50] | 0.654 | 0.95 [0.55, 1.64] | 0.851 | 0.135 |
| P for trend |  |  |  | 0.96 [0.94, 0.98] | <0.001 | 0.96 [0.93, 0.98] | <0.001 |  |  |  | 0.99 [0.95, 1.02] | 0.427 | 0.99 [0.96, 1.03] | 0.613 | 0.169 |
| per 1 SD |  |  |  | 0.81 [0.73, 0.89] | <0.001 | 0.80 [0.72, 0.89] | <0.001 |  |  |  | 0.91 [0.77, 1.07] | 0.248 | 0.92 [0.78, 1.10] | 0.371 | 0.175 |
|  | **uPDI** |  |  |  |  |  |  | **uPDI** |  |  |  |  |  |  |  |
| Q1 | 50 | 69 | 129859 | Ref. |  | Ref. |  | 50 | 29 | 303512 | Ref. |  | Ref. |  |  |
| Q2 | 54 | 62 | 125661 | 0.94 [0.66, 1.32] | 0.709 | 0.93 [0.66, 1.31] | 0.666 | 54 | 28 | 278025 | 1.07 [0.63, 1.79] | 0.805 | 1.04 [0.62, 1.76] | 0.873 | 0.726 |
| Q3 | 57 | 81 | 131458 | 1.20 [0.87, 1.65] | 0.275 | 1.16 [0.84, 1.61] | 0.357 | 57 | 35 | 266373 | 1.41 [0.86, 2.31] | 0.172 | 1.32 [0.80, 2.16] | 0.277 | 0.670 |
| Q4 | 60 | 84 | 143059 | 1.16 [0.84, 1.60] | 0.363 | 1.12 [0.81, 1.55] | 0.484 | 60 | 28 | 264874 | 1.15 [0.68, 1.94] | 0.591 | 1.06 [0.63, 1.79] | 0.833 | 0.861 |
| Q5 | 64 | 111 | 159109 | 1.46 [1.08, 1.98] | 0.014 | 1.38 [1.01, 1.88] | 0.041 | 64 | 35 | 245136 | 1.63 [0.99, 2.68] | 0.055 | 1.42 [0.85, 2.35] | 0.179 | 0.925 |
| P for trend |  |  |  | 1.03 [1.01, 1.05] | 0.005 | 1.03 [1.00, 1.05] | 0.016 |  |  |  | 1.03 [1.00, 1.07] | 0.061 | 1.02 [0.99, 1.06] | 0.210 | 0.649 |
| per 1 SD |  |  |  | 1.14 [1.04, 1.26] | 0.008 | 1.12 [1.01, 1.24] | 0.028 |  |  |  | 1.16 [0.99, 1.37] | 0.073 | 1.11 [0.93, 1.31] | 0.243 | 0.930 |

The nine dietary pattern scores were divided into quintiles and treated as categorical variables. Model 1 was adjusted for age at first completion of the Oxford WebQ and sex, and Model 2 was further adjusted for race, educational attainment, TDI, family history of cancer, smoking status, average alcohol intake (except aMED & SMD), MET, average energy intake, BMI, WHR and T2DM. The *p*-heterogeneity was obtained using the contrast method based on a fully unconstrained approach.

MAFLD, metabolic dysfunction-associated fatty liver disease; HR, hazard ratio; CI, confidence interval; aMED, alternate Mediterranean diet; PHD, Planetary Health Diet; SMD, sulfur microbial diet; LCD, Low-carbohydrate Diet; aLCD, animal based Low-carbohydrate Diet; pLCD, plant based Low-carbohydrate Diet; PDI, Plant-based Diet Index; hPDI, healthful Plant-based Diet Index; uPDI, unhealthful Plant-based Diet Index.

**Table S11. The associations of the scores of dietary patterns with the incidence of severe liver disease according to MAFLD status.**

|  | **MAFLD (59,473)** | | | | | | | **Non-MAFLD (117,211)** | | | | | | | ***p*-heterogeneity** |
| --- | --- | --- | --- | --- | --- | --- | --- | --- | --- | --- | --- | --- | --- | --- | --- |
|  | Median | Cases | Person-years | Model1  HR (95% CI) | Model1 *P* | Model2  HR (95% CI) | Model2 *P* | Median | Cases | Person-years | Model1 HR (95% CI) | Model1 *P* | Model2  HR (95% CI) | Model2 *P* |  |
|  | **aMED** |  |  |  |  |  |  | **aMED** |  |  |  |  |  |  |  |
| Q1 | 2 | 147 | 170455 | Ref. |  | Ref. |  | 2 | 57 | 242623 | Ref. |  | Ref. |  |  |
| Q2 | 3 | 126 | 186939 | 0.74 [0.59, 0.94] | 0.015 | 0.78 [0.61,0.99] | 0.038 | 3 | 57 | 325809 | 0.72 [0.50, 1.03] | 0.074 | 0.77 [0.53, 1.11] | 0.163 | 0.954 |
| Q3 | 4 | 98 | 174880 | 0.60 [0.47, 0.78] | <0.001 | 0.64 [0.50,0.83] | 0.001 | 4 | 48 | 364044 | 0.53 [0.36, 0.78] | 0.001 | 0.58 [0.39, 0.86] | 0.006 | 0.681 |
| Q4 | 5 | 69 | 103701 | 0.70 [0.53, 0.94] | 0.016 | 0.76 [0.57,1.02] | 0.068 | 5 | 39 | 257168 | 0.59 [0.39, 0.89] | 0.012 | 0.67 [0.44, 1.01] | 0.059 | 0.626 |
| Q5 | 6 | 21 | 52887 | 0.40 [0.26, 0.64] | <0.001 | 0.45 [0.28,0.71] | 0.001 | 6 | 24 | 167881 | 0.54 [0.33, 0.87] | 0.011 | 0.63 [0.38, 1.02] | 0.060 | 0.331 |
| P for trend |  |  |  | 0.84 [0.78, 0.91] | <0.001 | 0.86 [0.80,0.93] | <0.001 |  |  |  | 0.85 [0.76, 0.94] | 0.003 | 0.88 [0.79, 0.98] | 0.021 | 0.732 |
| per 1 SD |  |  |  | 0.84 [0.76, 0.92] | <0.001 | 0.86 [0.78,0.95] | 0.003 |  |  |  | 0.81 [0.71,0.93] | 0.002 | 0.85 [0.74, 0.98] | 0.022 | 0.894 |
|  | **PHD** |  |  |  |  |  |  | **PHD** |  |  |  |  |  |  |  |
| Q1 | 2 | 171 | 196045 | Ref. |  | Ref. |  | 2 | 61 | 291992 | Ref. |  | Ref. |  |  |
| Q2 | 3 | 102 | 173107 | 0.66 [0.52, 0.84] | 0.001 | 0.68 [0.53,0.87] | 0.002 | 3 | 47 | 309644 | 0.71 [0.49, 1.04] | 0.080 | 0.75 [0.51, 1.10] | 0.137 | 0.675 |
| Q3 | 3.5 | 49 | 92577 | 0.58 [0.42, 0.79] | 0.001 | 0.63 [0.46,0.86] | 0.004 | 3.5 | 31 | 204610 | 0.71 [0.46, 1.10] | 0.125 | 0.78 [0.51, 1.21] | 0.273 | 0.433 |
| Q4 | 4 | 77 | 119747 | 0.72 [0.55, 0.94] | 0.015 | 0.76 [0.58,1.00] | 0.051 | 4 | 49 | 264491 | 0.87 [0.60, 1.27] | 0.478 | 0.94 [0.65, 1.37] | 0.756 | 0.367 |
| Q5 | 5 | 62 | 107386 | 0.64 [0.48, 0.86] | 0.003 | 0.69 [0.52,0.93] | 0.014 | 5 | 37 | 286787 | 0.61 [0.40, 0.91] | 0.017 | 0.67 [0.44, 1.01] | 0.055 | 0.910 |
| P for trend |  |  |  | 0.85 [0.78, 0.93] | 0.001 | 0.88 [0.80,0.96] | 0.006 |  |  |  | 0.88 [0.77, 1.00] | 0.045 | 0.91 [0.80, 1.03] | 0.134 | 0.673 |
| per 1 SD |  |  |  | 0.85 [0.77,0.93] | 0.001 | 0.87 [0.80,0.96] | 0.006 |  |  |  | 0.85 [0.75,0.98] | 0.022 | 0.89 [0.78, 1.01] | 0.079 | 0.778 |
|  | **SMD** |  |  |  |  |  |  | **SMD** |  |  |  |  |  |  |  |
| Q1 | -2.6 | 87 | 136184 | Ref. |  | Ref. |  | -2.54 | 47 | 273879 | Ref. |  | Ref. |  |  |
| Q2 | -1.7 | 72 | 123145 | 0.92 [0.68, 1.26] | 0.615 | 0.95 [0.70,1.30] | 0.761 | -1.69 | 46 | 286299 | 0.94 [0.63, 1.41] | 0.766 | 0.97 [0.65, 1.47] | 0.898 | 0.936 |
| Q3 | -1.2 | 75 | 124429 | 0.96 [0.71, 1.31] | 0.813 | 0.99 [0.72,1.36] | 0.956 | -1.2 | 43 | 285452 | 0.90 [0.59, 1.36] | 0.608 | 0.92 [0.61, 1.41] | 0.709 | 0.785 |
| Q4 | -0.72 | 83 | 134824 | 1.00 [0.74, 1.35] | 0.986 | 1.01 [0.74,1.37] | 0.974 | -0.74 | 48 | 273994 | 1.06 [0.71, 1.59] | 0.763 | 1.06 [0.70, 1.61] | 0.774 | 0.855 |
| Q5 | 0.03 | 144 | 170280 | 1.43 [1.09, 1.86] | 0.009 | 1.31 [0.99,1.73] | 0.055 | -0.03 | 41 | 237901 | 1.08 [0.71, 1.65] | 0.715 | 1.02 [0.66, 1.58] | 0.917 | 0.344 |
| P for trend |  |  |  | 1.16 [1.05, 1.29] | 0.005 | 1.12 [1.01,1.25] | 0.037 |  |  |  | 1.04 [0.89, 1.22] | 0.619 | 1.02 [0.87, 1.20] | 0.824 | 0.342 |
| per 1 SD |  |  |  | 1.15 [1.06,1.26] | 0.001 | 1.11 [1.02,1.21] | 0.013 |  |  |  | 1.08 [0.93,1.24] | 0.309 | 1.06 [0.92, 1.22] | 0.439 | 0.584 |
|  | **LCD** |  |  |  |  |  |  | **LCD** |  |  |  |  |  |  |  |
| Q1 | 2.33 | 81 | 141783 | Ref. |  | Ref. |  | 2.33 | 51 | 298313 | Ref. |  | Ref. |  |  |
| Q2 | 6 | 105 | 138473 | 1.31 [0.98, 1.75] | 0.069 | 1.27 [0.95,1.69] | 0.112 | 6 | 58 | 292914 | 1.15 [0.79, 1.68] | 0.466 | 1.15 [0.79, 1.67] | 0.474 | 0.680 |
| Q3 | 9 | 115 | 146400 | 1.38 [1.04, 1.84] | 0.026 | 1.28 [0.96,1.70] | 0.095 | 9 | 51 | 288862 | 1.04 [0.71, 1.53] | 0.842 | 1.00 [0.68, 1.48] | 0.998 | 0.316 |
| Q4 | 12 | 65 | 118166 | 0.98 [0.71, 1.36] | 0.892 | 0.90 [0.65,1.25] | 0.526 | 12 | 31 | 225825 | 0.81 [0.52, 1.27] | 0.358 | 0.76 [0.48, 1.19] | 0.228 | 0.554 |
| Q5 | 17 | 95 | 144040 | 1.20 [0.89, 1.62] | 0.225 | 1.08 [0.80,1.45] | 0.634 | 16.67 | 34 | 251610 | 0.82 [0.53, 1.27] | 0.371 | 0.77 [0.49, 1.18] | 0.230 | 0.211 |
| P for trend |  |  |  | 1.00 [0.99, 1.02] | 0.715 | 1.00 [0.98,1.01] | 0.649 |  |  |  | 0.98 [0.96, 1.01] | 0.161 | 0.98 [0.95, 1.00] | 0.072 | 0.183 |
| per 1 SD |  |  |  | 1.03 [0.94,1.13] | 0.491 | 1.00 [0.91,1.09] | 0.917 |  |  |  | 0.92 [0.80,1.06] | 0.241 | 0.89 [0.78, 1.03] | 0.120 | 0.168 |
|  | **aLCD** |  |  |  |  |  |  | **aLCD** |  |  |  |  |  |  |  |
| Q1 | 1 | 76 | 129902 | Ref. |  | Ref. |  | 1 | 47 | 295175 | Ref. |  | Ref. |  |  |
| Q2 | 4 | 86 | 126577 | 1.13 [0.83, 1.54] | 0.445 | 1.09 [0.80,1.49] | 0.582 | 4 | 49 | 272904 | 1.11 [0.75, 1.66] | 0.604 | 1.05 [0.70, 1.57] | 0.799 | 0.886 |
| Q3 | 7 | 89 | 135649 | 1.09 [0.80, 1.47] | 0.600 | 1.00 [0.73,1.36] | 0.986 | 7 | 51 | 273766 | 1.16 [0.78, 1.72] | 0.468 | 1.06 [0.71, 1.59] | 0.758 | 0.823 |
| Q4 | 10 | 94 | 144957 | 1.09 [0.81, 1.48] | 0.571 | 0.95 [0.70,1.29] | 0.738 | 10 | 42 | 268408 | 0.98 [0.65, 1.49] | 0.932 | 0.86 [0.56, 1.31] | 0.475 | 0.709 |
| Q5 | 16 | 116 | 151776 | 1.31 [0.98, 1.76] | 0.064 | 1.12 [0.84,1.50] | 0.446 | 15.67 | 36 | 247272 | 0.93 [0.60, 1.43] | 0.733 | 0.79 [0.51, 1.22] | 0.283 | 0.191 |
| P for trend |  |  |  | 1.02 [1.00, 1.03] | 0.077 | 1.00 [0.99,1.02] | 0.597 |  |  |  | 0.99 [0.97, 1.02] | 0.557 | 0.98 [0.96, 1.01] | 0.158 | 0.179 |
| per 1 SD |  |  |  | 1.08 [0.99,1.17] | 0.091 | 1.02 [0.93,1.12] | 0.649 |  |  |  | 0.98 [0.86,1.13] | 0.797 | 0.93 [0.80, 1.07] | 0.290 | 0.294 |
|  | **pLCD** |  |  |  |  |  |  | **pLCD** |  |  |  |  |  |  |  |
| Q1 | 11 | 76 | 129902 | Ref. |  | Ref. |  | 11 | 47 | 295175 | Ref. |  | Ref. |  |  |
| Q2 | 14 | 86 | 126577 | 0.95 [0.73, 1.24] | 0.720 | 0.97 [0.74,1.26] | 0.795 | 14 | 49 | 272904 | 0.82 [0.56, 1.21] | 0.310 | 0.85 [0.58, 1.25] | 0.410 | 0.580 |
| Q3 | 16.5 | 89 | 135649 | 0.79 [0.59, 1.05] | 0.104 | 0.80 [0.60,1.07] | 0.139 | 16.5 | 51 | 273766 | 0.85 [0.58, 1.26] | 0.417 | 0.90 [0.61, 1.33] | 0.604 | 0.634 |
| Q4 | 19 | 94 | 144957 | 0.99 [0.76, 1.29] | 0.918 | 1.01 [0.77,1.32] | 0.956 | 19 | 42 | 268408 | 0.74 [0.50, 1.10] | 0.137 | 0.79 [0.53, 1.17] | 0.231 | 0.315 |
| Q5 | 22 | 116 | 151776 | 0.71 [0.52, 0.97] | 0.034 | 0.70 [0.51,0.95] | 0.024 | 22 | 36 | 247272 | 0.56 [0.36, 0.87] | 0.010 | 0.56 [0.36, 0.89] | 0.013 | 0.426 |
| P for trend |  |  |  | 0.98 [0.95, 1.00] | 0.080 | 0.98 [0.95,1.00] | 0.07 |  |  |  | 0.96 [0.92, 0.99] | 0.012 | 0.96 [0.93, 0.99] | 0.018 | 0.318 |
| per 1 SD |  |  |  | 0.90 [0.83,0.99] | 0.030 | 0.90 [0.82,0.99] | 0.023 |  |  |  | 0.83 [0.73,0.95] | 0.006 | 0.84 [0.74, 0.96] | 0.009 | 0.400 |
|  | **PDI** |  |  |  |  |  |  | **PDI** |  |  |  |  |  |  |  |
| Q1 | 44 | 142 | 179825 | Ref. |  | Ref. |  | 44 | 48 | 258942 | Ref. |  | Ref. |  |  |
| Q2 | 48 | 106 | 149860 | 0.89 [0.69, 1.15] | 0.382 | 0.97 [0.76,1.26] | 0.841 | 48 | 48 | 272701 | 0.94 [0.63, 1.41] | 0.771 | 1.03 [0.69, 1.54] | 0.895 | 0.804 |
| Q3 | 50.5 | 81 | 117762 | 0.87 [0.66, 1.14] | 0.323 | 1.00 [0.76,1.32] | 0.99 | 50.5 | 43 | 250608 | 0.92 [0.61, 1.39] | 0.684 | 1.06 [0.70, 1.61] | 0.778 | 0.819 |
| Q4 | 53 | 67 | 126090 | 0.68 [0.51, 0.91] | 0.009 | 0.80 [0.60,1.08] | 0.143 | 53 | 49 | 285375 | 0.93 [0.62, 1.38] | 0.707 | 1.11 [0.74, 1.66] | 0.624 | 0.199 |
| Q5 | 57 | 65 | 115326 | 0.73 [0.54, 0.97] | 0.033 | 0.88 [0.65,1.20] | 0.42 | 57 | 37 | 289900 | 0.69 [0.45, 1.06] | 0.090 | 0.86 [0.55, 1.35] | 0.521 | 0.934 |
| P for trend |  |  |  | 0.97 [0.95, 0.99] | 0.006 | 0.99 [0.97,1.01] | 0.231 |  |  |  | 0.98 [0.95, 1.01] | 0.111 | 0.99 [0.96, 1.02] | 0.681 | 1.000 |
| per 1 SD |  |  |  | 0.84 [0.77,0.92] | <0.001 | 0.90 [0.82,0.99] | 0.028 |  |  |  | 0.87 [0.76,0.99] | 0.040 | 0.94 [0.82, 1.08] | 0.411 | 0.610 |
|  | **hPDI** |  |  |  |  |  |  | **hPDI** |  |  |  |  |  |  |  |
| Q1 | 49.5 | 162 | 194186 | Ref. |  | Ref. |  | 50 | 42 | 241114 | Ref. |  | Ref. |  |  |
| Q2 | 54 | 95 | 143229 | 0.76 [0.59, 0.98] | 0.035 | 0.75 [0.58,0.97] | 0.027 | 54 | 50 | 245162 | 1.13 [0.75, 1.70] | 0.571 | 1.14 [0.75, 1.73] | 0.530 | 0.094 |
| Q3 | 57 | 102 | 138408 | 0.84 [0.66, 1.08] | 0.172 | 0.84 [0.65,1.08] | 0.171 | 57 | 42 | 280802 | 0.82 [0.53, 1.25] | 0.355 | 0.83 [0.54, 1.29] | 0.411 | 0.963 |
| Q4 | 60 | 51 | 114120 | 0.51 [0.37, 0.70] | <0.001 | 0.51 [0.37,0.70] | <0.001 | 60 | 47 | 281803 | 0.92 [0.60, 1.39] | 0.682 | 0.95 [0.62, 1.46] | 0.816 | 0.022 |
| Q5 | 64 | 51 | 98919 | 0.60 [0.44, 0.82] | 0.002 | 0.61 [0.44,0.84] | 0.003 | 64 | 44 | 308644 | 0.79 [0.51, 1.21] | 0.282 | 0.84 [0.54, 1.31] | 0.452 | 0.253 |
| P for trend |  |  |  | 0.96 [0.94, 0.98] | <0.001 | 0.96 [0.94,0.98] | <0.001 |  |  |  | 0.98 [0.95, 1.01] | 0.162 | 0.98 [0.96, 1.01] | 0.296 | 0.219 |
| per 1 SD |  |  |  | 0.82 [0.74,0.90] | <0.001 | 0.81 [0.74,0.90] | <0.001 |  |  |  | 0.87 [0.76,1.00] | 0.052 | 0.89 [0.77, 1.03] | 0.105 | 0.292 |
|  | **uPDI** |  |  |  |  |  |  | **uPDI** |  |  |  |  |  |  |  |
| Q1 | 50 | 82 | 129777 | Ref. |  | Ref. |  | 50 | 41 | 303444 | Ref. |  | Ref. |  |  |
| Q2 | 54 | 74 | 125609 | 0.94 [0.69, 1.29] | 0.709 | 0.94 [0.68,1.28] | 0.684 | 54 | 40 | 277967 | 1.09 [0.71, 1.69] | 0.695 | 1.07 [0.69, 1.66] | 0.751 | 0.639 |
| Q3 | 57 | 90 | 131416 | 1.12 [0.83, 1.52] | 0.441 | 1.10 [0.82,1.49] | 0.527 | 57 | 44 | 266295 | 1.28 [0.84, 1.96] | 0.254 | 1.22 [0.79, 1.87] | 0.369 | 0.699 |
| Q4 | 60 | 86 | 143042 | 1.01 [0.74, 1.36] | 0.961 | 0.99 [0.73,1.34] | 0.924 | 60 | 47 | 264796 | 1.42 [0.93, 2.16] | 0.103 | 1.33 [0.87, 2.03] | 0.188 | 0.267 |
| Q5 | 64 | 129 | 159019 | 1.46 [1.11, 1.93] | 0.008 | 1.40 [1.05,1.85] | 0.022 | 64 | 53 | 245022 | 1.86 [1.23, 2.81] | 0.003 | 1.66 [1.09, 2.53] | 0.017 | 0.511 |
| P for trend |  |  |  | 1.03 [1.01, 1.05] | 0.006 | 1.02 [1.00,1.04] | 0.016 |  |  |  | 1.05 [1.02, 1.08] | 0.002 | 1.04 [1.01, 1.07] | 0.010 | 0.275 |
| per 1 SD |  |  |  | 1.13 [1.03,1.24] | 0.008 | 1.11 [1.01,1.22] | 0.024 |  |  |  | 1.23 [1.08,1.42] | 0.003 | 1.19 [1.03, 1.36] | 0.016 | 0.410 |

The nine dietary pattern scores were divided into quintiles and treated as categorical variables. Model 1 was adjusted for age at first completion of the Oxford WebQ and sex, and Model 2 was further adjusted for race, educational attainment, TDI, family history of cancer, smoking status, average alcohol intake (except aMED & SMD), MET, average energy intake, BMI, WHR and T2DM. The *p*-heterogeneity was obtained using the contrast method based on a fully unconstrained approach.

MAFLD, metabolic dysfunction-associated fatty liver disease; HR, hazard ratio; CI, confidence interval; aMED, alternate Mediterranean diet; PHD, Planetary Health Diet; SMD, sulfur microbial diet; LCD, Low-carbohydrate Diet; aLCD, animal based Low-carbohydrate Diet; pLCD, plant based Low-carbohydrate Diet; PDI, Plant-based Diet Index; hPDI, healthful Plant-based Diet Index; uPDI, unhealthful Plant-based Diet Index.

**Table S12. The associations of the scores of dietary patterns with the incidence of liver cancer according to MAFLD status.**

|  | **MAFLD (59,** **473)** | | | | | | | **Non-MAFLD (117,211)** | | | | | | | ***p*-heterogeneity** |
| --- | --- | --- | --- | --- | --- | --- | --- | --- | --- | --- | --- | --- | --- | --- | --- |
|  | Median | Cases | Person-years | Model1  HR (95% CI) | Model1 *P* | Model2  HR (95% CI) | Model2 *P* | Median | Cases | Person-years | Model1 HR (95% CI) | Model1 *P* | Model2  HR (95% CI) | Model2 *P* |  |
|  | **aMED** |  |  |  |  |  |  | **aMED** |  |  |  |  |  |  |  |
| Q1 | 2 | 38 | 171019 | Ref. |  | Ref. |  | 2 | 25 | 242755 | Ref. |  | Ref. |  |  |
| Q2 | 3 | 37 | 187442 | 0.81 [0.52, 1.28] | 0.366 | 0.83 [0.53, 1.31] | 0.435 | 3 | 25 | 325993 | 0.69 [0.40, 1.21] | 0.197 | 0.70 [0.40, 1.23] | 0.214 | 0.643 |
| Q3 | 4 | 35 | 175181 | 0.79 [0.50, 1.25] | 0.310 | 0.82 [0.51, 1.30] | 0.393 | 4 | 27 | 364128 | 0.65 [0.37, 1.11] | 0.116 | 0.67 [0.39, 1.15] | 0.146 | 0.580 |
| Q4 | 5 | 27 | 103915 | 0.99 [0.60, 1.62] | 0.965 | 1.04 [0.63, 1.72] | 0.872 | 5 | 25 | 257221 | 0.81 [0.47, 1.41] | 0.460 | 0.85 [0.48, 1.49] | 0.563 | 0.601 |
| Q5 | 6 | 6 | 52964 | 0.41 [0.17, 0.96] | 0.041 | 0.43 [0.18, 1.02] | 0.057 | 6 | 12 | 167943 | 0.56 [0.28, 1.12] | 0.101 | 0.60 [0.30, 1.22] | 0.159 | 0.558 |
| P for trend |  |  |  | 0.91 [0.80, 1.05] | 0.189 | 0.93 [0.81, 1.06] | 0.280 |  |  |  | 0.91 [0.79, 1.06] | 0.218 | 0.93 [0.80, 1.08] | 0.327 | 1.000 |
| per 1 SD |  |  |  | 0.91 [0.77, 1.08] | 0.267 | 0.93 [0.78, 1.10] | 0.378 |  |  |  | 0.87 [0.72, 1.05] | 0.149 | 0.89 [0.74, 1.08] | 0.227 | 0.736 |
|  | **PHD** |  |  |  |  |  |  | **PHD** |  |  |  |  |  |  |  |
| Q1 | 2 | 45 | 196741 | Ref. |  | Ref. |  | 2 | 25 | 292157 | Ref. |  | Ref. |  |  |
| Q2 | 3 | 28 | 173483 | 0.67 [0.42, 1.07] | 0.095 | 0.69 [0.43, 1.10] | 0.117 | 3 | 27 | 309749 | 0.97 [0.56, 1.67] | 0.917 | 0.98 [0.57, 1.69] | 0.941 | 0.338 |
| Q3 | 3.5 | 21 | 92711 | 0.90 [0.54, 1.51] | 0.696 | 0.96 [0.57, 1.62] | 0.891 | 3.5 | 12 | 204700 | 0.66 [0.33, 1.31] | 0.235 | 0.68 [0.34, 1.35] | 0.267 | 0.435 |
| Q4 | 4 | 31 | 119962 | 1.06 [0.67, 1.67] | 0.817 | 1.12 [0.71, 1.78] | 0.626 | 4 | 24 | 264628 | 1.01 [0.57, 1.76] | 0.984 | 1.02 [0.58, 1.79] | 0.943 | 0.801 |
| Q5 | 5 | 18 | 107624 | 0.67 [0.39, 1.16] | 0.156 | 0.73 [0.42, 1.27] | 0.265 | 5 | 26 | 286807 | 0.99 [0.57, 1.73] | 0.984 | 1.01 [0.58, 1.76] | 0.970 | 0.417 |
| P for trend |  |  |  | 0.93 [0.79, 1.10] | 0.409 | 0.96 [0.82, 1.13] | 0.638 |  |  |  | 1.00 [0.84, 1.20] | 0.983 | 1.01 [0.84, 1.21] | 0.935 | 0.682 |
| per 1 SD |  |  |  | 0.91 [0.76, 1.07] | 0.254 | 0.93 [0.79, 1.11] | 0.437 |  |  |  | 0.99 [0.82, 1.19] | 0.874 | 0.99 [0.82, 1.19] | 0.912 | 0.627 |
|  | **SMD** |  |  |  |  |  |  | **SMD** |  |  |  |  |  |  |  |
| Q1 | -2.6 | 29 | 136486 | Ref. |  | Ref. |  | -2.54 | 26 | 273964 | Ref. |  | Ref. |  |  |
| Q2 | -1.7 | 24 | 123400 | 0.92 [0.53, 1.57] | 0.752 | 0.98 [0.57, 1.69] | 0.946 | -1.69 | 34 | 286384 | 1.25 [0.75, 2.08] | 0.392 | 1.25 [0.74, 2.09] | 0.401 | 0.525 |
| Q3 | -1.2 | 26 | 124646 | 1.00 [0.59, 1.70] | 0.993 | 1.09 [0.64, 1.87] | 0.751 | -1.2 | 21 | 285556 | 0.80 [0.45, 1.42] | 0.443 | 0.78 [0.44, 1.40] | 0.410 | 0.406 |
| Q4 | -0.72 | 31 | 135096 | 1.14 [0.69, 1.90] | 0.607 | 1.23 [0.73, 2.07] | 0.434 | -0.74 | 18 | 274125 | 0.74 [0.41, 1.35] | 0.328 | 0.70 [0.38, 1.29] | 0.250 | 0.169 |
| Q5 | 0.03 | 33 | 170893 | 1.03 [0.63, 1.70] | 0.900 | 1.02 [0.61, 1.71] | 0.945 | -0.03 | 15 | 238011 | 0.76 [0.40, 1.44] | 0.399 | 0.68 [0.35, 1.31] | 0.252 | 0.342 |
| P for trend |  |  |  | 1.04 [0.86, 1.25] | 0.696 | 1.03 [0.85, 1.25] | 0.750 |  |  |  | 0.85 [0.68, 1.06] | 0.146 | 0.82 [0.65, 1.03] | 0.081 | 0.137 |
| per 1 SD |  |  |  | 0.99 [0.85, 1.15] | 0.901 | 0.98 [0.84, 1.15] | 0.829 |  |  |  | 0.90 [0.74, 1.09] | 0.263 | 0.87 [0.71, 1.06] | 0.164 | 0.359 |
|  | **LCD** |  |  |  |  |  |  | **LCD** |  |  |  |  |  |  |  |
| Q1 | 2.33 | 20 | 142116 | Ref. |  | Ref. |  | 2.33 | 25 | 298463 | Ref. |  | Ref. |  |  |
| Q2 | 6 | 32 | 138854 | 1.59 [0.91, 2.78] | 0.104 | 1.55 [0.89, 2.71] | 0.124 | 6 | 27 | 293046 | 1.08 [0.63, 1.87] | 0.774 | 1.08 [0.63, 1.86] | 0.782 | 0.362 |
| Q3 | 9 | 31 | 146815 | 1.50 [0.86, 2.64] | 0.156 | 1.46 [0.83, 2.57] | 0.188 | 9 | 24 | 288971 | 0.99 [0.57, 1.74] | 0.979 | 0.97 [0.55, 1.70] | 0.909 | 0.316 |
| Q4 | 12 | 28 | 118343 | 1.70 [0.96, 3.02] | 0.070 | 1.61 [0.91, 2.87] | 0.104 | 12 | 21 | 225865 | 1.11 [0.62, 1.99] | 0.722 | 1.06 [0.59, 1.89] | 0.851 | 0.316 |
| Q5 | 17 | 32 | 144393 | 1.66 [0.95, 2.90] | 0.077 | 1.50 [0.85, 2.62] | 0.161 | 16.67 | 17 | 251696 | 0.83 [0.45, 1.55] | 0.567 | 0.77 [0.42, 1.44] | 0.416 | 0.117 |
| P for trend |  |  |  | 1.03 [0.99, 1.06] | 0.114 | 1.02 [0.99, 1.05] | 0.247 |  |  |  | 0.99 [0.95, 1.03] | 0.617 | 0.99 [0.95, 1.02] | 0.429 | 0.205 |
| per 1 SD |  |  |  | 1.10 [0.94, 1.29] | 0.220 | 1.07 [0.91, 1.25] | 0.437 |  |  |  | 0.92 [0.76, 1.12] | 0.431 | 0.90 [0.74, 1.09] | 0.281 | 0.176 |
|  | **aLCD** |  |  |  |  |  |  | **aLCD** |  |  |  |  |  |  |  |
| Q1 | 1 | 21 | 130194 | Ref. |  | Ref. |  | 1 | 24 | 295291 | Ref. |  | Ref. |  |  |
| Q2 | 4 | 28 | 126882 | 1.30 [0.74, 2.29] | 0.365 | 1.30 [0.74, 2.30] | 0.363 | 4 | 28 | 273015 | 1.23 [0.71, 2.12] | 0.458 | 1.19 [0.69, 2.06] | 0.529 | 0.826 |
| Q3 | 7 | 21 | 135986 | 0.91 [0.50, 1.66] | 0.751 | 0.89 [0.49, 1.64] | 0.713 | 7 | 18 | 273920 | 0.79 [0.43, 1.45] | 0.447 | 0.75 [0.40, 1.38] | 0.351 | 0.698 |
| Q4 | 10 | 39 | 145251 | 1.62 [0.95, 2.76] | 0.074 | 1.57 [0.92, 2.69] | 0.099 | 10 | 24 | 268462 | 1.09 [0.62, 1.91] | 0.775 | 0.99 [0.56, 1.76] | 0.980 | 0.249 |
| Q5 | 16 | 34 | 152207 | 1.39 [0.81, 2.39] | 0.237 | 1.32 [0.76, 2.28] | 0.327 | 15.67 | 20 | 247352 | 1.00 [0.55, 1.81] | 0.991 | 0.88 [0.48, 1.60] | 0.670 | 0.330 |
| P for trend |  |  |  | 1.02 [0.99, 1.05] | 0.167 | 1.02 [0.99, 1.05] | 0.266 |  |  |  | 1.00 [0.96, 1.03] | 0.836 | 0.99 [0.95, 1.02] | 0.510 | 0.205 |
| per 1 SD |  |  |  | 1.09 [0.94, 1.28] | 0.262 | 1.07 [0.91, 1.25] | 0.415 |  |  |  | 0.96 [0.79, 1.17] | 0.715 | 0.92 [0.75, 1.12] | 0.410 | 0.247 |
|  | **pLCD** |  |  |  |  |  |  | **pLCD** |  |  |  |  |  |  |  |
| Q1 | 11 | 21 | 130194 | Ref. |  | Ref. |  | 11 | 24 | 295291 | Ref. |  | Ref. |  |  |
| Q2 | 14 | 28 | 126882 | 0.85 [0.52, 1.41] | 0.534 | 0.83 [0.50, 1.37] | 0.470 | 14 | 28 | 273015 | 0.81 [0.46, 1.46] | 0.489 | 0.83 [0.46, 1.48] | 0.523 | 1.000 |
| Q3 | 16.5 | 21 | 135986 | 0.78 [0.46, 1.33] | 0.367 | 0.76 [0.45, 1.29] | 0.308 | 16.5 | 18 | 273920 | 0.90 [0.50, 1.59] | 0.705 | 0.92 [0.52, 1.64] | 0.790 | 0.631 |
| Q4 | 19 | 39 | 145251 | 0.98 [0.60, 1.60] | 0.932 | 0.92 [0.56, 1.51] | 0.751 | 19 | 24 | 268462 | 0.99 [0.58, 1.71] | 0.974 | 1.03 [0.59, 1.78] | 0.921 | 0.766 |
| Q5 | 22 | 34 | 152207 | 1.14 [0.69, 1.88] | 0.605 | 1.05 [0.63, 1.74] | 0.849 | 22 | 20 | 247352 | 0.83 [0.46, 1.51] | 0.543 | 0.84 [0.46, 1.54] | 0.578 | 0.580 |
| P for trend |  |  |  | 1.01 [0.97, 1.06] | 0.559 | 1.01 [0.96, 1.05] | 0.803 |  |  |  | 0.99 [0.95, 1.04] | 0.790 | 1.00 [0.95, 1.05] | 0.854 | 0.772 |
| per 1 SD |  |  |  | 1.03 [0.87, 1.21] | 0.752 | 1.00 [0.85, 1.18] | 0.986 |  |  |  | 0.94 [0.78, 1.14] | 0.528 | 0.95 [0.79, 1.14] | 0.571 | 0.683 |
|  | **PDI** |  |  |  |  |  |  | **PDI** |  |  |  |  |  |  |  |
| Q1 | 44 | 45 | 180323 | Ref. |  | Ref. |  | 44 | 23 | 259030 | Ref. |  | Ref. |  |  |
| Q2 | 48 | 30 | 150228 | 0.78 [0.49, 1.24] | 0.292 | 0.79 [0.50, 1.26] | 0.327 | 48 | 21 | 272831 | 0.84 [0.47, 1.52] | 0.572 | 0.88 [0.49, 1.60] | 0.686 | 0.778 |
| Q3 | 50.5 | 14 | 118104 | 0.46 [0.25, 0.84] | 0.012 | 0.47 [0.26, 0.87] | 0.015 | 50.5 | 23 | 250704 | 1.00 [0.56, 1.78] | 0.992 | 1.08 [0.60, 1.93] | 0.800 | 0.052 |
| Q4 | 53 | 29 | 126301 | 0.90 [0.56, 1.44] | 0.656 | 0.92 [0.57, 1.48] | 0.723 | 53 | 18 | 285543 | 0.69 [0.37, 1.28] | 0.241 | 0.76 [0.40, 1.42] | 0.383 | 0.637 |
| Q5 | 57 | 25 | 115564 | 0.85 [0.52, 1.39] | 0.523 | 0.85 [0.51, 1.41] | 0.523 | 57 | 29 | 289932 | 1.09 [0.63, 1.89] | 0.752 | 1.23 [0.69, 2.17] | 0.479 | 0.344 |
| P for trend |  |  |  | 0.99 [0.95, 1.02] | 0.494 | 0.99 [0.95, 1.03] | 0.510 |  |  |  | 1.00 [0.96, 1.05] | 0.871 | 1.01 [0.97, 1.06] | 0.581 | 0.514 |
| per 1 SD |  |  |  | 0.96 [0.81, 1.13] | 0.604 | 0.96 [0.81, 1.13] | 0.630 |  |  |  | 1.00 [0.83, 1.21] | 0.980 | 1.04 [0.85, 1.27] | 0.708 | 0.548 |
|  | **hPDI** |  |  |  |  |  |  | **hPDI** |  |  |  |  |  |  |  |
| Q1 | 49.5 | 49 | 194766 | Ref. |  | Ref. |  | 50 | 20 | 241210 | Ref. |  | Ref. |  |  |
| Q2 | 54 | 27 | 143622 | 0.68 [0.43, 1.09] | 0.113 | 0.72 [0.45, 1.15] | 0.168 | 54 | 18 | 245319 | 0.81 [0.43, 1.53] | 0.518 | 0.81 [0.43, 1.54] | 0.520 | 0.771 |
| Q3 | 57 | 24 | 138804 | 0.62 [0.38, 1.01] | 0.053 | 0.67 [0.40, 1.10] | 0.110 | 57 | 27 | 280888 | 1.02 [0.57, 1.83] | 0.942 | 1.03 [0.57, 1.86] | 0.921 | 0.279 |
| Q4 | 60 | 26 | 114222 | 0.80 [0.50, 1.29] | 0.364 | 0.88 [0.54, 1.44] | 0.618 | 60 | 22 | 281909 | 0.83 [0.45, 1.53] | 0.547 | 0.84 [0.45, 1.56] | 0.580 | 0.908 |
| Q5 | 64 | 17 | 99108 | 0.62 [0.35, 1.08] | 0.090 | 0.69 [0.39, 1.23] | 0.207 | 64 | 27 | 308715 | 0.93 [0.52, 1.67] | 0.807 | 0.96 [0.53, 1.76] | 0.899 | 0.436 |
| P for trend |  |  |  | 0.97 [0.94, 1.01] | 0.095 | 0.98 [0.94, 1.02] | 0.259 |  |  |  | 1.00 [0.96, 1.04] | 0.861 | 1.00 [0.96, 1.04] | 0.964 | 0.489 |
| per 1 SD |  |  |  | 0.86 [0.72, 1.02] | 0.084 | 0.90 [0.75, 1.08] | 0.258 |  |  |  | 0.98 [0.81, 1.19] | 0.828 | 0.99 [0.81, 1.21] | 0.937 | 0.491 |
|  | **uPDI** |  |  |  |  |  |  | **uPDI** |  |  |  |  |  |  |  |
| Q1 | 50 | 27 | 130067 | Ref. |  | Ref. |  | 50 | 20 | 303520 | Ref. |  | Ref. |  |  |
| Q2 | 54 | 26 | 125849 | 1.02 [0.59, 1.75] | 0.947 | 1.05 [0.61, 1.81] | 0.850 | 54 | 22 | 278026 | 1.26 [0.69, 2.31] | 0.450 | 1.25 [0.68, 2.30] | 0.466 | 0.676 |
| Q3 | 57 | 30 | 131723 | 1.18 [0.70, 1.99] | 0.527 | 1.24 [0.73, 2.09] | 0.422 | 57 | 23 | 266457 | 1.44 [0.79, 2.62] | 0.235 | 1.41 [0.77, 2.59] | 0.261 | 0.754 |
| Q4 | 60 | 27 | 143319 | 1.01 [0.59, 1.73] | 0.967 | 1.07 [0.62, 1.83] | 0.811 | 60 | 26 | 264874 | 1.74 [0.97, 3.11] | 0.065 | 1.70 [0.94, 3.07] | 0.077 | 0.258 |
| Q5 | 64 | 33 | 159564 | 1.26 [0.76, 2.11] | 0.370 | 1.32 [0.79, 2.22] | 0.291 | 64 | 23 | 245163 | 1.89 [1.03, 3.46] | 0.039 | 1.82 [0.99, 3.37] | 0.055 | 0.432 |
| P for trend |  |  |  | 1.01 [0.98, 1.05] | 0.412 | 1.02 [0.98, 1.05] | 0.321 |  |  |  | 1.05 [1.01, 1.09] | 0.019 | 1.05 [1.00, 1.09] | 0.029 | 0.303 |
| per 1 SD |  |  |  | 1.07 [0.91, 1.27] | 0.401 | 1.09 [0.92, 1.29] | 0.305 |  |  |  | 1.24 [1.02, 1.51] | 0.027 | 1.23 [1.01, 1.49] | 0.041 | 0.358 |

The nine dietary pattern scores were divided into quintiles and treated as categorical variables. Model 1 was adjusted for age at first completion of the Oxford WebQ and sex, and Model 2 was further adjusted for race, educational attainment, TDI, family history of cancer, smoking status, average alcohol intake (except aMED & SMD), MET, average energy intake, BMI, WHR and T2DM. The *p*-heterogeneity was obtained using the contrast method based on a fully unconstrained approach.

MAFLD, metabolic dysfunction-associated fatty liver disease; HR, hazard ratio; CI, confidence interval; aMED, alternate Mediterranean diet; PHD, Planetary Health Diet; SMD, sulfur microbial diet; LCD, Low-carbohydrate Diet; aLCD, animal based Low-carbohydrate Diet; pLCD, plant based Low-carbohydrate Diet; PDI, Plant-based Diet Index; hPDI, healthful Plant-based Diet Index; uPDI, unhealthful Plant-based Diet Index.

**Table S13. The associations of the scores of dietary patterns with the incidence of hepatocellular carcinoma according to MAFLD status.**

|  | **MAFLD (59,392)** | | | | | | | **Non-MAFLD (117,128)** | | | | | | | ***p*-heterogeneity** |
| --- | --- | --- | --- | --- | --- | --- | --- | --- | --- | --- | --- | --- | --- | --- | --- |
|  | Median | Cases | Person-years | Model1  HR (95% CI) | Model1 *P* | Model2  HR (95% CI) | Model2 *P* | Median | Cases | Person-years | Model1 HR (95% CI) | Model1 *P* | Model2  HR (95% CI) | Model2 *P* |  |
|  | **aMED** |  |  |  |  |  |  | **aMED** |  |  |  |  |  |  |  |
| Q1 | 2 | 17 | 170887 | Ref. |  | Ref. |  | 2 | 7 | 242647 | Ref. |  | Ref. |  |  |
| Q2 | 3 | 18 | 187322 | 0.86 [0.44, 1.67] | 0.651 | 0.91 [0.47, 1.76] | 0.769 | 3 | 6 | 325859 | 0.59 [0.20, 1.76] | 0.347 | 0.63 [0.21, 1.87] | 0.402 | 0.573 |
| Q3 | 4 | 13 | 175057 | 0.63 [0.31, 1.30] | 0.215 | 0.66 [0.32, 1.36] | 0.261 | 4 | 5 | 363998 | 0.42 [0.13, 1.34] | 0.144 | 0.47 [0.15, 1.48] | 0.194 | 0.623 |
| Q4 | 5 | 11 | 103833 | 0.86 [0.40, 1.85] | 0.708 | 0.91 [0.42, 1.97] | 0.820 | 5 | 9 | 257131 | 1.03 [0.38, 2.78] | 0.946 | 1.19 [0.44, 3.26] | 0.729 | 0.678 |
| Q5 | 6 | 3 | 52947 | 0.43 [0.13, 1.46] | 0.177 | 0.44 [0.13, 1.53] | 0.197 | 6 | 4 | 167892 | 0.66 [0.19, 2.28] | 0.515 | 0.82 [0.23, 2.90] | 0.761 | 0.490 |
| P for trend |  |  |  | 0.87 [0.71, 1.07] | 0.197 | 0.88 [0.71, 1.09] | 0.241 |  |  |  | 0.99 [0.75, 1.30] | 0.934 | 1.04 [0.78, 1.38] | 0.788 | 0.359 |
| per 1 SD |  |  |  | 0.88 [0.68, 1.14] | 0.324 | 0.89 [0.69, 1.15] | 0.375 |  |  |  | 0.95 [0.67, 1.36] | 0.792 | 1.02 [0.71, 1.46] | 0.931 | 0.545 |
|  | **PHD** |  |  |  |  |  |  | **PHD** |  |  |  |  |  |  |  |
| Q1 | 2 | 22 | 196600 | Ref. |  | Ref. |  | 2 | 5 | 292021 | Ref. |  | Ref. |  |  |
| Q2 | 3 | 12 | 173384 | 0.58 [0.29, 1.18] | 0.131 | 0.61 [0.30, 1.23] | 0.169 | 3 | 7 | 309632 | 1.25 [0.40, 3.93] | 0.706 | 1.29 [0.41, 4.06] | 0.668 | 0.276 |
| Q3 | 3.5 | 7 | 92624 | 0.61 [0.26, 1.43] | 0.255 | 0.69 [0.29, 1.62] | 0.393 | 3.5 | 3 | 204654 | 0.81 [0.19, 3.39] | 0.773 | 0.86 [0.20, 3.60] | 0.834 | 0.797 |
| Q4 | 4 | 14 | 119875 | 0.98 [0.50, 1.91] | 0.942 | 1.08 [0.55, 2.12] | 0.821 | 4 | 7 | 264498 | 1.44 [0.46, 4.54] | 0.535 | 1.55 [0.49, 4.92] | 0.453 | 0.596 |
| Q5 | 5 | 7 | 107562 | 0.54 [0.23, 1.25] | 0.150 | 0.61 [0.26, 1.43] | 0.252 | 5 | 9 | 286723 | 1.67 [0.56, 5.01] | 0.357 | 1.85 [0.62, 5.57] | 0.273 | 0.118 |
| P for trend |  |  |  | 0.87 [0.67, 1.11] | 0.266 | 0.91 [0.71, 1.17] | 0.457 |  |  |  | 1.19 [0.84, 1.69] | 0.317 | 1.24 [0.87, 1.75] | 0.233 | 0.158 |
| per 1 SD |  |  |  | 0.81 [0.62, 1.05] | 0.113 | 0.85 [0.65, 1.10] | 0.223 |  |  |  | 1.14 [0.80, 1.62] | 0.461 | 1.18 [0.83, 1.68] | 0.350 | 0.144 |
|  | **SMD** |  |  |  |  |  |  | **SMD** |  |  |  |  |  |  |  |
| Q1 | -2.6 | 11 | 136401 | Ref. |  | Ref. |  | -2.54 | 6 | 273827 | Ref. |  | Ref. |  |  |
| Q2 | -1.7 | 13 | 123324 | 1.31 [0.59, 2.93] | 0.506 | 1.53 [0.68, 3.45] | 0.302 | -1.69 | 8 | 286204 | 1.26 [0.44, 3.64] | 0.667 | 1.28 [0.44, 3.73] | 0.645 | 0.795 |
| Q3 | -1.2 | 11 | 124557 | 1.14 [0.49, 2.62] | 0.763 | 1.38 [0.59, 3.22] | 0.462 | -1.2 | 5 | 285478 | 0.81 [0.25, 2.67] | 0.734 | 0.82 [0.24, 2.73] | 0.741 | 0.491 |
| Q4 | -0.72 | 16 | 135006 | 1.59 [0.74, 3.43] | 0.235 | 1.89 [0.86, 4.18] | 0.114 | -0.74 | 5 | 274052 | 0.89 [0.27, 2.91] | 0.842 | 0.84 [0.25, 2.84] | 0.778 | 0.273 |
| Q5 | 0.03 | 11 | 170757 | 0.94 [0.41, 2.17] | 0.883 | 0.94 [0.40, 2.23] | 0.891 | -0.03 | 7 | 237967 | 1.54 [0.52, 4.60] | 0.437 | 1.36 [0.44, 4.22] | 0.593 | 0.610 |
| P for trend |  |  |  | 1.01 [0.77, 1.34] | 0.933 | 1.01 [0.76, 1.34] | 0.941 |  |  |  | 1.10 [0.72, 1.68] | 0.655 | 1.05 [0.68, 1.62] | 0.824 | 0.883 |
| per 1 SD |  |  |  | 0.97 [0.77, 1.22] | 0.791 | 0.98 [0.78, 1.21] | 0.823 |  |  |  | 1.07 [0.73, 1.57] | 0.742 | 1.02 [0.69, 1.50] | 0.924 | 0.861 |
|  | **LCD** |  |  |  |  |  |  | **LCD** |  |  |  |  |  |  |  |
| Q1 | 2.33 | 11 | 142081 | Ref. |  | Ref. |  | 2.33 | 7 | 298343 | Ref. |  | Ref. |  |  |
| Q2 | 6 | 12 | 138734 | 1.08 [0.48, 2.46] | 0.847 | 1.05 [0.46, 2.39] | 0.900 | 6 | 10 | 292940 | 1.42 [0.54, 3.74] | 0.475 | 1.38 [0.52, 3.62] | 0.515 | 0.674 |
| Q3 | 9 | 13 | 146713 | 1.17 [0.53, 2.62] | 0.697 | 1.12 [0.50, 2.51] | 0.782 | 9 | 5 | 288860 | 0.73 [0.23, 2.29] | 0.585 | 0.67 [0.21, 2.12] | 0.497 | 0.475 |
| Q4 | 12 | 7 | 118212 | 0.80 [0.31, 2.06] | 0.641 | 0.72 [0.28, 1.86] | 0.498 | 12 | 2 | 225748 | 0.37 [0.08, 1.78] | 0.215 | 0.33 [0.07, 1.59] | 0.168 | 0.402 |
| Q5 | 17 | 19 | 144306 | 1.89 [0.90, 3.97] | 0.094 | 1.55 [0.73, 3.27] | 0.255 | 16.67 | 7 | 251637 | 1.19 [0.42, 3.40] | 0.747 | 1.02 [0.36, 2.93] | 0.970 | 0.525 |
| P for trend |  |  |  | 1.04 [0.99, 1.09] | 0.112 | 1.03 [0.98, 1.08] | 0.310 |  |  |  | 0.99 [0.92, 1.06] | 0.710 | 0.98 [0.91, 1.05] | 0.506 | 0.259 |
| per 1 SD |  |  |  | 1.19 [0.94, 1.51] | 0.145 | 1.11 [0.87, 1.40] | 0.402 |  |  |  | 0.92 [0.64, 1.34] | 0.679 | 0.87 [0.60, 1.27] | 0.476 | 0.282 |
|  | **aLCD** |  |  |  |  |  |  | **aLCD** |  |  |  |  |  |  |  |
| Q1 | 1 | 10 | 130152 | Ref. |  | Ref. |  | 1 | 9 | 295206 | Ref. |  | Ref. |  |  |
| Q2 | 4 | 12 | 126779 | 1.15 [0.50, 2.67] | 0.742 | 1.19 [0.51, 2.76] | 0.691 | 4 | 7 | 272864 | 0.82 [0.30, 2.19] | 0.687 | 0.74 [0.27, 2.00] | 0.554 | 0.477 |
| Q3 | 7 | 9 | 135907 | 0.81 [0.33, 1.98] | 0.637 | 0.79 [0.32, 1.95] | 0.604 | 7 | 4 | 273843 | 0.46 [0.14, 1.50] | 0.200 | 0.41 [0.12, 1.33] | 0.136 | 0.393 |
| Q4 | 10 | 13 | 145109 | 1.14 [0.50, 2.60] | 0.754 | 1.07 [0.47, 2.48] | 0.866 | 10 | 3 | 268332 | 0.36 [0.10, 1.32] | 0.122 | 0.30 [0.08, 1.11] | 0.071 | 0.109 |
| Q5 | 16 | 18 | 152100 | 1.58 [0.73, 3.42] | 0.248 | 1.41 [0.64, 3.09] | 0.392 | 15.67 | 8 | 247283 | 1.04 [0.40, 2.70] | 0.938 | 0.82 [0.31, 2.18] | 0.695 | 0.397 |
| P for trend |  |  |  | 1.03 [0.98, 1.08] | 0.192 | 1.02 [0.97, 1.07] | 0.357 |  |  |  | 0.99 [0.93, 1.07] | 0.864 | 0.98 [0.91, 1.05] | 0.577 | 0.366 |
| per 1 SD |  |  |  | 1.15 [0.91, 1.45] | 0.235 | 1.09 [0.86, 1.38] | 0.462 |  |  |  | 0.90 [0.61, 1.32] | 0.587 | 0.83 [0.56, 1.23] | 0.348 | 0.245 |
|  | **pLCD** |  |  |  |  |  |  | **pLCD** |  |  |  |  |  |  |  |
| Q1 | 11 | 10 | 130152 | Ref. |  | Ref. |  | 11 | 9 | 295206 | Ref. |  | Ref. |  |  |
| Q2 | 14 | 12 | 126779 | 0.71 [0.33, 1.53] | 0.386 | 0.68 [0.31, 1.46] | 0.323 | 14 | 7 | 272864 | 2.43 [0.47, 12.53] | 0.288 | 2.45 [0.47, 12.62] | 0.285 | 0.167 |
| Q3 | 16.5 | 9 | 135907 | 0.58 [0.25, 1.36] | 0.208 | 0.54 [0.23, 1.27] | 0.161 | 16.5 | 4 | 273843 | 4.59 [0.99, 21.25] | 0.051 | 4.79 [1.03, 22.24] | 0.045 | 0.015 |
| Q4 | 19 | 13 | 145109 | 1.06 [0.52, 2.14] | 0.875 | 0.94 [0.47, 1.92] | 0.873 | 19 | 3 | 268332 | 5.07 [1.12, 22.85] | 0.035 | 5.28 [1.16, 23.93] | 0.031 | 0.043 |
| Q5 | 22 | 18 | 152100 | 1.06 [0.50, 2.24] | 0.884 | 0.89 [0.42, 1.90] | 0.772 | 22 | 8 | 247283 | 2.18 [0.40, 11.90] | 0.369 | 2.23 [0.40, 12.24] | 0.358 | 0.336 |
| P for trend |  |  |  | 1.02 [0.95, 1.08] | 0.656 | 1.00 [0.94, 1.07] | 0.996 |  |  |  | 1.07 [0.97, 1.17] | 0.186 | 1.07 [0.97, 1.18] | 0.174 | 0.259 |
| per 1 SD |  |  |  | 1.02 [0.79, 1.31] | 0.879 | 0.96 [0.75, 1.24] | 0.777 |  |  |  | 1.37 [0.95, 1.96] | 0.088 | 1.37 [0.96, 1.96] | 0.085 | 0.110 |
|  | **PDI** |  |  |  |  |  |  | **PDI** |  |  |  |  |  |  |  |
| Q1 | 44 | 21 | 180174 | Ref. |  | Ref. |  | 44 | 8 | 258938 | Ref. |  | Ref. |  |  |
| Q2 | 48 | 11 | 150132 | 0.61 [0.29, 1.27] | 0.185 | 0.63 [0.30, 1.32] | 0.220 | 48 | 7 | 272754 | 0.80 [0.29, 2.20] | 0.663 | 0.86 [0.31, 2.38] | 0.767 | 0.628 |
| Q3 | 50.5 | 8 | 118065 | 0.57 [0.25, 1.28] | 0.174 | 0.59 [0.26, 1.33] | 0.203 | 50.5 | 4 | 250595 | 0.49 [0.15, 1.62] | 0.242 | 0.54 [0.16, 1.83] | 0.325 | 0.906 |
| Q4 | 53 | 10 | 126192 | 0.67 [0.32, 1.43] | 0.306 | 0.70 [0.33, 1.50] | 0.359 | 53 | 3 | 285437 | 0.32 [0.09, 1.22] | 0.096 | 0.37 [0.10, 1.43] | 0.150 | 0.414 |
| Q5 | 57 | 12 | 115483 | 0.90 [0.44, 1.82] | 0.760 | 0.84 [0.40, 1.76] | 0.643 | 57 | 9 | 289803 | 0.95 [0.37, 2.46] | 0.913 | 1.13 [0.41, 3.07] | 0.813 | 0.642 |
| P for trend |  |  |  | 0.99 [0.93, 1.04] | 0.624 | 0.98 [0.93, 1.04] | 0.555 |  |  |  | 0.98 [0.91, 1.06] | 0.649 | 0.99 [0.92, 1.08] | 0.901 | 0.839 |
| per 1 SD |  |  |  | 0.93 [0.72, 1.19] | 0.551 | 0.92 [0.72, 1.18] | 0.499 |  |  |  | 0.85 [0.59, 1.23] | 0.391 | 0.90 [0.62, 1.32] | 0.600 | 0.924 |
|  | **hPDI** |  |  |  |  |  |  | **hPDI** |  |  |  |  |  |  |  |
| Q1 | 49.5 | 26 | 194624 | Ref. |  | Ref. |  | 50 | 4 | 241112 | Ref. |  | Ref. |  |  |
| Q2 | 54 | 12 | 143534 | 0.57 [0.29, 1.12] | 0.103 | 0.63 [0.31, 1.26] | 0.189 | 54 | 3 | 245216 | 0.66 [0.15, 2.97] | 0.593 | 0.69 [0.15, 3.09] | 0.623 | 0.915 |
| Q3 | 57 | 9 | 138716 | 0.44 [0.20, 0.93] | 0.032 | 0.52 [0.24, 1.12] | 0.095 | 57 | 8 | 280766 | 1.47 [0.44, 4.92] | 0.529 | 1.57 [0.46, 5.31] | 0.468 | 0.134 |
| Q4 | 60 | 6 | 114108 | 0.35 [0.15, 0.86] | 0.022 | 0.43 [0.18, 1.07] | 0.071 | 60 | 8 | 281837 | 1.45 [0.43, 4.86] | 0.548 | 1.54 [0.45, 5.29] | 0.489 | 0.100 |
| Q5 | 64 | 9 | 99064 | 0.64 [0.30, 1.37] | 0.253 | 0.80 [0.36, 1.75] | 0.570 | 64 | 8 | 308597 | 1.31 [0.39, 4.41] | 0.665 | 1.49 [0.43, 5.16] | 0.533 | 0.408 |
| P for trend |  |  |  | 0.94 [0.89, 1.00] | 0.042 | 0.96 [0.91, 1.02] | 0.185 |  |  |  | 1.03 [0.96, 1.11] | 0.426 | 1.04 [0.96, 1.13] | 0.323 | 0.115 |
| per 1 SD |  |  |  | 0.77 [0.59, 1.00] | 0.047 | 0.85 [0.65, 1.12] | 0.254 |  |  |  | 1.04 [0.72, 1.50] | 0.849 | 1.08 [0.73, 1.58] | 0.704 | 0.320 |
|  | **uPDI** |  |  |  |  |  |  | **uPDI** |  |  |  |  |  |  |  |
| Q1 | 50 | 15 | 129998 | Ref. |  | Ref. |  | 50 | 8 | 303461 | Ref. |  | Ref. |  |  |
| Q2 | 54 | 10 | 125770 | 0.70 [0.32, 1.56] | 0.386 | 0.76 [0.34, 1.69] | 0.500 | 54 | 5 | 277919 | 0.72 [0.24, 2.21] | 0.569 | 0.70 [0.23, 2.16] | 0.538 | 0.907 |
| Q3 | 57 | 11 | 131606 | 0.78 [0.36, 1.71] | 0.541 | 0.88 [0.40, 1.92] | 0.745 | 57 | 4 | 266319 | 0.63 [0.19, 2.11] | 0.457 | 0.62 [0.18, 2.06] | 0.433 | 0.636 |
| Q4 | 60 | 10 | 143197 | 0.68 [0.31, 1.51] | 0.344 | 0.78 [0.35, 1.75] | 0.540 | 60 | 9 | 264758 | 1.53 [0.59, 3.98] | 0.383 | 1.49 [0.57, 3.94] | 0.416 | 0.313 |
| Q5 | 64 | 16 | 159474 | 1.15 [0.57, 2.34] | 0.695 | 1.29 [0.63, 2.64] | 0.493 | 64 | 5 | 245070 | 1.05 [0.34, 3.25] | 0.927 | 0.99 [0.32, 3.10] | 0.987 | 0.699 |
| P for trend |  |  |  | 1.01 [0.96, 1.06] | 0.745 | 1.02 [0.97, 1.07] | 0.523 |  |  |  | 1.02 [0.95, 1.10] | 0.544 | 1.02 [0.95, 1.10] | 0.612 | 1.000 |
| per 1 SD |  |  |  | 1.03 [0.80, 1.33] | 0.819 | 1.08 [0.84, 1.38] | 0.567 |  |  |  | 1.13 [0.78, 1.63] | 0.525 | 1.11 [0.76, 1.62] | 0.584 | 0.906 |

The nine dietary pattern scores were divided into quintiles and treated as categorical variables. Model 1 was adjusted for age at first completion of the Oxford WebQ and sex, and Model 2 was further adjusted for race, educational attainment, TDI, family history of cancer, smoking status, average alcohol intake (except aMED & SMD), MET, average energy intake, BMI, WHR and T2DM. The *p*-heterogeneity was obtained using the contrast method based on a fully unconstrained approach.

MAFLD, metabolic dysfunction-associated fatty liver disease; HR, hazard ratio; CI, confidence interval; aMED, alternate Mediterranean diet; PHD, Planetary Health Diet; SMD, sulfur microbial diet; LCD, Low-carbohydrate Diet; aLCD, animal based Low-carbohydrate Diet; pLCD, plant based Low-carbohydrate Diet; PDI, Plant-based Diet Index; hPDI, healthful Plant-based Diet Index; uPDI, unhealthful Plant-based Diet Index.

**Table S14. The associations of the scores of dietary patterns with the incidence of intrahepatic cholangiocarcinoma according to MAFLD status.**

|  | **MAFLD (59,392)** | | | | | | | **Non-MAFLD (117,164)** | | | | | | | ***p*-heterogeneity** |
| --- | --- | --- | --- | --- | --- | --- | --- | --- | --- | --- | --- | --- | --- | --- | --- |
|  | Median | Cases | Person-years | Model1  HR (95% CI) | Model1 *P* | Model2  HR (95% CI) | Model2 *P* | Median | Cases | Person-years | Model1 HR (95% CI) | Model1 *P* | Model2  HR (95% CI) | Model2 *P* |  |
|  | **aMED** |  |  |  |  |  |  | **aMED** |  |  |  |  |  |  |  |
| Q1 | 2 | 16 | 170886 | Ref. |  | Ref. |  | 2 | 16 | 242695 | Ref. |  | Ref. |  |  |
| Q2 | 3 | 17 | 187294 | 0.91 [0.46, 1.80] | 0.783 | 0.91 [0.46, 1.81] | 0.794 | 3 | 16 | 325936 | 0.69 [0.34, 1.37] | 0.286 | 0.68 [0.34, 1.37] | 0.282 | 0.559 |
| Q3 | 4 | 17 | 175070 | 0.94 [0.47, 1.86] | 0.859 | 0.96 [0.48, 1.91] | 0.902 | 4 | 18 | 364086 | 0.66 [0.34, 1.30] | 0.230 | 0.67 [0.34, 1.31] | 0.239 | 0.465 |
| Q4 | 5 | 10 | 103833 | 0.91 [0.41, 2.00] | 0.808 | 0.93 [0.42, 2.07] | 0.863 | 5 | 11 | 257123 | 0.54 [0.25, 1.18] | 0.121 | 0.55 [0.25, 1.19] | 0.128 | 0.356 |
| Q5 | 6 | 2 | 52936 | 0.34 [0.08, 1.49] | 0.154 | 0.35 [0.08, 1.56] | 0.170 | 6 | 6 | 167905 | 0.42 [0.17, 1.09] | 0.074 | 0.43 [0.17, 1.12] | 0.084 | 0.819 |
| P for trend |  |  |  | 0.90 [0.73, 1.10] | 0.302 | 0.90 [0.73, 1.12] | 0.355 |  |  |  | 0.83 [0.68, 1.00] | 0.052 | 0.83 [0.68, 1.01] | 0.062 | 0.586 |
| per 1 SD |  |  |  | 0.85 [0.65, 1.10] | 0.213 | 0.86 [0.66, 1.12] | 0.255 |  |  |  | 0.78 [0.61, 1.00] | 0.046 | 0.78 [0.61, 1.00] | 0.054 | 0.597 |
|  | **PHD** |  |  |  |  |  |  | **PHD** |  |  |  |  |  |  |  |
| Q1 | 2 | 19 | 196567 | Ref. |  | Ref. |  | 2 | 13 | 292076 | Ref. |  | Ref. |  |  |
| Q2 | 3 | 12 | 173380 | 0.69 [0.33, 1.41] | 0.306 | 0.70 [0.34, 1.44] | 0.328 | 3 | 17 | 309689 | 1.17 [0.57, 2.40] | 0.677 | 1.17 [0.57, 2.41] | 0.674 | 0.324 |
| Q3 | 3.5 | 10 | 92648 | 1.03 [0.48, 2.22] | 0.940 | 1.06 [0.49, 2.30] | 0.876 | 3.5 | 8 | 204674 | 0.84 [0.35, 2.02] | 0.693 | 0.84 [0.35, 2.04] | 0.705 | 0.697 |
| Q4 | 4 | 15 | 119866 | 1.22 [0.62, 2.40] | 0.569 | 1.26 [0.64, 2.49] | 0.507 | 4 | 15 | 264581 | 1.19 [0.57, 2.51] | 0.639 | 1.18 [0.56, 2.49] | 0.666 | 0.899 |
| Q5 | 5 | 6 | 107557 | 0.53 [0.21, 1.34] | 0.182 | 0.56 [0.22, 1.41] | 0.219 | 5 | 14 | 286726 | 1.01 [0.48, 2.16] | 0.971 | 0.98 [0.46, 2.10] | 0.956 | 0.361 |
| P for trend |  |  |  | 0.92 [0.72, 1.18] | 0.508 | 0.93 [0.73, 1.20] | 0.594 |  |  |  | 1.00 [0.79, 1.27] | 0.973 | 0.99 [0.78, 1.25] | 0.946 | 0.721 |
| per 1 SD |  |  |  | 0.91 [0.70, 1.17] | 0.449 | 0.92 [0.71, 1.19] | 0.538 |  |  |  | 0.97 [0.76, 1.23] | 0.788 | 0.95 [0.75, 1.22] | 0.695 | 0.859 |
|  | **SMD** |  |  |  |  |  |  | **SMD** |  |  |  |  |  |  |  |
| Q1 | -2.6 | 11 | 136387 | Ref. |  | Ref. |  | -2.54 | 15 | 273889 | Ref. |  | Ref. |  |  |
| Q2 | -1.7 | 9 | 123299 | 0.90 [0.37, 2.17] | 0.814 | 0.94 [0.39, 2.29] | 0.894 | -1.69 | 22 | 286298 | 1.40 [0.73, 2.70] | 0.317 | 1.37 [0.71, 2.66] | 0.349 | 0.504 |
| Q3 | -1.2 | 13 | 124566 | 1.30 [0.58, 2.90] | 0.522 | 1.38 [0.61, 3.12] | 0.441 | -1.2 | 12 | 285511 | 0.79 [0.37, 1.69] | 0.546 | 0.75 [0.35, 1.63] | 0.474 | 0.287 |
| Q4 | -0.72 | 14 | 134971 | 1.33 [0.60, 2.92] | 0.483 | 1.41 [0.63, 3.18] | 0.403 | -0.74 | 12 | 274080 | 0.86 [0.40, 1.84] | 0.703 | 0.78 [0.36, 1.70] | 0.534 | 0.301 |
| Q5 | 0.03 | 15 | 170796 | 1.19 [0.55, 2.59] | 0.662 | 1.23 [0.55, 2.75] | 0.616 | -0.03 | 6 | 237967 | 0.54 [0.21, 1.39] | 0.200 | 0.46 [0.18, 1.23] | 0.121 | 0.124 |
| P for trend |  |  |  | 1.11 [0.83, 1.47] | 0.481 | 1.12 [0.84, 1.50] | 0.452 |  |  |  | 0.80 [0.60, 1.06] | 0.122 | 0.75 [0.56, 1.01] | 0.062 | 0.057 |
| per 1 SD |  |  |  | 0.99 [0.79, 1.25] | 0.953 | 0.99 [0.78, 1.26] | 0.953 |  |  |  | 0.89 [0.69,1.14] | 0.354 | 0.85 [0.66, 1.10] | 0.226 | 0.394 |
|  | **LCD** |  |  |  |  |  |  | **LCD** |  |  |  |  |  |  |  |
| Q1 | 2.33 | 8 | 142025 | Ref. |  | Ref. |  | 2.33 | 14 | 298390 | Ref. |  | Ref. |  |  |
| Q2 | 6 | 15 | 138744 | 1.87 [0.79, 4.40] | 0.154 | 1.84 [0.78, 4.35] | 0.163 | 6 | 15 | 292964 | 1.07 [0.52, 2.22] | 0.854 | 1.09 [0.52, 2.26] | 0.822 | 0.364 |
| Q3 | 9 | 12 | 146702 | 1.43 [0.59, 3.50] | 0.432 | 1.42 [0.58, 3.48] | 0.444 | 9 | 14 | 288915 | 1.03 [0.49, 2.16] | 0.936 | 1.02 [0.49, 2.15] | 0.955 | 0.577 |
| Q4 | 12 | 16 | 118281 | 2.38 [1.02, 5.56] | 0.046 | 2.34 [1.00, 5.49] | 0.050 | 12 | 16 | 225837 | 1.50 [0.73, 3.09] | 0.265 | 1.45 [0.70, 2.98] | 0.314 | 0.401 |
| Q5 | 17 | 11 | 144267 | 1.37 [0.55, 3.40] | 0.501 | 1.34 [0.54, 3.34] | 0.534 | 16.67 | 8 | 251638 | 0.70 [0.29, 1.67] | 0.422 | 0.65 [0.27, 1.57] | 0.342 | 0.263 |
| P for trend |  |  |  | 1.02 [0.97, 1.07] | 0.508 | 1.02 [0.97, 1.07] | 0.544 |  |  |  | 0.99 [0.94, 1.04] | 0.739 | 0.99 [0.94, 1.04] | 0.588 | 0.406 |
| per 1 SD |  |  |  | 1.06 [0.83, 1.35] | 0.644 | 1.05 [0.82, 1.34] | 0.690 |  |  |  | 0.92 [0.71, 1.19] | 0.534 | 0.90 [0.69, 1.16] | 0.409 | 0.398 |
|  | **aLCD** |  |  |  |  |  |  | **aLCD** |  |  |  |  |  |  |  |
| Q1 | 1 | 9 | 130105 | Ref. |  | Ref. |  | 1 | 12 | 295209 | Ref. |  | Ref. |  |  |
| Q2 | 4 | 11 | 126768 | 1.21 [0.50, 2.92] | 0.673 | 1.19 [0.49, 2.88] | 0.696 | 4 | 17 | 272952 | 1.48 [0.71, 3.11] | 0.295 | 1.50 [0.71, 3.15] | 0.287 | 0.695 |
| Q3 | 7 | 10 | 135915 | 1.02 [0.41, 2.51] | 0.963 | 1.01 [0.41, 2.50] | 0.980 | 7 | 13 | 273880 | 1.13 [0.52, 2.48] | 0.757 | 1.11 [0.50, 2.44] | 0.801 | 0.878 |
| Q4 | 10 | 18 | 145128 | 1.75 [0.78, 3.89] | 0.172 | 1.73 [0.77, 3.89] | 0.184 | 10 | 14 | 268400 | 1.26 [0.58, 2.73] | 0.557 | 1.19 [0.54, 2.60] | 0.666 | 0.516 |
| Q5 | 16 | 14 | 152105 | 1.32 [0.57, 3.04] | 0.521 | 1.30 [0.56, 3.04] | 0.539 | 15.67 | 11 | 247304 | 1.09 [0.48, 2.47] | 0.837 | 0.97 [0.42, 2.24] | 0.952 | 0.630 |
| P for trend |  |  |  | 1.02 [0.97, 1.07] | 0.388 | 1.02 [0.97, 1.07] | 0.405 |  |  |  | 1.00 [0.95, 1.05] | 0.937 | 0.99 [0.94, 1.04] | 0.679 | 0.406 |
| per 1 SD |  |  |  | 1.06 [0.84, 1.34] | 0.629 | 1.06 [0.83, 1.34] | 0.655 |  |  |  | 1.00 [0.78, 1.28] | 0.975 | 0.95 [0.74, 1.23] | 0.712 | 0.539 |
|  | **pLCD** |  |  |  |  |  |  | **pLCD** |  |  |  |  |  |  |  |
| Q1 | 11 | 9 | 130105 | Ref. |  | Ref. |  | 11 | 12 | 295209 | Ref. |  | Ref. |  |  |
| Q2 | 14 | 11 | 126768 | 1.04 [0.50, 2.18] | 0.915 | 1.03 [0.49, 2.16] | 0.941 | 14 | 17 | 272952 | 0.76 [0.39, 1.50] | 0.437 | 0.79 [0.40, 1.55] | 0.486 | 0.605 |
| Q3 | 16.5 | 10 | 135915 | 0.91 [0.41, 2.01] | 0.819 | 0.90 [0.41, 1.98] | 0.790 | 16.5 | 13 | 273880 | 0.54 [0.25, 1.15] | 0.110 | 0.55 [0.26, 1.19] | 0.132 | 0.378 |
| Q4 | 19 | 18 | 145128 | 0.94 [0.44, 2.04] | 0.880 | 0.93 [0.43, 2.01] | 0.848 | 19 | 14 | 268400 | 0.53 [0.25, 1.12] | 0.098 | 0.55 [0.26, 1.16] | 0.117 | 0.338 |
| Q5 | 22 | 14 | 152105 | 1.08 [0.49, 2.38] | 0.847 | 1.05 [0.47, 2.33] | 0.902 | 22 | 11 | 247304 | 0.70 [0.34, 1.43] | 0.325 | 0.70 [0.34, 1.45] | 0.337 | 0.462 |
| P for trend |  |  |  | 1.00 [0.94, 1.07] | 0.969 | 1.00 [0.93, 1.07] | 0.978 |  |  |  | 0.95 [0.90, 1.02] | 0.160 | 0.96 [0.90, 1.02] | 0.173 | 0.395 |
| p per 1 SD |  |  |  | 1.03 [0.80, 1.32] | 0.839 | 1.02 [0.79, 1.31] | 0.889 |  |  |  | 0.80 [0.63, 1.02] | 0.072 | 0.80 [0.63, 1.03] | 0.080 | 0.177 |
|  | **PDI** |  |  |  |  |  |  | **PDI** |  |  |  |  |  |  |  |
| Q1 | 44 | 20 | 180147 | Ref. |  | Ref. |  | 44 | 9 | 258938 | Ref. |  | Ref. |  |  |
| Q2 | 48 | 13 | 150151 | 0.76 [0.38, 1.53] | 0.439 | 0.76 [0.38, 1.53] | 0.445 | 48 | 13 | 272783 | 1.32 [0.57, 3.10] | 0.517 | 1.39 [0.59, 3.26] | 0.450 | 0.283 |
| Q3 | 50.5 | 6 | 118048 | 0.44 [0.18, 1.10] | 0.079 | 0.44 [0.18, 1.11] | 0.083 | 50.5 | 18 | 250680 | 1.97 [0.89, 4.40] | 0.096 | 2.16 [0.96, 4.84] | 0.061 | 0.010 |
| Q4 | 53 | 16 | 126217 | 1.10 [0.57, 2.13] | 0.778 | 1.09 [0.56, 2.14] | 0.794 | 53 | 13 | 285518 | 1.26 [0.54, 2.96] | 0.590 | 1.39 [0.59, 3.30] | 0.451 | 0.662 |
| Q5 | 57 | 7 | 115456 | 0.53 [0.22, 1.25] | 0.145 | 0.52 [0.22, 1.27] | 0.151 | 57 | 14 | 289826 | 1.33 [0.58, 3.08] | 0.502 | 1.51 [0.64, 3.59] | 0.349 | 0.089 |
| P for trend |  |  |  | 0.97 [0.92, 1.03] | 0.290 | 0.97 [0.91, 1.03] | 0.299 |  |  |  | 1.01 [0.96, 1.07] | 0.631 | 1.02 [0.97, 1.08] | 0.426 | 0.230 |
| per 1 SD |  |  |  | 0.89 [0.70, 1.14] | 0.363 | 0.89 [0.69, 1.15] | 0.377 |  |  |  | 1.05 [0.82, 1.34] | 0.720 | 1.09 [0.84, 1.42] | 0.497 | 0.278 |
|  | **hPDI** |  |  |  |  |  |  | **hPDI** |  |  |  |  |  |  |  |
| Q1 | 49.5 | 18 | 194561 | Ref. |  | Ref. |  | 50 | 12 | 241168 | Ref. |  | Ref. |  |  |
| Q2 | 54 | 13 | 143524 | 0.91 [0.44, 1.86] | 0.794 | 0.90 [0.44, 1.86] | 0.782 | 54 | 10 | 245259 | 0.74 [0.32, 1.71] | 0.477 | 0.72 [0.31, 1.67] | 0.443 | 0.693 |
| Q3 | 57 | 12 | 138748 | 0.85 [0.41, 1.76] | 0.657 | 0.84 [0.40, 1.78] | 0.653 | 57 | 18 | 280832 | 1.10 [0.53, 2.30] | 0.792 | 1.07 [0.51, 2.26] | 0.852 | 0.653 |
| Q4 | 60 | 14 | 114146 | 1.18 [0.58, 2.38] | 0.650 | 1.18 [0.57, 2.44] | 0.652 | 60 | 11 | 281844 | 0.67 [0.29, 1.53] | 0.340 | 0.65 [0.28, 1.51] | 0.316 | 0.294 |
| Q5 | 64 | 5 | 99040 | 0.49 [0.18, 1.32] | 0.156 | 0.49 [0.18, 1.35] | 0.169 | 64 | 16 | 308641 | 0.89 [0.42, 1.90] | 0.760 | 0.87 [0.40, 1.89] | 0.717 | 0.376 |
| P for trend |  |  |  | 0.98 [0.93, 1.03] | 0.389 | 0.98 [0.92, 1.03] | 0.412 |  |  |  | 0.99 [0.94, 1.04] | 0.733 | 0.99 [0.94, 1.04] | 0.702 | 0.793 |
| per 1 SD |  |  |  | 0.87 [0.67, 1.13] | 0.306 | 0.87 [0.66, 1.15] | 0.322 |  |  |  | 0.98 [0.76, 1.26] | 0.853 | 0.97 [0.74, 1.26] | 0.817 | 0.579 |
|  | **uPDI** |  |  |  |  |  |  | **uPDI** |  |  |  |  |  |  |  |
| Q1 | 50 | 10 | 129967 | Ref. |  | Ref. |  | 50 | 10 | 303459 | Ref. |  | Ref. |  |  |
| Q2 | 54 | 10 | 125752 | 1.06 [0.44, 2.54] | 0.901 | 1.07 [0.45, 2.59] | 0.872 | 54 | 14 | 277969 | 1.62 [0.72, 3.65] | 0.244 | 1.60 [0.71, 3.61] | 0.257 | 0.509 |
| Q3 | 57 | 14 | 131620 | 1.47 [0.65, 3.32] | 0.350 | 1.51 [0.67, 3.42] | 0.323 | 57 | 15 | 266400 | 1.91 [0.86, 4.24] | 0.115 | 1.86 [0.83, 4.16] | 0.132 | 0.722 |
| Q4 | 60 | 14 | 143234 | 1.39 [0.62, 3.13] | 0.427 | 1.43 [0.63, 3.25] | 0.391 | 60 | 14 | 264802 | 1.92 [0.85, 4.32] | 0.117 | 1.85 [0.82, 4.20] | 0.141 | 0.663 |
| Q5 | 64 | 14 | 159446 | 1.37 [0.61, 3.10] | 0.449 | 1.43 [0.62, 3.27] | 0.397 | 64 | 14 | 245116 | 2.41 [1.07, 5.45] | 0.035 | 2.31 [1.01, 5.30] | 0.047 | 0.423 |
| P for trend |  |  |  | 1.02 [0.97, 1.08] | 0.354 | 1.03 [0.98, 1.08] | 0.309 |  |  |  | 1.06 [1.00, 1.11] | 0.032 | 1.05 [1.00, 1.11] | 0.047 | 0.597 |
| per 1 SD |  |  |  | 1.13 [0.88, 1.45] | 0.352 | 1.14 [0.89, 1.48] | 0.304 |  |  |  | 1.29 [1.00, 1.66] | 0.047 | 1.27 [0.98, 1.64] | 0.07 | 0.558 |

The nine dietary pattern scores were divided into quintiles and treated as categorical variables. Model 1 was adjusted for age at first completion of the Oxford WebQ and sex, and Model 2 was further adjusted for race, educational attainment, TDI, family history of cancer, smoking status, average alcohol intake (except aMED & SMD), MET, average energy intake, BMI, WHR and T2DM. The *p*-heterogeneity was obtained using the contrast method based on a fully unconstrained approach.

MAFLD, metabolic dysfunction-associated fatty liver disease; HR, hazard ratio; CI, confidence interval; aMED, alternate Mediterranean diet; PHD, Planetary Health Diet; SMD, sulfur microbial diet; LCD, Low-carbohydrate Diet; aLCD, animal based Low-carbohydrate Diet; pLCD, plant based Low-carbohydrate Diet; PDI, Plant-based Diet Index; hPDI, healthful Plant-based Diet Index; uPDI, unhealthful Plant-based Diet Index.

**Table S15. The associations of the scores of dietary patterns with the mortality of liver disease according to MAFLD status.**

|  | **MAFLD (59,** **473)** | | | | | | | **Non-MAFLD (117,211)** | | | | | | | ***p*-heterogeneity** |
| --- | --- | --- | --- | --- | --- | --- | --- | --- | --- | --- | --- | --- | --- | --- | --- |
|  | Median | Cases | Person-years | Model1  HR (95% CI) | Model1 *P* | Model2  HR (95% CI) | Model2 *P* | Median | Cases | Person-years | Model1 HR (95% CI) | Model1 *P* | Model2  HR (95% CI) | Model2 *P* |  |
|  | **aMED** |  |  |  |  |  |  | **aMED** |  |  |  |  |  |  |  |
| Q1 | 2 | 43 | 165613 | Ref. |  | Ref. |  | 2 | 22 | 237871 | Ref. |  | Ref. |  |  |
| Q2 | 3 | 41 | 181550 | 0.81 [0.53, 1.24] | 0.332 | 0.85 [0.55, 1.30] | 0.443 | 3 | 21 | 320187 | 0.66 [0.36, 1.21] | 0.178 | 0.69 [0.38, 1.26] | 0.231 | 0.580 |
| Q3 | 4 | 29 | 170420 | 0.59 [0.37, 0.94] | 0.027 | 0.63 [0.39, 1.02] | 0.058 | 4 | 19 | 358142 | 0.52 [0.28, 0.96] | 0.036 | 0.55 [0.30, 1.03] | 0.061 | 0.734 |
| Q4 | 5 | 26 | 101163 | 0.86 [0.53, 1.41] | 0.560 | 0.96 [0.59, 1.58] | 0.876 | 5 | 18 | 253363 | 0.67 [0.36, 1.25] | 0.204 | 0.73 [0.39, 1.37] | 0.330 | 0.501 |
| Q5 | 6 | 8 | 51324 | 0.50 [0.24, 1.07] | 0.073 | 0.58 [0.27, 1.24] | 0.157 | 6 | 10 | 165267 | 0.54 [0.26, 1.14] | 0.108 | 0.62 [0.29, 1.33] | 0.218 | 0.903 |
| P for trend |  |  |  | 0.88 [0.77, 1.01] | 0.073 | 0.91 [0.80, 1.05] | 0.205 |  |  |  | 0.87 [0.74, 1.03] | 0.113 | 0.90 [0.76, 1.07] | 0.233 | 0.921 |
| per 1 SD |  |  |  | 0.85 [0.72, 1.01] | 0.061 | 0.89 [0.75, 1.06] | 0.181 |  |  |  | 0.84 [0.68, 1.04] | 0.108 | 0.88 [0.71, 1.09] | 0.226 | 0.936 |
|  | **PHD** |  |  |  |  |  |  | **PHD** |  |  |  |  |  |  |  |
| Q1 | 2 | 55 | 190463 | Ref. |  | Ref. |  | 2 | 21 | 286224 | Ref. |  | Ref. |  |  |
| Q2 | 3 | 22 | 167978 | 0.44 [0.27, 0.72] | 0.001 | 0.45 [0.28, 0.74] | 0.002 | 3 | 22 | 304042 | 0.95 [0.52, 1.72] | 0.856 | 0.97 [0.53, 1.77] | 0.923 | 0.052 |
| Q3 | 3.5 | 20 | 90395 | 0.71 [0.43, 1.19] | 0.192 | 0.76 [0.46, 1.28] | 0.303 | 3.5 | 9 | 201642 | 0.59 [0.27, 1.28] | 0.181 | 0.61 [0.28, 1.34] | 0.223 | 0.645 |
| Q4 | 4 | 29 | 116743 | 0.82 [0.52, 1.29] | 0.386 | 0.87 [0.55, 1.36] | 0.538 | 4 | 16 | 260516 | 0.80 [0.42, 1.53] | 0.500 | 0.83 [0.43, 1.59] | 0.567 | 0.907 |
| Q5 | 5 | 21 | 104491 | 0.66 [0.40, 1.09] | 0.105 | 0.71 [0.43, 1.18] | 0.188 | 5 | 22 | 282406 | 1.01 [0.55, 1.84] | 0.975 | 1.05 [0.57, 1.91] | 0.886 | 0.330 |
| P for trend |  |  |  | 0.90 [0.76, 1.05] | 0.181 | 0.92 [0.78, 1.08] | 0.318 |  |  |  | 0.99 [0.81, 1.21] | 0.909 | 1.00 [0.82, 1.22] | 0.998 | 0.525 |
| per 1 SD |  |  |  | 0.89 [0.75, 1.05] | 0.162 | 0.92 [0.77, 1.08] | 0.302 |  |  |  | 0.94 [0.76, 1.16] | 0.543 | 0.95 [0.77, 1.17] | 0.617 | 0.815 |
|  | **SMD** |  |  |  |  |  |  | **SMD** |  |  |  |  |  |  |  |
| Q1 | -2.6 | 30 | 132217 | Ref. |  | Ref. |  | -2.54 | 21 | 268820 | Ref. |  | Ref. |  |  |
| Q2 | -1.7 | 21 | 119780 | 0.78 [0.44, 1.36] | 0.373 | 0.77 [0.44, 1.36] | 0.368 | -1.69 | 27 | 281676 | 1.23 [0.69, 2.17] | 0.479 | 1.21 [0.68, 2.15] | 0.522 | 0.272 |
| Q3 | -1.2 | 26 | 121163 | 0.97 [0.57, 1.63] | 0.900 | 0.94 [0.55, 1.61] | 0.831 | -1.2 | 14 | 281032 | 0.66 [0.33, 1.29] | 0.221 | 0.62 [0.31, 1.24] | 0.178 | 0.352 |
| Q4 | -0.72 | 25 | 131255 | 0.88 [0.52, 1.50] | 0.645 | 0.83 [0.48, 1.43] | 0.498 | -0.74 | 13 | 269607 | 0.66 [0.33, 1.31] | 0.232 | 0.59 [0.29, 1.20] | 0.144 | 0.455 |
| Q5 | 0.03 | 45 | 165654 | 1.33 [0.84, 2.11] | 0.228 | 1.12 [0.69, 1.81] | 0.644 | -0.03 | 15 | 233694 | 0.93 [0.48, 1.80] | 0.821 | 0.79 [0.39, 1.56] | 0.490 | 0.417 |
| P for trend |  |  |  | 1.14 [0.95, 1.36] | 0.173 | 1.06 [0.88, 1.28] | 0.538 |  |  |  | 0.88 [0.69, 1.12] | 0.302 | 0.83 [0.64, 1.07] | 0.142 | 0.132 |
| per 1 SD |  |  |  | 1.08 [0.93, 1.25] | 0.341 | 1.01 [0.87, 1.18] | 0.874 |  |  |  | 0.94 [0.75, 1.17] | 0.565 | 0.89 [0.71, 1.12] | 0.320 | 0.366 |
|  | **LCD** |  |  |  |  |  |  | **LCD** |  |  |  |  |  |  |  |
| Q1 | 2.33 | 28 | 137417 | Ref. |  | Ref. |  | 2.33 | 21 | 292900 | Ref. |  | Ref. |  |  |
| Q2 | 6 | 35 | 134738 | 1.24 [0.75, 2.04] | 0.395 | 1.17 [0.71, 1.92] | 0.544 | 6 | 23 | 287835 | 1.10 [0.61, 1.98] | 0.758 | 1.10 [0.61, 1.99] | 0.751 | 0.876 |
| Q3 | 9 | 31 | 142498 | 1.07 [0.64, 1.78] | 0.800 | 0.94 [0.56, 1.57] | 0.812 | 9 | 17 | 284387 | 0.83 [0.44, 1.58] | 0.575 | 0.81 [0.42, 1.53] | 0.512 | 0.724 |
| Q4 | 12 | 26 | 115024 | 1.12 [0.66, 1.92] | 0.671 | 0.98 [0.57, 1.68] | 0.949 | 12 | 15 | 222241 | 0.94 [0.49, 1.83] | 0.864 | 0.89 [0.46, 1.73] | 0.725 | 0.825 |
| Q5 | 17 | 27 | 140392 | 0.99 [0.58, 1.68] | 0.973 | 0.84 [0.49, 1.42] | 0.508 | 16.67 | 14 | 247466 | 0.82 [0.42, 1.62] | 0.570 | 0.75 [0.38, 1.49] | 0.417 | 0.798 |
| P for trend |  |  |  | 1.00 [0.96, 1.03] | 0.805 | 0.98 [0.95, 1.02] | 0.350 |  |  |  | 0.98 [0.94, 1.03] | 0.470 | 0.98 [0.94, 1.02] | 0.309 | 1.000 |
| per 1 SD |  |  |  | 0.97 [0.83, 1.14] | 0.724 | 0.92 [0.78, 1.08] | 0.306 |  |  |  | 0.87 [0.70, 1.09] | 0.234 | 0.84 [0.67, 1.06] | 0.140 | 0.526 |
|  | **aLCD** |  |  |  |  |  |  | **aLCD** |  |  |  |  |  |  |  |
| Q1 | 1 | 26 | 126070 | Ref. |  | Ref. |  | 1 | 24 | 290001 | Ref. |  | Ref. |  |  |
| Q2 | 4 | 27 | 123245 | 1.02 [0.59, 1.74] | 0.954 | 0.95 [0.55, 1.63] | 0.851 | 4 | 19 | 268527 | 0.83 [0.46, 1.52] | 0.555 | 0.80 [0.44, 1.46] | 0.467 | 0.677 |
| Q3 | 7 | 27 | 131939 | 0.95 [0.55, 1.62] | 0.843 | 0.82 [0.48, 1.42] | 0.481 | 7 | 12 | 269224 | 0.53 [0.26, 1.05] | 0.070 | 0.49 [0.24, 0.98] | 0.045 | 0.256 |
| Q4 | 10 | 33 | 140997 | 1.11 [0.66, 1.85] | 0.697 | 0.89 [0.53, 1.50] | 0.664 | 10 | 19 | 264119 | 0.86 [0.47, 1.58] | 0.631 | 0.76 [0.41, 1.40] | 0.382 | 0.701 |
| Q5 | 16 | 34 | 147818 | 1.12 [0.67, 1.87] | 0.658 | 0.87 [0.52, 1.47] | 0.613 | 15.67 | 16 | 242960 | 0.81 [0.43, 1.52] | 0.503 | 0.68 [0.35, 1.29] | 0.234 | 0.563 |
| P for trend |  |  |  | 1.01 [0.98, 1.04] | 0.562 | 0.99 [0.96, 1.02] | 0.644 |  |  |  | 0.99 [0.95, 1.03] | 0.586 | 0.98 [0.94, 1.02] | 0.282 | 0.696 |
| per 1 SD |  |  |  | 1.01 [0.87, 1.19] | 0.856 | 0.93 [0.79, 1.10] | 0.395 |  |  |  | 0.92 [0.74, 1.15] | 0.476 | 0.86 [0.69, 1.09] | 0.213 | 0.587 |
|  | **pLCD** |  |  |  |  |  |  | **pLCD** |  |  |  |  |  |  |  |
| Q1 | 11 | 26 | 126070 | Ref. |  | Ref. |  | 11 | 24 | 290001 | Ref. |  | Ref. |  |  |
| Q2 | 14 | 27 | 123245 | 0.62 [0.38, 1.01] | 0.056 | 0.62 [0.38, 1.01] | 0.053 | 14 | 19 | 268527 | 1.11 [0.60, 2.06] | 0.729 | 1.15 [0.62, 2.14] | 0.648 | 0.125 |
| Q3 | 16.5 | 27 | 131939 | 0.66 [0.41, 1.09] | 0.104 | 0.68 [0.41, 1.12] | 0.128 | 16.5 | 12 | 269224 | 1.06 [0.56, 1.98] | 0.864 | 1.11 [0.59, 2.09] | 0.741 | 0.234 |
| Q4 | 19 | 33 | 140997 | 0.71 [0.44, 1.15] | 0.161 | 0.73 [0.45, 1.18] | 0.197 | 19 | 19 | 264119 | 0.90 [0.48, 1.71] | 0.753 | 0.95 [0.50, 1.81] | 0.885 | 0.521 |
| Q5 | 22 | 34 | 147818 | 0.79 [0.48, 1.29] | 0.346 | 0.77 [0.47, 1.26] | 0.292 | 22 | 16 | 242960 | 0.57 [0.26, 1.22] | 0.146 | 0.58 [0.27, 1.25] | 0.166 | 0.542 |
| P for trend |  |  |  | 0.98 [0.94, 1.02] | 0.357 | 0.98 [0.94, 1.02] | 0.349 |  |  |  | 0.96 [0.91, 1.01] | 0.137 | 0.96 [0.91, 1.02] | 0.171 | 0.565 |
| per 1 SD |  |  |  | 0.93 [0.79, 1.09] | 0.371 | 0.92 [0.78, 1.09] | 0.330 |  |  |  | 0.85 [0.69, 1.05] | 0.130 | 0.86 [0.70, 1.06] | 0.161 | 0.620 |
|  | **PDI** |  |  |  |  |  |  | **PDI** |  |  |  |  |  |  |  |
| Q1 | 44 | 43 | 174573 | Ref. |  | Ref. |  | 44 | 16 | 253847 | Ref. |  | Ref. |  |  |
| Q2 | 48 | 39 | 145917 | 1.07 [0.70, 1.65] | 0.752 | 1.19 [0.77, 1.83] | 0.443 | 48 | 17 | 268046 | 0.98 [0.50, 1.95] | 0.962 | 1.06 [0.54, 2.11] | 0.860 | 0.779 |
| Q3 | 50.5 | 22 | 114476 | 0.77 [0.46, 1.29] | 0.326 | 0.92 [0.55, 1.55] | 0.754 | 50.5 | 21 | 246705 | 1.31 [0.68, 2.51] | 0.415 | 1.50 [0.78, 2.90] | 0.226 | 0.252 |
| Q4 | 53 | 25 | 122773 | 0.82 [0.50, 1.35] | 0.445 | 1.03 [0.62, 1.70] | 0.911 | 53 | 16 | 281093 | 0.88 [0.44, 1.77] | 0.728 | 1.05 [0.52, 2.13] | 0.884 | 0.965 |
| Q5 | 57 | 18 | 112329 | 0.66 [0.38, 1.14] | 0.135 | 0.86 [0.49, 1.53] | 0.615 | 57 | 20 | 285139 | 1.09 [0.57, 2.11] | 0.794 | 1.38 [0.70, 2.74] | 0.352 | 0.297 |
| P for trend |  |  |  | 0.97 [0.93, 1.00] | 0.084 | 0.99 [0.95, 1.03] | 0.601 |  |  |  | 1.00 [0.96, 1.05] | 0.895 | 1.02 [0.97, 1.07] | 0.391 | 0.357 |
| per 1 SD |  |  |  | 0.87 [0.75, 1.03] | 0.101 | 0.97 [0.82, 1.15] | 0.719 |  |  |  | 1.03 [0.83, 1.28] | 0.766 | 1.13 [0.90, 1.41] | 0.285 | 0.287 |
|  | **hPDI** |  |  |  |  |  |  | **hPDI** |  |  |  |  |  |  |  |
| Q1 | 49.5 | 50 | 188822 | Ref. |  | Ref. |  | 50 | 16 | 236295 | Ref. |  | Ref. |  |  |
| Q2 | 54 | 23 | 139013 | 0.59 [0.36, 0.96] | 0.034 | 0.56 [0.34, 0.92] | 0.023 | 54 | 18 | 241006 | 1.02 [0.52, 2.00] | 0.956 | 0.99 [0.50, 1.94] | 0.966 | 0.184 |
| Q3 | 57 | 34 | 134774 | 0.88 [0.57, 1.37] | 0.577 | 0.85 [0.54, 1.33] | 0.476 | 57 | 21 | 276053 | 1.01 [0.52, 1.94] | 0.984 | 0.96 [0.49, 1.86] | 0.903 | 0.767 |
| Q4 | 60 | 23 | 111094 | 0.72 [0.44, 1.18] | 0.197 | 0.68 [0.41, 1.14] | 0.141 | 60 | 13 | 277440 | 0.62 [0.30, 1.30] | 0.203 | 0.59 [0.28, 1.24] | 0.163 | 0.758 |
| Q5 | 64 | 17 | 96367 | 0.63 [0.36, 1.10] | 0.104 | 0.61 [0.34, 1.07] | 0.084 | 64 | 22 | 304036 | 0.96 [0.50, 1.85] | 0.901 | 0.91 [0.47, 1.79] | 0.794 | 0.373 |
| P for trend |  |  |  | 0.97 [0.94, 1.01] | 0.140 | 0.97 [0.94, 1.01] | 0.113 |  |  |  | 0.99 [0.94, 1.03] | 0.565 | 0.98 [0.94, 1.03] | 0.490 | 0.730 |
| per 1 SD |  |  |  | 0.87 [0.74, 1.03] | 0.113 | 0.85 [0.71, 1.02] | 0.080 |  |  |  | 0.92 [0.74, 1.15] | 0.475 | 0.91 [0.72, 1.14] | 0.394 | 0.648 |
|  | **uPDI** |  |  |  |  |  |  | **uPDI** |  |  |  |  |  |  |  |
| Q1 | 50 | 24 | 126261 | Ref. |  | Ref. |  | 50 | 19 | 298273 | Ref. |  | Ref. |  |  |
| Q2 | 54 | 22 | 121932 | 0.96 [0.54, 1.72] | 0.897 | 0.95 [0.53, 1.70] | 0.865 | 54 | 17 | 273379 | 1.02 [0.53, 1.95] | 0.963 | 0.99 [0.51, 1.90] | 0.972 | 0.927 |
| Q3 | 57 | 27 | 127745 | 1.18 [0.68, 2.04] | 0.559 | 1.13 [0.65, 1.97] | 0.656 | 57 | 16 | 262063 | 1.04 [0.53, 2.02] | 0.918 | 0.98 [0.50, 1.92] | 0.959 | 0.749 |
| Q4 | 60 | 27 | 139151 | 1.11 [0.64, 1.93] | 0.706 | 1.05 [0.60, 1.84] | 0.856 | 60 | 12 | 260361 | 0.82 [0.40, 1.70] | 0.603 | 0.78 [0.37, 1.61] | 0.499 | 0.529 |
| Q5 | 64 | 47 | 154980 | 1.93 [1.18, 3.17] | 0.009 | 1.83 [1.10, 3.02] | 0.019 | 64 | 26 | 240753 | 2.18 [1.20, 3.97] | 0.011 | 2.00 [1.09, 3.69] | 0.026 | 0.826 |
| P for trend |  |  |  | 1.05 [1.01, 1.09] | 0.005 | 1.05 [1.01, 1.08] | 0.011 |  |  |  | 1.05 [1.00, 1.10] | 0.031 | 1.04 [1.00, 1.09] | 0.064 | 0.731 |
| per 1 SD |  |  |  | 1.25 [1.06, 1.48] | 0.008 | 1.23 [1.04, 1.45] | 0.018 |  |  |  | 1.33 [1.07, 1.65] | 0.011 | 1.29 [1.03, 1.62] | 0.025 | 0.740 |

The nine dietary pattern scores were divided into quintiles and treated as categorical variables. Model 1 was adjusted for age at first completion of the Oxford WebQ and sex, and Model 2 was further adjusted for race, educational attainment, TDI, family history of cancer, smoking status, average alcohol intake (except aMED & SMD), MET, average energy intake, BMI, WHR and T2DM. The *p*-heterogeneity was obtained using the contrast method based on a fully unconstrained approach.

MAFLD, metabolic dysfunction-associated fatty liver disease; HR, hazard ratio; CI, confidence interval; aMED, alternate Mediterranean diet; PHD, Planetary Health Diet; SMD, sulfur microbial diet; LCD, Low-carbohydrate Diet; aLCD, animal based Low-carbohydrate Diet; pLCD, plant based Low-carbohydrate Diet; PDI, Plant-based Diet Index; hPDI, healthful Plant-based Diet Index; uPDI, unhealthful Plant-based Diet Index.

**Table S16. Sensitivity analyses for associations of MAFLD with the incidence and mortality of liver diseases in the UK Biobank cohort.**

| **Outcome** | **Excluding new-onset individuals with MAFLD** | | **Excluding participants of others race** | | **Excluding outcome events occurred during the first 2-years follow up** | |
| --- | --- | --- | --- | --- | --- | --- |
|  | **HR (95% CI)** | ***P*** | **HR (95% CI)** | ***P*** | **HR (95% CI)** | ***P*** |
| CLD | 2.85  [2.57, 3.16] | <0.001 | 2.91  [2.61, 3.24] | <0.001 | 2.88  [2.58, 3.22] | <0.001 |
| SLD | 2.42  [2.21, 2.65] | <0.001 | 2.47  [2.25, 2.72] | <0.001 | 2.51  [2.28, 2.77] | <0.001 |
| LC | 1.93  [1.67, 2.23] | <0.001 | 1.79  [1.53, 2.09] | <0.001 | 1.81  [1.55, 2.13] | <0.001 |
| HCC | 3.01  [2.34, 3.88] | <0.001 | 2.70  [2.07, 3.53] | <0.001 | 2.78  [2.11, 3.66] | <0.001 |
| ICC | 1.42  [1.14, 1.76] | 0.002 | 1.36  [1.08, 1.71] | 0.009 | 1.37  [1.08, 1.73] | 0.010 |
| LRD | 2.25  [1.94, 2.61] | <0.001 | 2.28  [1.96, 2.64] | <0.001 | 2.30  [1.97, 2.68] | <0.001 |

The Cox regression model derived HRs, 95% CIs, and P-values using a fully adjusted model that accounted for age at recruitment, sex, TDI, smoking and drinking status, educational attainment, physical activity, and family history of cancer, and additionally adjusted for race in the analysis which excluded individuals with new-onset MAFLD.

MAFLD, metabolic dysfunction-associated fatty liver disease; HR, hazard ratio; CI, confidence interval; CLD, chronic liver disease; SLD, severe liver disease; LC, liver cancer; HCC, hepatocellular carcinoma; ICC, intrahepatic cholangiocarcinoma; LRD, liver-related death. CLD, chronic liver disease; SLD, severe liver disease; LC, liver cancer; HCC, hepatocellular carcinoma; ICC, intrahepatic cholangiocarcinoma; LRD, liver-related death.

**Table S17. Sensitivity analyses for associations of the scores of dietary patterns with the incidence and mortality of liver diseases in the UK Biobank cohort.**

|  | **Chronic liver disease (CLD)** | | | | | | **Severe liver disease (SLD)** | | | | | | **Liver-related death (LRD)** | | | | | |
| --- | --- | --- | --- | --- | --- | --- | --- | --- | --- | --- | --- | --- | --- | --- | --- | --- | --- | --- |
|  | Excluding new-onset individuals with MAFLD | | Excluding participants of others race | | Excluding outcome events occurred during the first 2-years follow up | | Excluding new-onset individuals with MAFLD | | Excluding participants of others race | | Excluding outcome events occurred during the first 2-years follow up | | Excluding new-onset individuals with MAFLD | | Excluding participants of others race | | Excluding outcome events occurred during the first 2-years follow up | |
|  | HR  (95% CI) | P | HR  (95% CI) | P | HR  (95% CI) | P | HR  (95% CI) | P | HR  (95% CI) | P | HR  (95% CI) | P | HR  (95% CI) | P | HR  (95% CI) | P | HR  (95% CI) | P |
| **aMED** |  |  |  |  |  |  |  |  |  |  |  |  |  |  |  |  |  |  |
| quintile 5 vs 1 | 0.53  [0.36, 0.74] | 0.001 | 0.55  [0.38, 0.81] | 0.002 | 0.59  [0.40, 0.88] | 0.009 | 0.51  [0.36, 0.72] | <0.001 | 0.52  [0.37, 0.74] | <0.001 | 0.55  [0.38, 0.79] | 0.001 | 0.56  [0.32, 0.99] | 0.046 | 0.54  [0.30, 0.96] | 0.036 | 0.54  [0.29, 0.98] | 0.042 |
| per 1 SD | 0.82  [0.75, 0.90] | <0.001 | 0.83  [0.76, 0.92] | <0.001 | 0.84  [0.76, 0.93] | 0.001 | 0.84  [0.77, 0.91] | <0.001 | 0.84  [0.77, 0.92] | <0.001 | 0.84  [0.77, 0.92] | <0.001 | 0.85  [0.74, 0.98] | 0.028 | 0.85  [0.73, 0.98] | 0.024 | 0.85  [0.73, 0.98] | 0.028 |
| **PHD** |  | | | | | | | | | | | | | | | | | |
| quintile 5 vs 1 | 0.63  [0.47, 0.85] | 0.002 | 0.68  [0.50, 0.91] | 0.009 | 0.75  [0.56, 1.02] | 0.067 | 0.70  [0.54, 0.90] | 0.005 | 0.73  [0.56, 0.94] | 0.015 | 0.77  [0.59, 1.01] | 0.063 | 0.82  [0.55, 1.23] | 0.340 | 0.84  [0.56, 1.26] | 0.395 | 0.80  [0.52, 1.22] | 0.299 |
| per 1 SD | 0.86  [0.79, 0.95] | 0.002 | 0.88  [0.80, 0.97] | 0.007 | 0.91  [0.83, 1.00] | 0.062 | 0.89  [0.81, 0.96] | 0.004 | 0.90  [0.82, 0.97] | 0.011 | 0.92  [0.84, 1.00] | 0.059 | 0.95  [0.82, 1.09] | 0.431 | 0.95  [0.82, 1.09] | 0.442 | 0.94  [0.81, 1.09] | 0.420 |
| **SMD** |  |  |  |  |  |  |  |  |  |  |  |  |  |  |  |  |  |  |
| quintile 5 vs 1 | 1.15  [0.87, 1.53] | 0.326 | 1.17  [0.88, 1.56] | 0.271 | 1.20  [0.89, 1.62] | 0.221 | 1.20  [0.94, 1.55] | 0.144 | 1.24  [0.96, 1.60] | 0.097 | 1.24  [0.95, 1.62] | 0.114 | 1.01  [0.66, 1.54] | 0.964 | 1.07  [0.70, 1.65] | 0.754 | 0.98  [0.63, 1.54] | 0.947 |
| per 1 SD | 1.09  [1.00, 1.19] | 0.061 | 1.10  [1.00, 1.20] | 0.043 | 1.11  [1.01, 1.21] | 0.033 | 1.08  [1.00, 1.17] | 0.049 | 1.09  [1.01, 1.18] | 0.029 | 1.10  [1.01, 1.20] | 0.027 | 0.97  [0.85, 1.11] | 0.673 | 0.98  [0.86, 1.13] | 0.801 | 0.96  [0.83, 1.11] | 0.580 |
| **LCD** |  |  |  |  |  |  |  |  |  |  |  |  |  |  |  |  |  |  |
| quintile 5 vs 1 | 1.13  [0.84, 1.52] | 0.437 | 1.11  [0.82, 1.50] | 0.515 | 1.10  [0.80, 1.50] | 0.557 | 1.01  [0.78, 1.32] | 0.921 | 1.02  [0.78, 1.34] | 0.866 | 1.04  [0.78, 1.38] | 0.793 | 0.81  [0.52, 1.26] | 0.348 | 0.79  [0.51, 1.23] | 0.296 | 0.84  [0.54, 1.32] | 0.459 |
| per 1 SD | 1.00  [0.92, 1.10] | 0.922 | 0.99  [0.91, 1.09] | 0.897 | 1.00  [0.90, 1.10] | 0.920 | 0.98  [0.90, 1.06] | 0.613 | 0.98  [0.90, 1.06] | 0.611 | 0.98  [0.89, 1.07] | 0.596 | 0.89  [0.77, 1.03] | 0.110 | 0.89  [0.77, 1.03] | 0.115 | 0.90  [0.78, 1.04] | 0.164 |
| **aLCD** |  |  |  |  |  |  |  |  |  |  |  |  |  |  |  |  |  |  |
| quintile 5 vs 1 | 1.24  [0.92, 1.67] | 0.166 | 1.18  [0.87, 1.60] | 0.284 | 1.20  [0.88, 1.66] | 0.253 | 1.11  [0.85, 1.43] | 0.441 | 1.10  [0.85, 1.44] | 0.466 | 1.12  [0.85, 1.49] | 0.414 | 0.76  [0.50, 1.16] | 0.209 | 0.76  [0.50, 1.16] | 0.208 | 0.80  [0.52, 1.24] | 0.315 |
| per 1 SD | 1.04  [0.95, 1.14] | 0.362 | 1.02  [0.94, 1.12] | 0.609 | 1.02  [0.93, 1.12] | 0.686 | 1.02  [0.94, 1.10] | 0.698 | 1.01  [0.93, 1.10] | 0.800 | 1.00  [0.92, 1.09] | 0.935 | 0.91  [0.79, 1.05] | 0.199 | 0.91  [0.79, 1.06] | 0.221 | 0.92  [0.79, 1.06] | 0.248 |
| **pLCD** |  |  |  |  |  |  |  |  |  |  |  |  |  |  |  |  |  |  |
| quintile 5 vs 1 | 0.62  [0.46, 0.85] | 0.003 | 0.65  [0.47, 0.89] | 0.006 | 0.69  [0.50, 0.95] | 0.022 | 0.62  [0.47, 0.82] | 0.001 | 0.64  [0.48, 0.85] | 0.002 | 0.68  [0.50, 0.91] | 0.010 | 0.68  [0.44, 1.06] | 0.089 | 0.66  [0.42, 1.04] | 0.075 | 0.70  [0.44, 1.11] | 0.131 |
| per 1 SD | 0.87  [0.80, 0.95] | 0.003 | 0.89  [0.81, 0.97] | 0.010 | 0.91  [0.82, 1.00] | 0.042 | 0.87  [0.80, 0.94] | 0.001 | 0.88  [0.81, 0.96] | 0.003 | 0.90  [0.82, 0.98] | 0.014 | 0.89  [0.78, 1.02] | 0.090 | 0.89  [0.77, 1.02] | 0.083 | 0.91  [0.79, 1.05] | 0.185 |
| **PDI** |  |  |  |  |  |  |  |  |  |  |  |  |  |  |  |  |  |  |
| quintile 5 vs 1 | 0.83  [0.61, 1.13] | 0.240 | 0.85  [0.62, 1.16] | 0.295 | 0.81  [0.58, 1.13] | 0.218 | 0.87  [0.67, 1.14] | 0.321 | 0.84  [0.64, 1.11] | 0.230 | 0.86  [0.64, 1.14] | 0.296 | 1.05  [0.67, 1.65] | 0.820 | 1.06  [0.67, 1.68 | 0.789 | 1.02  [0.63, 1.65] | 0.940 |
| per 1 SD | 0.89  [0.81, 0.98] | 0.017 | 0.90  [0.82, 0.99] | 0.034 | 0.90  [0.81, 0.99] | 0.029 | 0.92  [0.84, 0.99] | 0.038 | 0.91  [0.84, 0.99] | 0.035 | 0.91  [0.84, 1.00] | 0.050 | 1.01  [0.88, 1.16] | 0.896 | 1.01  [0.88, 1.17] | 0.890 | 1.00  [0.86, 1.16] | 0.983 |
| **hPDI** |  |  |  |  |  |  |  |  |  |  |  |  |  |  |  |  |  |  |
| quintile 5 vs 1 | 0.69  [0.50, 0.93] | 0.016 | 0.67  [0.49, 0.91] | 0.012 | 0.71  [0.51, 0.98] | 0.037 | 0.68  [0.52, 0.90] | 0.001 | 0.67  [0.51, 0.88] | 0.004 | 0.70  [0.53, 0.94] | 0.017 | 0.74  [0.48, 1.14] | 0.170 | 0.75  [0.48, 1.17] | 0.199 | 0.74  [0.47, 1.17] | 0.202 |
| per 1 SD | 0.85  [0.77, 0.94] | 0.001 | 0.85  [0.76, 0.94] | 0.001 | 0.87  [0.78, 0.97] | 0.010 | 0.85  [0.78, 0.93] | <0.001 | 0.84  [0.77, 0.92] | <0.001 | 0.86  [0.78, 0.94] | 0.002 | 0.88  [0.76, 1.02] | 0.094 | 0.88  [0.76, 1.02] | 0.097 | 0.87  [0.74, 1.02] | 0.083 |
| **uPDI** |  |  |  |  |  |  |  |  |  |  |  |  |  |  |  |  |  |  |
| quintile 5 vs 1 | 1.46  [1.10, 1.94] | 0.010 | 1.50  [1.12, 2.00] | 0.006 | 1.36  [1.00, 1.84] | 0.047 | 1.51  [1.17, 1.94] | 0.001 | 1.55  [1.20, 2.00] | 0.001 | 1.47  [1.12, 1.93] | 0.006 | 1.90  [1.27, 2.86] | 0.002 | 1.95  [1.30, 2.92] | 0.001 | 1.93  [1.26, 2.95] | 0.002 |
| per 1 SD | 1.11  [1.01, 1.22] | 0.025 | 1.12  [1.02, 1.24] | 0.015 | 1.09  [0.99, 1.20] | 0.088 | 1.13  [1.04, 1.23] | 0.004 | 1.13  [1.04, 1.23] | 0.004 | 1.11  [1.02, 1.22] | 0.019 | 1.24  [1.08, 1.43] | 0.003 | 1.27  [1.10, 1.46] | 0.001 | 1.26  [1.09, 1.47] | 0.002 |

The nine dietary pattern scores were divided into quintiles and treated as categorical variables. The Cox regression model derived HRs, 95% CIs, and P-values using a fully adjusted model that accounted for age at first completion of the Oxford WebQ, sex, educational attainment, TDI, family history of cancer, smoking status, average alcohol intake (except aMED & SMD), MET, average energy intake, BMI, WHR T2DM, and additionally adjusted for race in the analysis which excluded individuals with new-onset MAFLD.

MAFLD, metabolic dysfunction-associated fatty liver disease; HR, hazard ratio; CI, confidence interval; aMED, alternate Mediterranean diet; PHD, Planetary Health Diet; SMD, sulfur microbial diet; LCD, Low-carbohydrate Diet; aLCD, animal based Low-carbohydrate Diet; pLCD, plant based Low-carbohydrate Diet; PDI, Plant-based Diet Index; hPDI, healthful Plant-based Diet Index; uPDI, unhealthful Plant-based Diet Index.

**Table S18. Sensitivity analyses for associations of the scores of dietary patterns with the incidence of liver cancer and its subtypes in the UK Biobank cohort.**

|  | **Liver cancer (LC)** | | | | | | **Hepatocellular carcinoma (HCC)** | | | | | | **Intrahepatic cholangiocarcinoma（ICC）** | | | | | |
| --- | --- | --- | --- | --- | --- | --- | --- | --- | --- | --- | --- | --- | --- | --- | --- | --- | --- | --- |
|  | Excluding new-onset individuals with MAFLD | | Excluding participants of others race | | Excluding outcome events occurred during the first 2-years follow up | | Excluding new-onset individuals with MAFLD | | Excluding participants of others race | | Excluding outcome events occurred during the first 2-years follow up | | Excluding new-onset individuals with MAFLD | | Excluding participants of others race | | Excluding outcome events occurred during the first 2-years follow up | |
|  | HR  (95% CI) | P | HR  (95% CI) | P | HR  (95% CI) | P | HR  (95% CI) | P | HR  (95% CI) | P | HR  (95% CI) | P | HR  (95% CI) | P | HR  (95% CI) | P | HR  (95% CI) | P |
| **aMED** |  |  |  |  |  |  |  |  |  |  |  |  |  |  |  |  |  |  |
| quintile 5 vs 1 | 0.54  [0.31, 0.94] | 0.029 | 0.53  [0.30, 0.94] | 0.030 | 0.54  [0.31, 0.97] | 0.037 | 0.57  [0.23, 1.43] | 0.231 | 0.58  [0.23, 1.46] | 0.245 | 0.58  [0.23, 1.47] | 0.254 | 0.46  [0.21, 1.02] | 0.057 | 0.44  [0.19, 1.02] | 0.055 | 0.45  [0.19, 1.07] | 0.072 |
| per 1 SD | 0.90  [0.78, 1.03] | 0.113 | 0.90  [0.78, 1.03] | 0.119 | 0.86  [0.74, 1.00] | 0.045 | 0.91  [0.72, 1.14] | 0.422 | 0.91  [0.73, 1.15] | 0.446 | 0.88  [0.70, 1.12] | 0.314 | 0.83  [0.68, 1.00] | 0.048 | 0.82  [0.68, 1.00] | 0.045 | 0.78  [0.63, 0.96] | 0.019 |
| **PHD** |  |  |  |  |  |  |  |  |  |  |  |  |  |  |  |  |  |  |
| quintile 5 vs 1 | 0.87  [0.58, 1.30] | 0. 502 | 0.88  [0.58, 1.34] | 0.559 | 0.78  [0.50, 1.21] | 0.266 | 1.00  [0.51, 1.93] | 0.990 | 1.06  [0.54, 2.06] | 0.863 | 1.00  [0.50, 1.97] | 0.993 | 0.84  [0.46, 1.52] | 0.559 | 0.82  [0.44, 1.53] | 0.534 | 0.61  [0.30, 1.22] | 0.162 |
| per 1 SD | 0.97  [0.85, 1.11] | 0.670 | 0.98  [0.85, 1.12] | 0.719 | 0.93  [0.81, 1.08] | 0.331 | 0.98  [0.78, 1.23] | 0.845 | 0.99  [0.79, 1.25] | 0.953 | 0.96  [0.76, 1.21] | 0.727 | 0.95  [0.79, 1.15] | 0.622 | 0.95  [0.79, 1.15] | 0.600 | 0.88  [0.72, 1.08] | 0.230 |
| **SMD** |  |  |  |  |  |  |  |  |  |  |  |  |  |  |  |  |  |  |
| quintile 5 vs 1 | 0.80  [0.52, 1.23] | 0.316 | 0.79  [0.51, 1.22] | 0.294 | 0.84  [0.53, 1.34] | 0.464 | 0.96  [0.46, 2.02] | 0.918 | 0.96  [0.46, 2.02] | 0.918 | 0.75  [0.34, 1.64] | 0.464 | 0.79  [0.43, 1.47] | 0.464 | 0.77  [0.40, 1.47] | 0.425 | 1.06  [0.53, 2.10] | 0.867 |
| per 1 SD | 0.91  [0.80, 1.04] | 0.162 | 0.90  [0.79, 1.03] | 0.127 | 0.91  [0.80, 1.05] | 0.207 | 0.95  [0.77, 1.18] | 0.662 | 0.95  [0.77, 1.18] | 0.653 | 0.92  [0.74, 1.14] | 0.459 | 0.91  [0.76, 1.10] | 0.324 | 0.89  [0.74, 1.08] | 0.249 | 0.94  [0.76, 1.15] | 0.518 |
| **LCD** |  |  |  |  |  |  |  |  |  |  |  |  |  |  |  |  |  |  |
| quintile 5 vs 1 | 1.11  [0.72, 1.72] | 0.624 | 1.06  [0.68, 1.63] | 0.809 | 1.18  [0.74, 1.87] | 0.484 | 1.32  [0.68, 2.58] | 0.411 | 1.27  [0.65, 2.48] | 0.485 | 1.24  [0.62, 2.48] | 0.552 | 1.01  [0.54, 1.91] | 0.967 | 0.95  [0.50, 1.78] | 0.867 | 1.17  [0.59, 2.31] | 0.648 |
| per 1 SD | 0.98  [0.86, 1.12] | 0.785 | 0.97  [0.85, 1.11] | 0.658 | 0.98  [0.85, 1.13] | 0.798 | 0.99  [0.79, 1.23] | 0.915 | 0.99  [0.79, 1.24] | 0.921 | 0.96  [0.76, 1.21] | 0.735 | 0.99  [0.82, 1.19] | 0.900 | 0.98  [0.80, 1.17] | 0.714 | 1.02  [0.83, 1.24] | 0.882 |
| **aLCD** |  |  |  |  |  |  |  |  |  |  |  |  |  |  |  |  |  |  |
| quintile 5 vs 1 | 1.10  [0.72, 1.69] | 0.663 | 1.04  [0.68, 1.60] | 0.849 | 1.16  [0.72, 1.86] | 0.540 | 1.22  [0.64, 2.33] | 0.554 | 1.20  [0.63, 2.30] | 0.579 | 1.17  [0.60, 2.30] | 0.645 | 1.12  [0.60, 2.08] | 0.730 | 1.01  [0.54, 1.89] | 0.976 | 1.40  [0.68, 2.86] | 0.357 |
| per 1 SD | 1.00  [0.88, 1.14] | 0.995 | 0.98  [0.86, 1.12] | 0.790 | 0.99  [0.86, 1.14] | 0.872 | 1.00  [0.81, 1.25] | 0.967 | 1.00  [0.80, 1.25] | 0.997 | 0.98  [0.78, 1.24] | 0.882 | 1.01  [0.84, 1.22] | 0.884 | 0.99  [0.81, 1.19] | 0.855 | 1.03  [0.84, 1.26] | 0.782 |
| **pLCD** |  |  |  |  |  |  |  |  |  |  |  |  |  |  |  |  |  |  |
| quintile 5 vs 1 | 0.96  [0.64, 1.46] | 0.861 | 1.00  [0.65, 1.52] | 0.985 | 0.93  [0.60, 1.44] | 0.741 | 0.96  [0.43, 2.15] | 0. 916 | 1.03  [0.45, 2.35] | 0.940 | 0.85  [0.36, 2.03] | 0.714 | 0.87  [0.51, 1.50] | 0.622 | 0.89  [0.51, 1.54] | 0.681 | 0.82  [0.46, 1.47] | 0.513 |
| per 1 SD | 0.96  [0.85, 1.10] | 0.592 | 0.98  [0.85, 1.11] | 0.711 | 0.99  [0.86, 1.14] | 0.841 | 1.05  [0.84, 1.31] | 0.685 | 1.06  [0.85, 1.33] | 0.589 | 1.02  [0.80, 1.28] | 0.899 | 0.90  [0.75, 1.08] | 0.242 | 0.90  [0.75, 1.09] | 0.288 | 0.93  [0.76, 1.13] | 0.448 |
| **PDI** |  |  |  |  |  |  |  |  |  |  |  |  |  |  |  |  |  |  |
| quintile 5 vs 1 | 1.08  [0.73, 1.61] | 0.691 | 1.13  [0.75, 1.68] | 0.562 | 1.13  [0.74, 1.73] | 0.581 | 1.14  [0.61, 2.15] | 0.679 | 1.20  [0.63, 2.27] | 0.576 | 1.35  [0.70, 2.59] | 0.366 | 0.91  [0.49, 1.68] | 0.763 | 0.94  [0.50, 1.77] | 0.857 | 0.89  [0.45, 1.75] | 0.731 |
| per 1 SD | 1.01  [0.88, 1.15] | 0.916 | 1.02  [0.89, 1.17] | 0.760 | 1.02  [0.88, 1.18] | 0.778 | 0.96  [0.76, 1.20] | 0.707 | 0.97  [0.77, 1.22] | 0.796 | 1.01  [0.80, 1.28] | 0.949 | 0.99  [0.82, 1.20] | 0.937 | 1.01  [0.83, 1.23] | 0.926 | 1.00  [0.81, 1.23] | 0.997 |
| **hPDI** |  |  |  |  |  |  |  |  |  |  |  |  |  |  |  |  |  |  |
| quintile 5 vs 1 | 0.91  [0.60, 1.38] | 0.654 | 0.97  [0.63, 1.49] | 0.892 | 0.99  [0.64, 1.52] | 0.947 | 1.10  [0.56, 2.17] | 0.775 | 1.12  [0.57, 2.20] | 0.742 | 1.05  [0.52, 2.08] | 0.900 | 0.83  [0.45, 1.51] | 0.536 | 0.94  [0.50, 1.75] | 0.845 | 0.95  [0.50, 1.79] | 0.870 |
| per 1 SD | 0.97  [0.84, 1.12] | 0.661 | 0.99  [0.86, 1.14] | 0.864 | 0.97  [0.83, 1.13] | 0.710 | 0.98  [0.77, 1.24] | 0.836 | 0.98  [0.77, 1.24] | 0.849 | 0.93  [0.72, 1.19] | 0.548 | 0.94  [0.77, 1.15] | 0.564 | 0.98  [0.80, 1.20] | 0.824 | 0.97  [0.78, 1.20] | 0.747 |
| **uPDI** |  |  |  |  |  |  |  |  |  |  |  |  |  |  |  |  |  |  |
| quintile 5 vs 1 | 1.51  [1.00, 2.30] | 0.051 | 1.55  [1.02, 2.35] | 0.040 | 1.64  [1.05, 2.58] | 0.030 | 1.29  [0.67, 2.47] | 0.449 | 1.30  [0.68, 2.49] | 0.436 | 1.52  [0.77, 3.01] | 0.226 | 1.63  [0.89, 2.99] | 0.114 | 1.70  [0.92, 3.11] | 0.088 | 1.79  [0.92, 3.51] | 0.088 |
| per 1 SD | 1.14  [1.00, 1.31] | 0.055 | 1.16  [1.01, 1.33] | 0.034 | 1.18  [1.02, 1.36] | 0.027 | 1.09  [0.87, 1.37] | 0.439 | 1.10  [0.88, 1.38] | 0.400 | 1.16  [0.92, 1.46] | 0.220 | 1.17  [0.96, 1.41] | 0.114 | 1.20  [0.99, 1.45] | 0.070 | 1.22  [0.99, 1.51] | 0.057 |

The nine dietary pattern scores were divided into quintiles and treated as categorical variables. The Cox regression model derived HRs, 95% CIs, and P-values using a fully adjusted model that accounted for age at first completion of the Oxford WebQ, sex, educational attainment, TDI, family history of cancer, smoking status, average alcohol intake (except aMED & SMD), MET, average energy intake, BMI, WHR T2DM, and additionally adjusted for race in the analysis which excluded individuals with new-onset MAFLD.

MAFLD, metabolic dysfunction-associated fatty liver disease; HR, hazard ratio; CI, confidence interval; aMED, alternate Mediterranean diet; PHD, Planetary Health Diet; SMD, sulfur microbial diet; LCD, Low-carbohydrate Diet; aLCD, animal based Low-carbohydrate Diet; pLCD, plant based Low-carbohydrate Diet; PDI, Plant-based Diet Index; hPDI, healthful Plant-based Diet Index; uPDI, unhealthful Plant-based Diet Index.
